# Comprehensive functional enrichment analysis of male infertility

**DOI:** 10.1038/s41598-017-16005-0

**Published:** 2017-11-17

**Authors:** Seyed Morteza Razavi, Marjan Sabbaghian, Mahdi Jalili, Adeleh Divsalar, Olaf Wolkenhauer, Ali Salehzadeh-Yazdi

**Affiliations:** 1 Department of Cell and Molecular Biology, Faculty of Biological Sciences, Kharazmi University, Tehran, Iran; 2 Department of Andrology, Reproductive Biomedicine Research Center, Royan Institute for Reproductive Biomedicine, ACECR, Tehran, Iran; 3 Hematology, Oncology and SCT Research Center, Tehran University of Medical Sciences, Tehran, Iran; 4 Department of Systems Biology and Bioinformatics, University of Rostock, 18051 Rostock, Germany

## Abstract

Spermatogenesis is a multifactorial process that forms differentiated sperm cells in a complex microenvironment. This process involves the genome, epigenome, transcriptome, and proteome to ensure the stability of the spermatogonia and supporting cells. The identification of signaling pathways linked to infertility has been hampered by the inherent complexity and multifactorial aspects of spermatogenesis. Systems biology is a promising approach to unveil underlying signaling pathways and genes and identify putative biomarkers. In this study, we analyzed thirteen microarray libraries of infertile humans and mice, and different classes of male infertility were compared using differentially expressed genes and functional enrichment analysis. We found regulatory processes, immune response, glutathione transferase and muscle tissue development to be among the most common biological processes in up-regulated genes, and genes involved in spermatogenesis were down-regulated in maturation arrest (MArrest) and oligospermia cases. We also observed the overexpression of genes involved in steroid metabolism in post-meiotic and meiotic arrest. Furthermore, we found that the infertile mouse model most similar to human MArrest was the *Dazap1* mutant mouse. The results of this study could help elucidate features of infertility etiology and provide the basis for diagnostic markers.

## Introduction

Infertility is defined as the inability to have children after one year of unprotected sexual intercourse^1^. Ten to fifteen percent of couples face infertility, which is related to male factors in almost 50% of cases^2^. The most common causes of male infertility are varicocele (37–40%), endocrine disorders (>20%), genital duct infection (8–35%), testicular defects (9%), genetic problems (15–30%), antisperm antibodies (8–19%), and idiopathic male infertility (15–25%)^3–5^.

Semen deficiencies in male infertility are often characterized as 1) oligospermia, in which there are fewer than 15 million sperm cells per milliliter, 2) azoospermia, which is the absence of sperm in ejaculate and which can be categorized into two major classes, obstructive azoospermia (OA) and non-obstructive azoospermia (NOA), 3) teratospermia, a condition in which less than 4% of sperm are morphologically normal, and 4) asthenospermia, which is when sperm have motility problems^6^. Idiopathic male infertility is a complicated condition with abnormal semen parameters that probably has a genetic basis^7,8^. Some cases are classified as “unexplained male infertility,” in which all characteristics of routine semen analysis and sexual history are normal^9^. Despite abundant studies, the origins of many infertility cases are still not known because spermatogenesis is a multifactorial and complex process. The cause of 21–29% of azoospermia cases is related to genetic factors, and 12–41% of azoospermia cases are idiopathic azoospermia^10^. The genetic basis of azoospermia involves numerous causes, such as abnormality, single and multiple gene disorders and epigenetics, and Y chromosome defects have a major role in male infertility^10^.

NOA patients go through four stages, such as pre-meiotic arrest (PreMA), meiotic arrest (MA), post-meiotic arrest (PostMA) and sertoli cell only syndrome (SCOS)^11^. Unlike NOA cases, we do not have enough information about the transcriptome of testis tissue for oligospermia and teratospermia cases because they are not a candidate for testis biopsy. Our knowledge about these cases should be based on the genome, the transcriptome of sperm cells, the genome of somatic cells of infertile men and the testis tissue of infertile mouse mutants^12^. Furthermore, unlike oligospermia and teratospermia cases, NOA cases are unable to create sperm, so we do not know about the transcriptome and proteome of NOA sperm^13^.

Based on current knowledge, the spermatogenesis process is regulated by 1500 to 2000 genes, and every alteration in these genes may disturb fertility^12,14^. Several studies have investigated the biology of spermatogenesis and identified many key genes involved in spermatogenesis pathways. There are some comprehensive reviews about the dependency among the genome, epigenome, transcriptome and proteome^15^ and the genes and pathways involved in male infertility^8,12,16^. High throughput technologies, such as gene expression profiling assays, have been extensively applied to investigate the molecular mechanisms associated with male infertility^17^. In 2006, in one of the first microarray experiments on SCOS and MA cases, 10 novel genes were identified that had been down-regulated in male infertility cases^18^. In 2008, Okada *et al*. revealed differentially expressed genes (DEGs) in NOA cases, investigated the top 10 biological processes (BP) of gene ontology (GO) terms for separate up- and down-regulated gene lists, and suggested some novel therapeutic targets for NOA treatment^19^. A transcriptome analysis of NOA and hypospermatogenesis (HS) (with and without AZFc [azoospermia factor c] region deletion) by Gatta *et al*. revealed that the transcripts of all cases with AZFc deletion were clustered together independently from the phenotype of testes (SCOS or HS). Furthermore, the transcripts of half of the idiopathic HS cases were clustered with the AZFc deletion cases, and many of the genes with post-meiotic functions were down-regulated in AZFc deletion cases^20^. In 2010, Saito *et al*. studied the microarray data of *Ing2* knockdown (KD) mouse testes and showed that *Ing2* plays a crucial role in spermatogenesis^21^. In 2013, Malcher *et al*. extracted the DEGs of PreMA, MA, PostMA and SCOS and focused on the expression of genes involved in the immune system^22,23^. In another study in 2015, Bansal *et al*. analyzed the GO of mixed up- and down-regulated genes in sperm gene expression profiles of idiopathic oligospermia and asthenospermia^24^. A bioinformatics analysis of four microarray datasets of NOA testes was conducted by Ansari-Pour *et al*. in 2016. They reconstructed a protein-protein interaction network of spermatogenic failure genes with a Y-centric focus base of DEGs^25^. A gene set enrichment analysis (GSEA) establishes whether an *a priori* defined set of genes shows statistically significant differences between two biological states or phenotypes^26^.

In this study, we elicited the DEGs involved in each type of human male infertility and multiple genes involved in certain infertile mouse mutants and several stages of arresting in meiosis and SCOS. Then GO and KEGG pathway analyses were performed on the DEGs. Furthermore, we performed a GSEA for each type of male infertility for humans and mice to discover the most important gene sets in male infertility. This study is a step toward finding a diagnostic biomarker for male infertility and could help explain the etiology of male infertility.

## Results

### DEGs and pathway analysis in maturation arrest azoospermia (MArrest), oligospermia and teratospermia

We extracted 597, 154 and 283 up-regulated genes and 525, 144 and 292 down-regulated genes from the libraries of MArrest, oligospermia and teratospermia (MArrest-oligo-terato-spermia), respectively (Supplementary Table S1). We found 26 up-regulated miRNAs for MArrest (*miR-15A*, *miR-18a*, *miR-21*, *miR-23b*, *miR-27b*, *miR29c*, *miR-30e*, *miR-31*, *miR-32*, m*iR-99a*, *miR-99AHG*, *miR-128-1*, *miR-107*, *miR-145*, *miR-154*, *miR-186*, *miR-197*, *miR-199a-2*, *miR-214*, *miR-218-1*, *miR-503*, *miR-509*, *miR-LET7A2*, *miR-LET7C*, *miR-LET7F1* and *miR-LET7G*), two up-regulated miRNAs for teratospermia (miR-9-2 and miR-181A2HG) and one down-regulated miRNA for teratospermia (*miR-6805*). There was no common up-regulated gene among MArrest-oligo-terato-spermia disorders, but five down-regulated genes (*CLGN*, *KRT23*, *PCYT2, SMCP* and *SPINK2*) were common among MArrest-oligo-terato-spermia disorders.

**Figure 1:**
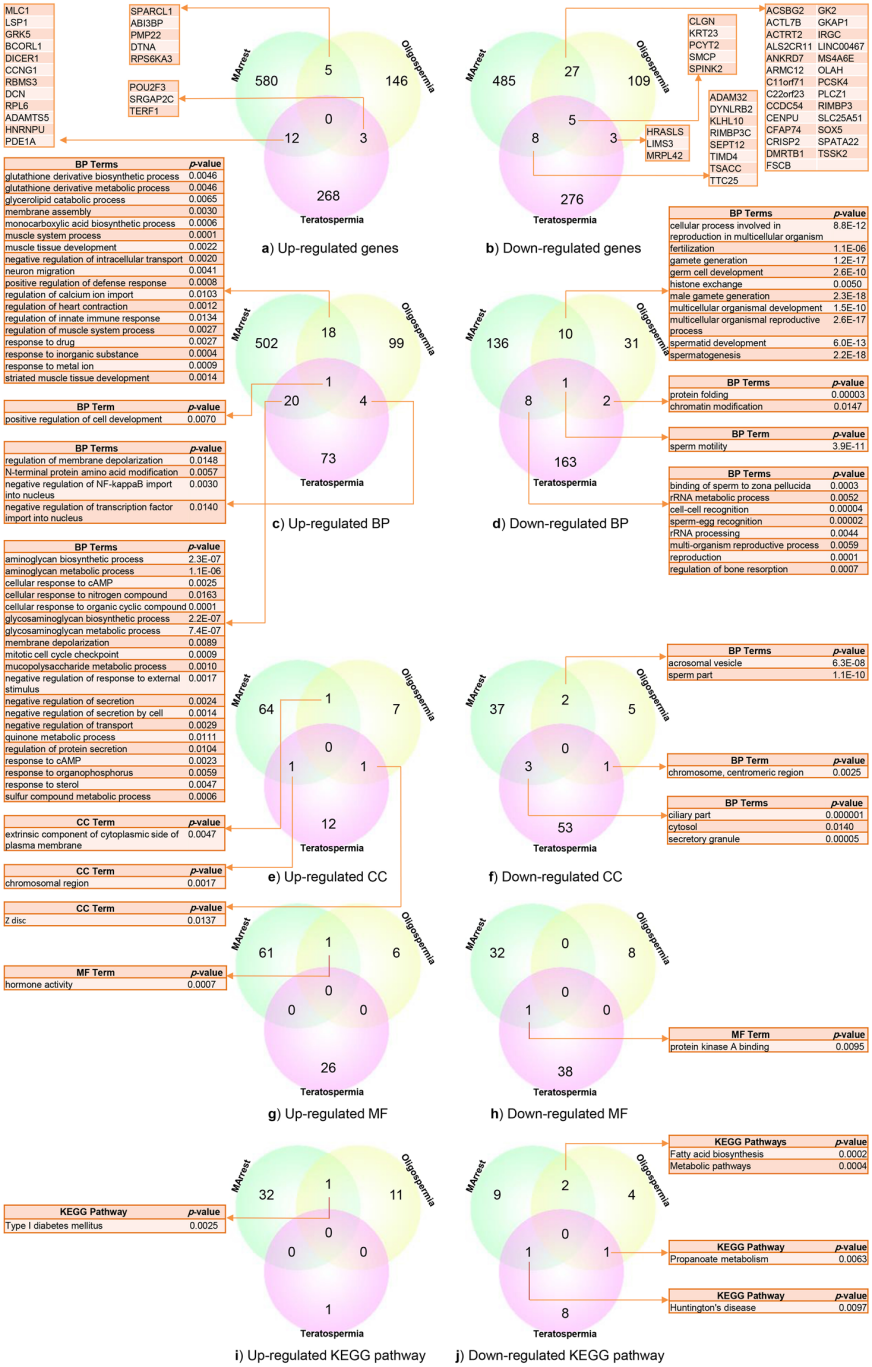
Venn diagram of similarities of DEGs, GO, KEGG pathway enrichment analysis between MArrest-oligo-terato-spermia. (**a**) Common up-regulated genes which the most similarity was between MArrest-terato-spermia (12 mutual genes). (**b**) Common down-regulated genes. The most similarity was between MArrest-oligo-spermia (27 mutual genes). (**c**) Common up-regulated BP that the common genes between MArrest-oligo-spermia were about regulatory process, immune system process and muscle tissue development. (**d**) Common down-regulated BP which mostly was between MArrest- oligo-spermia by spermatogenesis process. (**e**) Mutual up-regulated CC which common CC between MArrest- oligo-spermia related to plasma membrane. (**f**) Common down-regulated CC. (**g**,**h**) Common up- and down-regulated MF. (**i**,**j**) Common up- and down-regulated KEGG pathways.

In up-regulated genes, the maximum similarity was between MArrest and teratospermia, with 12 common genes among 880 genes (0.014%) (*MLC1, LSP1, GRK5, BCORL1, DICER1, CCNG1, RBMS3, DCN, RPL6, ADAMTS5, HNRNPU* and *PDE1A)*. Five genes *(SPARCL1, ABI3BP, PMP22, DTNA* and *RPS6KA3*) were common between MArrest and oligospermia among 751 genes (0.007%), and three genes (*POU2F3, SRGAP2C* and *TERF1*) were common between oligospermia and teratospermia among 437 genes (0.007%), as shown in Fig. 1a.

In down-regulated genes, five genes among 960 genes (0.005%) were common in all three MArrest-oligo-terato-spermia disorders. The maximum similarity was between MArrest and oligospermia, with 27 common genes among 668 genes (0.04%) (*ACSBG2, ACTL7B, ACTRT2, ALS2CR11, ANKRD7, ARMC12, C11orf71, C22orf23, CCDC54, CENPU, CFAP74, CRISP2, DMRTB1, FSCB, GK2, GKAP1, IRGC, LINC00467, MS4A6E, OLAH, PCSK4, PLCZ1, RIMBP3, SLC25A51, SOX5, SPATA22* and *TSSK2*). Eight genes among 817 genes (0.01%) (*ADAM32, DYNLRB2, KLHL10, RIMBP3C, SEPT12, TIMD4, TSACC* and *TTC25*) were common between MArrest and teratospermia, and three genes among 435 genes (0.007%) (*HRASLS, LIMS3* and *MRPL42*) were common between oligospermia and teratospermia. The common down-regulated genes are listed in Fig. 1b.

We found 541 BP terms for MArrest up-regulated genes, 122 BP terms for oligospermic up-regulated genes and 98 BP terms for teratospermic up-regulated genes. There were 155, 44 and 147 common BP terms for MArrest, oligospermia and teratospermia down-regulated genes, respectively (*p*-value ≤ 0.01) (Supplementary Table S2).

In the up-regulated gene lists for BPs, one BP was common among all MArrest-oligo-terato-spermia disorders, 18 BPs were common between MArrest and oligospermia, 20 BP terms were common between MArrest and teratospermia, and four BPs were common between oligospermia and teratospermia, as can be seen in Fig. 1c.

As observed in Fig. 1d, in the down-regulated gene lists for BPs, one BP term was common among all MArrest-oligo-terato-spermia disorders, 10 BP terms were common between MArrest and oligospermia, eight BPs were common between MArrest and teratospermia, and two BP terms were common between oligospermia and teratospermia.

We investigated 66, 9 and 14 cellular component (CC) terms for up-regulated genes and 42, 8 and 57 CC terms for down-regulated genes of MArrest, oligospermia and teratospermia, respectively (Supplementary Table S2). In the up-regulated gene lists for CC, as depicted in Fig. 1e, one CC term was common between MArrest and oligospermia, one CC was common between MArrest and teratospermia, and one CC was common between oligospermia and teratospermia. In the down-regulated gene lists for CC, two CC terms were common between MArrest and oligospermia, three CCs were common between MArrest and teratospermia, and one CC term was common between oligospermia and teratospermia (see Fig. 1f).

We found 62, 7 and 26 molecular function (MF) terms for up-regulated genes and 33, 8 and 39 MF terms for down-regulated genes of MArrest, oligospermia and teratospermia, respectively (Supplementary Table S2). In the up-regulated gene lists for MF,as shown in Fig. 1g, one MF term was common among MArrest-oligo-spermia disorders. In the down-regulated gene lists for MF, one MF term was common between MArrest and teratospermia (see Fig. 1h). In the up-regulated gene lists for the KEGG pathway, as depicted in Fig. 1i, one pathway was common between MArrest and oligospermia (*type I diabetes mellitus*). In the down-regulated gene lists for the KEGG pathway, as illustrated in Fig. 1j, two pathways (*fatty acid biosynthesis* and *metabolic pathways*) were common between MArrest and oligospermia, one pathway was common between oligospermia and teratospermia (*Propanoate metabolism*), and one pathway was common between MArrest and teratospermia (*Huntington’s disease*).

### DEGs and pathway analysis in PostMA, MA and SCOS

**Figure 2:**
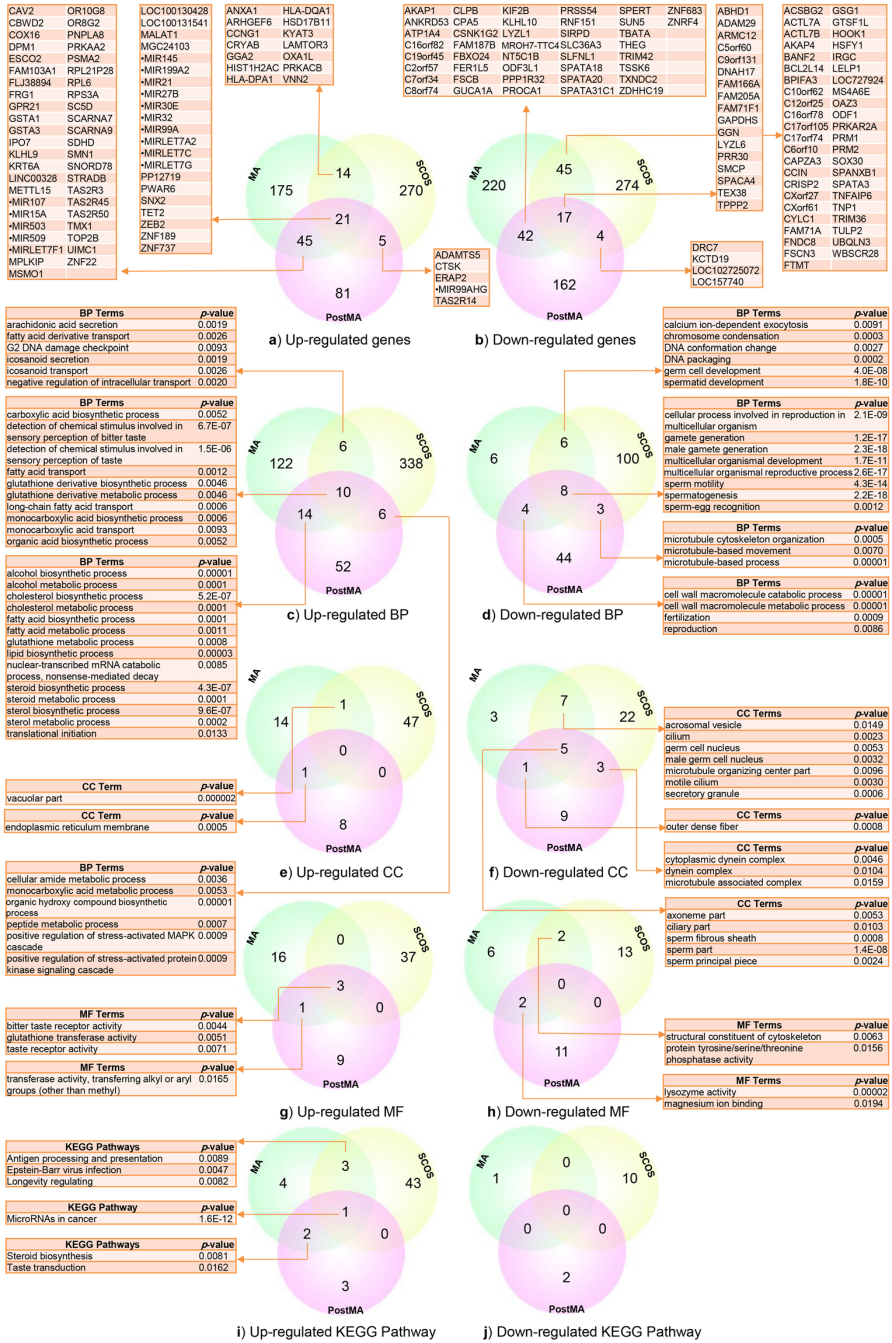
Venn diagram of similarities of DEGs, GO, KEGG pathway enrichment analysis between PostMA, MA and SCOS. (**a**) Common up- regulated genes with 21 mutual genes between all three groups. (**b**) Common down-regulated genes which 17 mutual genes were between all groups and 42 common genes were between PostMA and MA. (**c**,**d**) Common up and down-regulated BP, 10 BP terms were common between all PostMA, MA and SCOS, 14 BP terms were common between MA and PostMA, and 6 BP term was common between PostMA and SCOS. In down-regulated gene lists for BP, 8 BP terms were common between all PostMA, MA and SCOS. (**e**,**f**) In up-regulated CC, no common terms found. In down-regulated gene lists for CC, 5 CC terms were common between all PostMA, MA and SCOS and 7 CC terms were common between MA and PostMA. (**g**,**h**) Common up- and down-regulated MF. (**i**,**j**) Common up and down-regulated KEGG pathways.

We extracted 152, 255 and 310 top up-regulated genes and 163, 255 and 309 top down-regulated genes from the libraries of different stages of NOA: PostMA, MA and SCOS, respectively (Supplementary Table S3). For the up-regulated genes, 21 genes among 717 genes (0.029%) were common among PostMA, MA and SCOS, and half of these genes were miRNA (*LOC100130428, LOC100131541, MALAT1, MGC24103, miR-145*, *miR-199a-2*, *miR-21, miR-27b, miR-30e, miR-32*, *miR-99a*, *miR-LET7A2*, *miR-LET7C*, *miR-LET7G, PP12719, PWAR6, SNX2, TET2, ZEB2, ZNF189* and *ZNF737*). The maximum number of common genes was found between PostMA and MA, with 45 up-regulated genes among 407 genes (0.11%). Fourteen up-regulated genes among 565 genes (0.025%) were common between MA and SCOS, and five up-regulated genes among 462 genes (0.011%) were common between PostMA and SCOS. The common up-regulated genes have been shown in Fig. 2a. In down-regulated genes, 17 genes among 727 genes (0.023%) were common among PostMA, MA and SCOS, 45 genes among 564 genes (0.08%) were common between MA and SCOS, and 42 genes among 418 genes (0.1%) were common between PostMA and MA, as shown in Fig. 2b. We found 82 BP terms for PostMA up-regulated genes, 152 BP terms for MA up-regulated genes and 360 BP terms for SCOS up-regulated genes. We also discovered 59, 24 and 117 BP terms in PostMA, MA and SCOS for down-regulated genes, respectively (*p*-value ≤ 0.01) (Supplementary Table 4). In the up-regulated gene lists for BPs, 10 BP terms were common among PostMA, MA and SCOS, 14 BP terms were common between PostMA and MA, six BP terms were common between MA and SCOS, and six BP terms were common between PostMA and SCOS. The common BP terms for up-regulated genes are illustrated in Fig. 2c. As shown in Fig. 2d, in the down-regulated gene lists for BPs, eight BP terms were common among PostMA, MA and SCOS, four BP terms were common between PostMA and MA, six BP terms were common between MA and SCOS, and three BP terms were common between PostMA and SCOS. We investigated 9, 16 and 48 CC terms for up-regulated genes and 18, 16 and 37 CC terms for down-regulated genes of PostMA, MA and SCOS, respectively (Supplementary Table S4). In the up-regulated gene lists for CC, we found one common CC term between PostMA and MA and one common CC term between MA and SCOS (see Fig. 2e). As Fig. 2f indicates, in the down-regulated gene lists for CC, 5 CC terms were common among PostMA, MA and SCOS, seven CC terms were common between MA and SCOS, three CC terms were common between PostMA and SCOS, and one CC term was common between PostMA and MA. We found 13, 20 and 40 MF terms for up-regulated genes and 13, 10 and 15 MF terms for down-regulated genes of PostMA, MA and SCOS, respectively (Supplementary Table S4). In the up-regulated gene lists for MF, we observed three common MF terms among PostMA, MA and SCOS, and one MF term was common between PostMA and MA (see Fig. 2g). In the down-regulated gene lists for MF, two MF terms were common between PostMA and MA, and two MF terms were common between MA and SCOS, as shown in Fig. 2h. We found 6, 9 and 47 KEGG pathways for up-regulated genes and 2, 1 and 10 KEGG pathways for down-regulated genes (Supplementary Table S4). In up-regulated genes, we observed common miRNAs among PostMA, MA and SCOS that are involved in cancer. Three pathways were common between MA and SCOS for up-regulated genes (*antigen processing and presentation*, *Epstein-Barr virus infection* and *longevity regulating*), and the pathways of steroid biosynthesis and taste transduction were common between PostMA and MA up-regulated genes (see Fig. 2i). As can be seen in Fig. 2j, we did not find any common pathways for down-regulated genes.

### Comparison of infertile human and mouse

We compared nine types of infertility in male humans and mice, including MArrest, oligospermia and teratospermia in humans and *Ing2* Knockout (KO), *Bcl6* KD, *Etv5* KD, *Pou3f1* KD, *Ikbkap* KO and *Dazap1* mutant in mice. As shown in Table 1, the higher number of common up-regulated genes was found between *Etv5* KD and *Pou3f1* KD, with 53 genes, *Bcl6b* KD and *Etv5* KD, with 19 genes, *Bcl6b* KD and *Pou3f1* KD, with 14 genes, MArrest and teratospermia, with 12 genes, and MArrest and *Ing2* KO, with nine common genes. Additionally, the *Arpc1b* gene was up-regulated in *Bcl6b, Etv5, Pouf31* and *Dazap1* KD, and *Alcam* was up-regulated in *Bcl6b, Etv5, Pouf31* and *Ing2* KD infertile mice. The highest number of common down-regulated genes was found between MArrest and oligospermia, with 32 genes, MArrest and the *Dazap1* mutant, with 21 genes, MArrest and teratospermia, with 13 genes, and MArrest and *Ikbkap* KO, with 8 common genes. Three down-regulated genes (*PLCZ1*, *TSSK2* and *ANKRD7*) were common between MArrest, oligospermia and the *Dazap1* mouse mutant. *DMRTB1* was a down-regulated common gene among MArrest, oligospermia and *Ikbkap* KO, *GPR137B* was common among teratospermia, *Bcl6b* and *Pou3f1* KD, and *LMNB2* was common among *Bcl6b*, *Etv5* and *Pou3f1* KD infertile mice (Table 1 and Table 2).

**Table 1:**
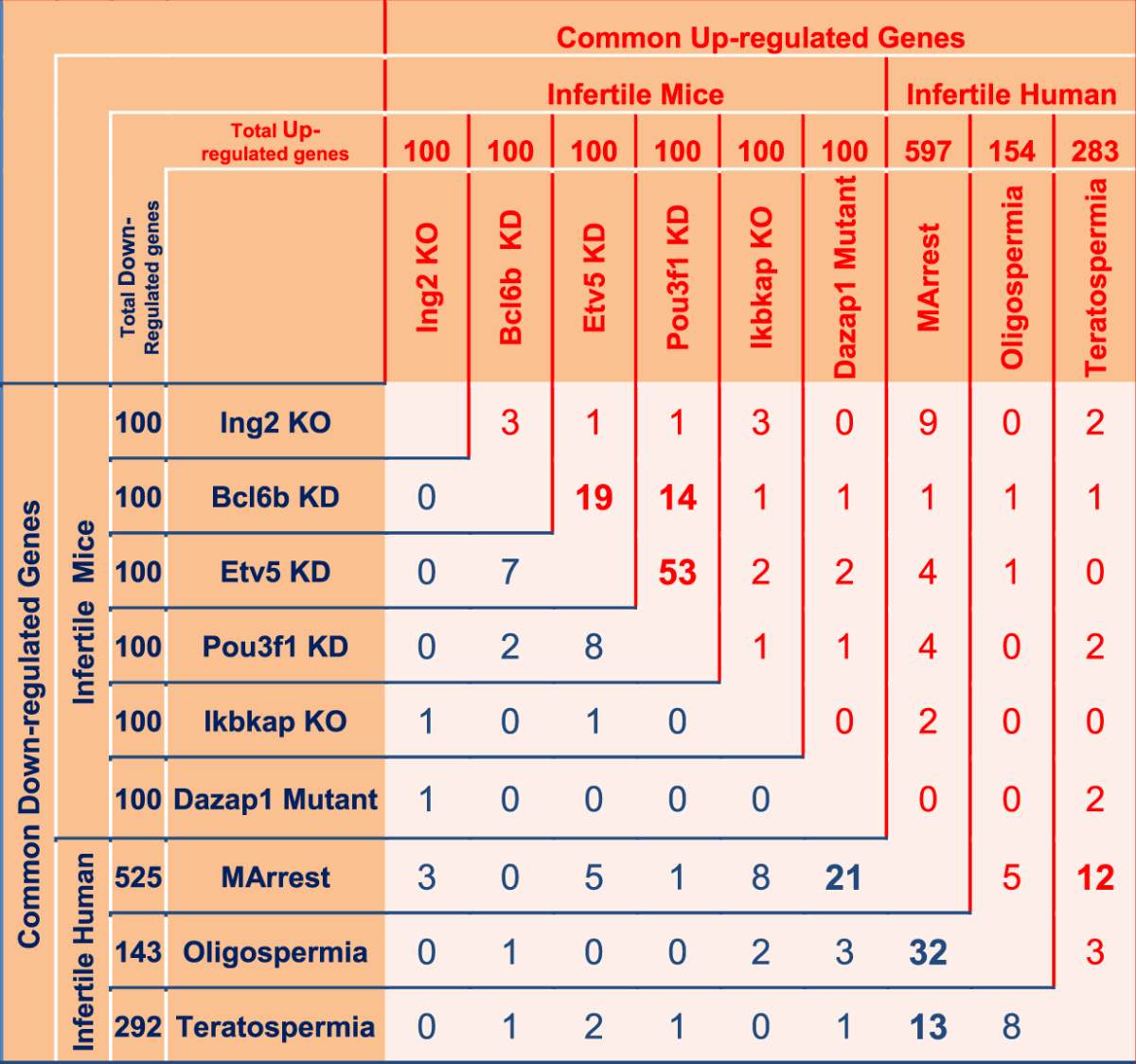
The number of common up- and down-regulated genes between infertile male human and mouse models.The red numbers are common up- regulated genes and blues are common down-regulated genes. In up-regulated genes the most similarity was between *Etv5* and *Pou3f1* KD (53 genes) and in down-regulated genes the most common genes was between MArrest and oligospermia (32 genes). *Note*: KO = Knockout, KD** = **Knockdown; MArrest = maturation arrest azoospermia.

**Table 2:** Common up- and down-regulated genes between infertile male human and mouse models.

Male infertility In Human and Mouse		Count	Common Up-regulated Genes
**Etv5 KD**	**Pou3f1 KD**	**53**	ACVR2A- AI256396- **ALCAM-** ANKRD29- ARF6- **ARPC1B**- ASPH- ATP1B1- BCL2- CAMK2N1- CASP3- CCNG2- CCNH- CCNY- CHMP3- CRLS1- D030029J20RIK- EIF4E2- EPS15- EXOC4- FAM8A1- FBN1- HIF1A- IKBKG- KCTD14- LAGE3- LGALS8- MACROD2- MFSD1- PHEX- PIGN- PIK3IP1- PKIA- PLSCR3- POLR3E- PPARGC1A- PPP1R21- PWP2- QK- RRAGD- SDPR- SH3GLB1- SURF4- TCFL5- TK2- TNC- TNRC6A- TRAPPC2- TUBB2B- UBE2Q2- USP22- YWHAH- ZFP667
**Bcl6b KD**	**Etv5 KD**	**19**	2810043O03RIK- **ALCAM-** ANKRD29- **ARPC1B**- CAMK2N1- CCNY- CRLS1- D030029J20RIK- E330037M01RIK- EXOC4- ITGB8- MALAT1- MMP13- PHEX- PPARGC1A- QK- TCFL5- TK2- ZBTB20
**Bcl6b KD**	**Pou3f1 KD**	**14**	**ALCAM-** ANKRD29- **ARPC1B**- CAMK2N1- CCNY- CRLS1- D030029J20RIK- EXOC4- PHEX- PPARGC1A- QK- TCFL5- TK2- ZFP292-
**MArrest**	**Teratospermia**	**12**	ADAMTS5- BCORL1- CCNG1- DCN- DICER1- GRK5- HNRNPU- LSP1- MLC1- PDE1A- RBMS3- RPL6
**MArrest**	**Ing2 KO**	**9**	ACSS3- COMMD6- CYP11A1- DCN- HSD17B3- HSD3B1- HSPA8- MGARP- MSMO1-
**MArrest**	**Oligospermia**	**5**	ABI3BP- DTNA- PMP22- RPS6KA3- SPARCL1
**MArrest**	**Etv5 KD**	**4**	CASK- MALAT1- SC5D- TUBB2B
**MArrest**	**Pou3f1 KD**	**4**	HMGB1- IGFBP5- RDX- TUBB2B
**Ikbkap KO**	**Ing2 KO**	**3**	ADH1- BCAT2- LIP
**Oligospermia**	**Teratospermia**	**3**	POU2F3- SRGAP2C- TERF1
**Bcl6b KD**	**Ing2 KO**	**3**	**ARPC1B**- LRP1- MID1
**MArrest**	**Ikbkap KO**	**2**	ELAVL3- PAPSS2
**Dazap1 Mutant**	**Etv5 KD**	**2**	**ALCAM**- PLAGL1
**Dazap1 Mutant**	**Teratospermia**	**2**	SLC26A3- SORBS1
**Etv5 KD**	**Ikbkap KO**	**2**	IGHM- ITGB
**Ing2 KO**	**Teratospermia**	**2**	DCN- GPM6B
**Pou3f1 KD**	**Teratospermia**	**2**	COL11A1- LPAR4
**MArrest**	**Bcl6b KD**	**1**	MALAT1
**Bcl6b KD**	**Dazap1 Mutant**	**1**	**ALCAM**
**Bcl6b KD**	**Ikbkap KO**	**1**	ITGB8
**Bcl6b KD**	**Oligospermia**	**1**	CUX1
**Bcl6b KD**	**Teratospermia**	**1**	FAM172A
**Dazap1 Mutant**	**Pou3f1 KD**	**1**	**ALCAM**
**Etv5 KD**	**Ing2 KO**	**1**	**ARPC1B**
**Etv5 KD**	**Oligospermia**	**1**	SNX13
**Ikbkap KO**	**Pou3f1 KD**	**1**	TCL1
**Ing2 KO**	**Pou3f1 KD**	**1**	**ARPC1B**
**Male infertility In Human and Mouse**		**Count**	**Common Down-regulated Genes**
**MArrest**	**Oligospermia**	**32**	ACSBG2- ACTL7B- ACTRT2- ALS2CR11- **ANKRD7**- ARMC12- C11ORF71- C22ORF23- CCDC54- CENPU- CFAP74- **CLGN**- CRISP2- DMRTB1- FSCB- GK2- GKAP1- IRGC- **KRT23**- LINC00467- MS4A6E- OLAH- PCSK4- **PCYT2**- **PLCZ1**- RIMBP3- SLC25A51- **SMCP**- SOX5- SPATA22- **SPINK2**- **TSSK2**
**MArrest**	**Dazap1 Mutant**	**21**	AKAP4- **ANKRD7**- APOBEC4- DDI1- DYDC1- GALNTL5- GTSF1L- HMGB4- IQCF5- KIF2B- LYZL1- NT5C1B- ODF1- ODF3- **PLCZ1**- PRM1- PRR30- SPAG6- STAT4- **TSSK2**- ZNRF4
**MArrest**	**Teratospermia**	**13**	ADAM32- **CLGN**- DYNLRB2- KLHL10- **KRT23**- **PCYT2**- RIMBP3C- SEPTIN12- **SMCP**- **SPINK2**- TIMD4- TSACC- TTC25
**MArrest**	**Ikbkap KO**	**8**	ANO1- DMRTB1- LCA5L- MARCH11- PPP3R2- SPATA4- SPATS1- SUN3
**Etv5 KD**	**Pou3f1 KD**	**8**	AGPAT3- EVI2A- GALNT10- LMNB2- PTPRE- SCLY- SEMA7A- TM4SF1
**Oligospermia**	**Teratospermia**	**8**	**CLGN**- HRASLS- **KRT23**- LIMS3-LOC440895- MRPL42- **PCYT2**- **SMCP**- **SPINK2**-
**Bcl6b KD**	**Etv5 KD**	**7**	Bcl6b KD- EDNRA- LMNB2- SGCB- SSPN- USP44- ZC4H2
**MArrest**	**Etv5 KD**	**5**	HOXB5- NEFM- PPM1J- PRKAR2A- SPTBN
**MArrest**	**Ing2 KO**	**3**	CCDC110- CYLC1- ITGA1
**Dazap1 Mutant**	**Oligospermia**	**3**	**ANKRD7**- **PLCZ1**- **TSSK2**
**Bcl6b KD**	**Pou3f1 KD**	**2**	GPR137B- LMNB2
**Etv5 KD**	**Teratospermia**	**2**	CXCR4- PAIP1
**Ikbkap KO**	**Oligospermia**	**2**	DMRTB1- OTX1
**MArrest**	**Pou3f1 KD**	**1**	BBS5
**Bcl6b KD**	**Oligospermia**	**1**	WIPI1
**Bcl6b KD**	**Teratospermia**	**1**	GPR137B
**Dazap1 Mutant**	**Ing2 KO**	**1**	HYAL6
**Dazap1 Mutant**	**Teratospermia**	**1**	GLIPR1L1
**Etv5 KD**	**Ikbkap KO**	**1**	MPPED2
**Ikbkap KO**	**Ing2 KO**	**1**	GM5622
**Pou3f1 KD**	**Teratospermia**	**1**	GPR137B

*Note*: **KO** = Knockout (mouse), **KD** = Knockdown (mouse); **MArrest** = Maturation Arrest(human); **Oligospermia** = (human); **Teratospermia** = (human).

### GSEA

**Figure 3:**
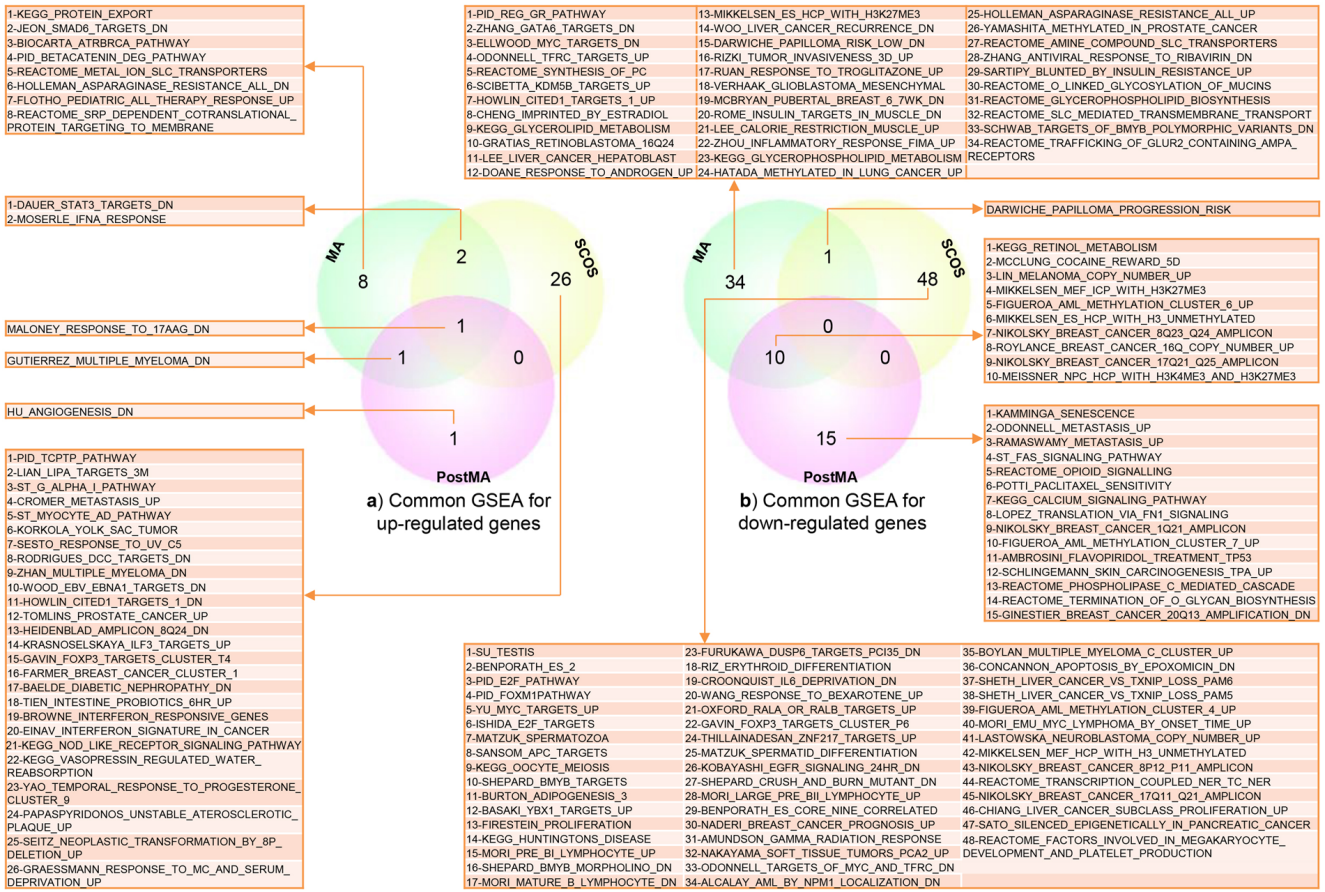
Gene set enrichment analysis (GSEA) of PostMA, MA and SCOS. (**a**) Common gene sets for up-regulated genes. (**b**) Common gene sets for down-regulated genes with 10 gene sets common between PostMA and MA.

We investigated gene sets based on all DEGs for MA, PostMA and SCOS (Fig. 3). In up-regulated gene sets, one gene set was common among PostMA, MA and SCOS, one gene set was common between PostMA and MA, and two gene sets were common between MA and SCOS. In the down-regulated gene sets, 10 gene sets were common between MA and PostMA, and one gene set was common between MA and SCOS. Common gene sets between each type of NOA are shown in Table 3.

**Table 3:** Common gene sets between DEGs of NOA types.

	Up-regulated gene sets	Down-regulated gene sets
**Common gene sets between MA, PMA and SCOS**	MALONEY_RESPONSE_TO_17AAG_DN	—
**Common gene sets between MA and PMA**	GUTIERREZ_MULTIPLE_MYELOMA_DN	ROYLANCE_BREAST_CANCER_16Q_COPY_NUMBER_UP
		NIKOLSKY_BREAST_CANCER_17Q21_Q25_AMPLICON
		FIGUEROA_AML_METHYLATION_CLUSTER_6_UP
		MIKKELSEN_MEF_ICP_WITH_H3K27ME3
		MEISSNER_NPC_HCP_WITH_H3K4ME3_AND_H3K27ME3
		KEGG_RETINOL_METABOLISM
		LIN_MELANOMA_COPY_NUMBER_UP
		NIKOLSKY_BREAST_CANCER_8Q23_Q24_AMPLICON
		MIKKELSEN_ES_HCP_WITH_H3_UNMETHYLATED
		MCCLUNG_COCAINE_REWARD_5D
**Common gene sets between MA and SCOS**	DAUER_STAT3_TARGETS_DN	DARWICHE_PAPILLOMA_PROGRESSION_RISK
	MOSERLE_IFNA_RESPONSE	

***Note***: MA = meiotic arrest; PMA = post meiotic arrest; SCOS = sertoly cell only syndrom.; DN = down.

### Principal component analysis (PCA)

**Figure 4:**
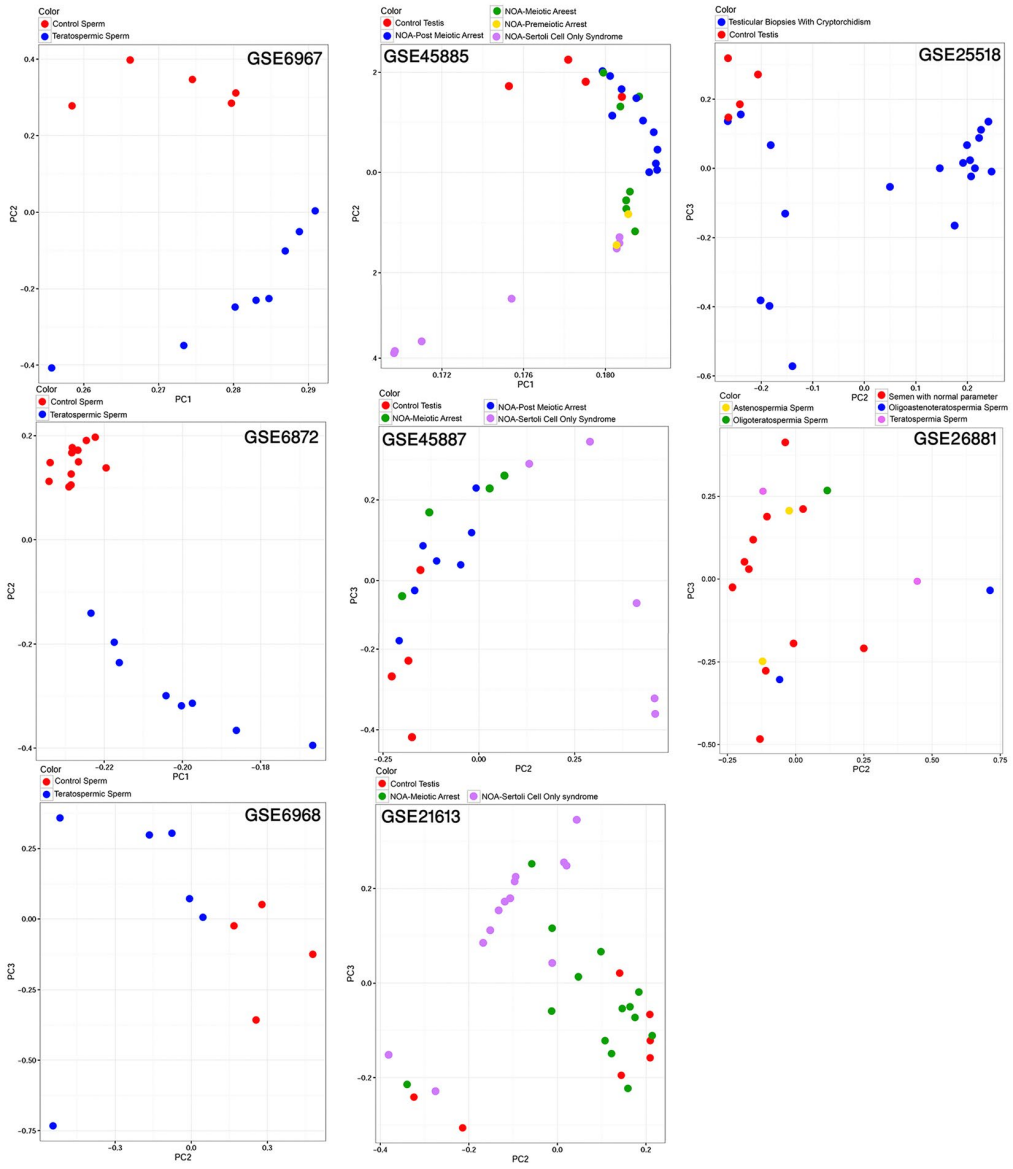
Principal component analysis (PCA) of eight human microarray libraries that show the visualization of quality, similarity and overlapping of library samples.

We found that in three teratospermia libraries, infertile samples were completely distinguished from normal samples. In three libraries from the stages of before and after meiotic arrest and SCOS, the clusters of normal samples and SCOS samples were separated, but there was an overlap between some samples of PostMA, MA and control cases (Fig. 4).

## Discussion

Understanding similarities among male infertility diseases could facilitate disease classification, help reveal hidden etiologies, and pave the way for new diagnostic tests and drugs. Toward this goal, we showed that *in silico* analyses are in good agreement with previous experimental results. Several studies have shown a direct association of an increase in steroid levels with azoospermia and oligospermia^19^, and male hormonal contraceptive trials use steroids to induce azoospermia and oligospermia^27,28^. Furthermore, steroid sex hormones regulate the spermatogenesis process and the development of skeletal muscles^29^. In this study, we observed that one of the major common BPs of up-regulated genes in MArrest and oligospermia was the development of muscle tissue and its regulation, and half of the common BPs of up-regulated genes in PostMA and MA were related to the steroid process. The *miR-145* regulates the development of smooth muscles^30^, and its high level of expression leads to the inhibition of cell-cell adhesion and cell motility^31^. We observed the overexpression of *miR-145* in NOA, and there were also up-regulated BPs related to muscle development in MArrest and oligospermia and down-regulated BPs related to sperm-egg recognition and sperm motility in NOA and teratospermia. Fu *et al*. found that several BPs of spermatogenesis-related genes were involved in sperm-egg recognition and fusion, and a protein-protein interaction analysis showed that these genes were down-regulated genes in teratospermia^32^. Moreover, a study on teratospermia suggested that the binding capacity of sperm to oocytes is low because of a lower expression of adhesion molecules in teratospermic spermatozoa^33^. In our study, we showed that half of the common down-regulated BPs in MArrest and teratospermia were related to sperm-egg adhesion.

Male germ cells are extremely sensitive to stress^34^. Glutathione is an important intracellular antioxidant, and several studies have indicated that decreased glutathione and glutathione transferase null genotypes lead to oligospermia and azoospermia^34–37^. We observed an up-regulation in glutathione transferase genes, which reduces the glutathione level in oligospermia and NOA (PostMA, MA and SCOS). Moreover, there are various results confirming the direct association between abnormal spermatogenesis due to the response to a stimulus^38–40^ and immune response^41^. We observed that one of the major similarities in MArrest, oligospermia and teratospermia was indeed overexpression of immune response, stimulus response and their regulation related genes. Bansal *et al*. revealed that idiopathic male infertility and asthenospermia are associated with changes in the expression of BPs, such as response to a stimulus, the immune system process, reproduction and the multicellular organismal process^24^. In this study, we showed the same BPs in MArrest, oligospermia and teratospermia. Noveski *et al*. determined that *miR-23b*, *miR-32*, *miR-154* and *miR-99* in MArrest and SCOS were up-regulated^42^, and we found that these genes were also up-regulated in NOA. *SOX9* is an essential protein for the maturation of sertoli cells and normal spermatogenesis, and it is a possible target of *miR-145* ^43,44^. Furthermore, we observed that *mir-145* is one of the common up-regulated genes. Approximately half of the common up-regulated genes in PostMA, MA and SCOS were miRNAs.

In 17 common down-regulated genes among PostMA, MA and SCOS, 9 genes were involved in spermatogenesis (*ADAM29* ^45^ *, DNAH17* ^46^ *, FAM166A* ^47^ *, FAM71F1* ^22^ *, GAPDHS* ^48^ *, GGN* ^22^ *, LYZL6* ^49^ *, SMCP* ^50^ and *SPACA4* ^22^), two genes were non-coding, and six genes (*ABHD1, ARMC12, FAM205A, PRR30, TEX38* and *TPPP2*) did not have specific and direct roles in spermatogenesis. *GGN* has a high level of expression in the late pachytene stage and primary spermatogenesis^51^. *ABHD1* is a member of the ABHD family, which has a role in spermatogenesis^52^. *TPPP2* has a high expression level in testes and has a role in testicular cancer^53^. In addition, in down-regulated genes common among PostMA, MA and SCOS, there were eight BPs that were classified into three clusters, including development and differentiation of spermatogenesis, sperm motility and sperm-egg recognition. Three common down-regulated CCs among PostMA, MA and SCOS and four CCs between MA and SCOS were related to the flagellum, which matches observations made by Fu *et al*.^32^.

Okada *et al*., Zhuang *et al*., Fu *et al*. and *Noveski et al*. identified meaningful BPs by using separate enrichment analyses for up- and down-regulated genes^19,32,42,54^.

In human and mouse male infertility, we observed the highest similarity between Etv5 and Pou3f1 KD in the up-regulated gene lists. Furthermore, in the down-regulated gene lists, we observed the highest similarity between MArrest and oligospermia among other types of male infertility, such as teratospermia and infertile mouse mutants. *DAZ* is one of the most important genes, and its deletion leads to NOA^8^. *Dazap1* is one of the isoforms of *DAZ*, and we observed the highest number of common genes between *Dazap1* mouse mutants and MArrest cases, with 21 common down-regulated genes, in comparison to other infertile mice. IKAP protein is encoded by the *Ikbkap* gene, which is a subunit of the Elongator complex and plays a role in chromatin remodeling^55^. Lin *et al*. revealed that a loss of function of *Ikbkap* in mice was the cause of defects in synapsis and meiotic recombination, leading to apoptosis and spermatogenesis arrest. In the present study, we observed that *Ikbkap* KO mice were highly similar to MArrest cases, with eight common down-regulated genes in which the process of meiosis was disrupted (Table 1 and Table 2). Tanespimycin or 17-allylamino-17-demethoxygeldanamycin (17AAG) is an antitumor drug that works by inhibiting *HSP90* (heat shock protein 90)^56^. In GSEA analysis, the low level of expression of a gene list in MA, PostMA and SCOS is similar to a gene set in ovarian cancer cells when treated with tanespimycin. However, there were no significant expression changes in *HSP90* in MA, PostMA and SCOS, although there were 17 gene sets related to spermatogenesis and 32 gene sets related to cancer in several stages of spermatogenesis arrest (Fig. 3).

In conclusion, we revealed that when comparing MArrest and oligospermia, the genes associated with immune response processes, muscle tissue development, and glutathione transferase and regulatory genes were up-regulated, and the genes related to spermatogenesis were down-regulated. When comparing PostMA, MA and SCOS, we found several common DEGs. Ten up-regulated miRNAs were common among all three NOA types, and the expression of genes associated with the spermatogenesis process was down-regulated. The proteins of these down-regulated genes have a function in sperm motility and flagellum development. Further work is needed to investigate the epigenomics and proteomics of male infertility to complement gene expression studies. Our study indicates which pathways one should focus on in future studies.

## Methods

**Figure 5:**
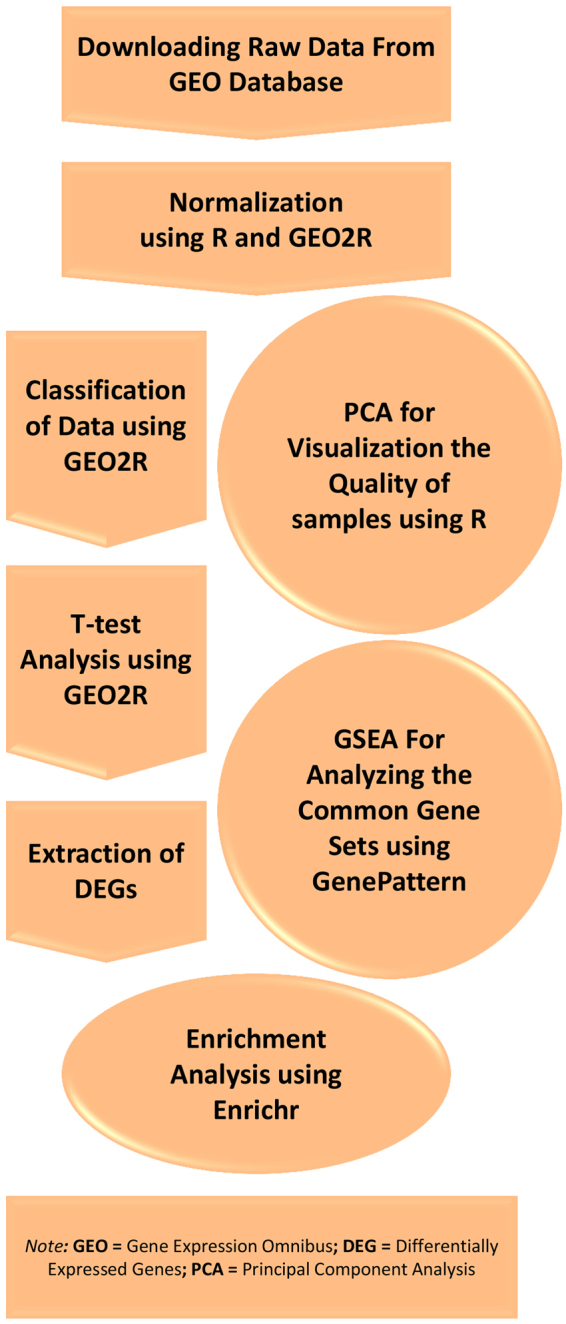
The workflow of this study.

In this study, we emphasized on unveiling underlying genes and signaling pathways and identifying putative biomarkers that are differentially expressed in male infertility microarray datasets. For this purpose, we used of functional enrichment analysis approaches including pathway enrichment analysis and GSEA. Figure 5 depicts the workflow used for this study.

### Microarray datasets and analysis

The microarray datasets related to male infertility were collected from the gene expression omnibus (GEO) repository^57^. Table 4 presents detailed information on the microarray datasets used. GEO2R (http://www.ncbi.nlm.nih.gov/geo/geo2r/), which employs a linear-based model for microarray analysis (limma), was used to obtain DEGs between male infertile and control samples. The top 100 DEGs were extracted in each infertile group against the normal group (*p*-value ≤ 0.01). Our expression study consists of three comparison steps, including (i) MArrest, oligospermia and teratospermia, (ii) PostMA, MA and SCOS, and (iii) nine types of human and mouse infertility.

**Table 4:** Information for the analyzed microarray libraries and infertility types^19–23,71–74^.

	Infertility Type	Tissue	Series ID	Platform ID	Number of Controls	Number of Cases	Platform Name
1	**Oligospermia & NOA**	Testise	**GSE14310**	GPL7870	3	23	Agilent-012097 Human 1 A Microarray (V2) G4110B
2	**NOA**	Testise	**GSE9210**	GPL887	11	47	Micro-CRIBI Human Oligo Array
3	**NOA**	Testise	**GSE45885**	GPL6244	4	27	Affymetrix Human Gene 1.0 ST Array
4	**NOA**	Testise	**GSE45887**	GPL6244	4	16	Affymetrix Human Gene 1.0 ST Array
5	**NOA**	Testise	**GSE21613**	GPL2891	4	17	GE Healthcare/Amersham Biosciences CodeLink™ UniSet Human 20 K I Bioarray
6	**NOA**	Testise	**GSE6023**	GPL2891	3	6	GE Healthcare/Amersham Biosciences CodeLink™ UniSet Human 20 K I Bioarray
7	**NOA**	Testise	**GSE45887**	GPL6244	12	5	GE Healthcare/Amersham Biosciences CodeLink™ UniSet Human 20 K I Bioarray
8	**OAT**	Sperm	**GSE26881**	GPL6244	11	7	Affymetrix Human Genome U133 Plus 2.0 Array
9	**Teratospermia**	Sperm	**GSE6872**	GPL570	13	8	Affymetrix Mouse Exon 1.0 ST Array
10	**Teratospermia**	Sperm	**GSE6967**	GPL2507	5	8	Sentrix Human-6 Expression BeadChip
11	**Teratospermia**	Sperm	**GSE6968**	GPL2700	4	6	Sentrix HumanRef-8 Expression BeadChip
12	**Mouse knockout (Ing2)**	Testise	**GSE18610**	GPL6246	3	5	Affymetrix Mouse Gene 1.0 ST Array
13	**Mouse knockout (Ikbkap)**	Testise	**GSE42230**	GPL6246	3	3	Affymetrix Mouse Gene 1.0 ST Array
14	**Mouse knockdown (Bcl6b-Etv5-Pou3f1)**	Testise	**GSE30683**	GPL1261	4	12	Affymetrix Mouse Genome 430 2.0 Array
15	**Mutant Mouse (Dazap1)**	Testise	**GSE42601**	GPL1261	3	3	Affymetrix Mouse Genome 430 2.0 Array

NOA = Non Obstructive Azoospermia, OAT = Oligo-Asterno-Teratospermia.

### Pathway enrichment analysis

After extracting up- and down-regulated genes from each library, the enriched GO terms (BP, CC and MF) and KEGG pathways were determined. Up- and down-regulated genes were then separately submitted to the Enrichr tool^58^. The common enriched GO terms and KEGG pathways for each comparison between an infertile group and a control group [such as NOA (PostMA, MA and SCOS), oligospermia and teratospermia] were extracted (*p*-value ≤ 0.01). We applied Venn diagrams (http://bioinformatics.psb.ugent.be/webtools/Venn/) for GO and KEGG pathway terms between three kinds of male infertility, MArrest, oligospermia and teratospermia, and three types of NOA (PostMA, MA and SCOS).

There are two strategies for GO and pathway enrichment analysis of DEGs: the analysis of all DEGs together or the split analysis of up- and down-regulated genes separately^59–61^. In this study, we used the second strategy, as suggested by other recent works^62–68^. Hong *et al*. compared the two types of GO and pathway enrichment analysis strategies using gene expression profiles of microarray and RNA-Seq, and they indicated that the separate strategy is more powerful and accurate^59^. When all DEGs are integrated together, the results might differ from when up-regulated and down-regulated genes are analyzed separately. For example, if a pathway has a considerable number of up-regulated genes and few down-regulated genes, the complete number of differentially regulated genes in the pathway might lead to statistically non-significant results, while computing the enrichment of over-represented genes separately might highlight an implication of the pathway in the system under investigation^61^. Therefore, we used the separated strategy to interpret the results.

### GSEA

GSEA is a powerful analytical method for interpreting gene expression data. We used software from the Broad institute^69^. All curated gene sets (C2.all.v 5.0 curated) were downloaded from the Molecular Signatures Database (MSigDB) and used to select significant gene sets based on the measurement of expression data^69^. A false discovery rate (FDR) less than 0.25 and *p*-values less than 0.01 were considered significant.

### PCA

The quality of eight human microarray libraries was examined with PCA. PCA was applied to eight normalized and log-transformed libraries of human male infertility using the R package^70^. All samples of each library were placed in a specific two-dimensional scatter plot without selection or weighting.

## Electronic supplementary material

Supplementary Information 
