# Supplementary material for: Comprehensive functional enrichment analysis of male infertility
Source: Sci Rep. 2017 Nov 17;7:15778. doi: 10.1038/s41598-017-16005-0 (PMC5693951; doi:10.1038/s41598-017-16005-0)
Supplement: Supplementary file 1 — Supplementary Information [file 41598_2017_16005_MOESM1_ESM.pdf]

SUPPLEMENTARY TABLES

Comprehensive functional enrichment analysis of male infertility

Seyed Morteza Razavi[1], Marjan Sabbaghian[2*], Mahdi Jalili[3], Adeleh Divsalar[1], Olaf Wolkenhauer[4], Ali Salehzadeh-Yazdi[4*]

[1]Department of Cell and Molecular Biology, Faculty of Biological Sciences, Kharazmi University, Tehran, Iran
[2]Department of Andrology, Reproductive Biomedicine Research Center, Royan Institute for Reproductive Biomedicine, ACECR, Tehran, Iran.
[3]Hematology, Oncology and SCT Research Center, Tehran University of Medical Sciences, Tehran, Iran.
[4]Department of Systems Biology and Bioinformatics, University of Rostock, 18051 Rostock, Germany

Correspondences:
Ali Salehzadeh-Yazdi
University of Rostock
Department of Systems Biology and Bioinformatics
Rostock, Germany
ali.salehzadeh-yazdi@uni-rostock.de

Marjan Sabbaghian
Department of Andrology, Reproductive Biomedicine Research Center
Royan Institute for Reproductive Biomedicine, ACECR,
Tehran, Iran
marjan.sabbaghian@gmail.com

Supplementary Table S1. Differentially expressed genes of azoo-oligo-terato-spermia

Supplementary Table S2. Up- and Down- regulated Biological Process, Cellular Component, Molecular Function and KEGG Pathways in Maturation Arrest Azoospermia, Oligospermia and Teratospermia.

Supplementary Table S3. Differentially expressed genes of Post Meiotic Arrest, Meiotic Arrest, Pre Meiotic Arrest and Sertoly Cell Only Syndrome

Supplementary Table S4. Up- and Down- regulated Biological Process, Cellular Component, Molecular Function and KEGG Pathways in Post MA, MA, Pre MA and SCOS

Supplementary Table S1. Differentially expressed genes of azoo-oligo-terato-spermia

Up- Regulated Genes						Down- Regulated Genes					
Maturation Arrest			oligospermia	teratospermia		Maturation Arrrest			oligospermia	teratospermia	
AAMDC	HIGD1A	PLCZ1	A2M	A1CF	RAPGEF2	AADAC	FSCB	SPACA4	ABHD2	ABHD16B	RDH8
ABCA10	HIST1H2AC	PLDN	ABCB10	ABCC13	RAPSN	ABHD1	FSCN3	SPACA7	ACSBG2	ACADM	RIMBP3C
ABCA6	HIST1H2BH	PLEK2	ABI3BP	ABCC9	RBL1	ABI3BP	FTMT	SPAG6	ACTL7B	ACOT11	RNF20
ABCA8	HIST2H2AB	PLEKHG4	ABRA	ACKR4	RBMS3	ACADSB	GADD45GIP1	SPANXA1	ACTRT2	ADAM32	RPL10A
ABCA9	HIVEP2	PLPP3	ACYP2	ACSM2A	RELT	ACR	GALNTL5	SPANXB1	ALS2CR11	ADAP2	RPL12
ABHD2	HLA-DMB	PMP22	ANK3	ACVR1B	RNF144B	ACRBP	GAPDHS	SPANXC	ANKRD7	ALDH1A2	RPL13A
ABI3BP	HLA-DPA1	PNPLA8	APOBEC2	ADAM18	ROR1	ACRV1	GEMIN4	SPATA16	APH1B	ALDOA	RPL15
ACAT2	HLA-DPB1	PNRC2	ARHGAP1	ADAMTS3	RORA	ACSBG2	GFRA2	SPATA17	ARL4A	ALOX15B	RPL22
ACSBG2	HLA-DQA1	POMK	ATP7B	ADAMTS5	RPL37A	ACSL6	GGN	SPATA18	ARMC12	ALOX5AP	RPL3
ACSM3	HLA-DRA	PP12719	C10ORF71	ADH1B	RPL6	ACTG2	GGTLC2	SPATA19	ASXL1	AMFR	RPL35A
ACSS2	HMGB1	PRKAA2	C11ORF21	ADH4	RSAD2	ACTL7A	GIMAP5	SPATA20	ATP23	APP	RPL4
ACSS3	HMGCLL1	PRKACB	CACNA1S	AGBL4	RTP5	ACTL7B	GINS1	SPATA22	ATP5G1	APPBP2	RPL8
ACTL7A	HMGCR	PRKRIR	CCDC102A	AK5	RUNDC3B	ACTRT2	GK2	SPATA3	BRD7	AQP11	RPLP2
ACTL7B	HMGCS1	PROK1	CCK	AKR7A2	RUNX1T1	ADAD1	GKAP1	SPATA31A7	C11ORF71	AQP2	RPS11
ADAMTS12	HNF4G	PRPS2	CD48	ALPP	SCD5	ADAM2	GLP1R	SPATA31C1	C12ORF56	ARFGAP1	RPS15
ADAMTS5	HNRNPU	PSMA2	CHL1	AMY2B	SCGB1D1	ADAM29	GLT1D1	SPATA4	C15ORF48	ARHGAP19	RPS16
ADIRF	HOOK1	PSMA3	CLEC10A	ANKFN1	SCN11A	ADAM32	GLT6D1	SPATA6	C1R	ARHGAP4	RPS24
AK3L1	HRASLS	PSMC6	CLEC14A	APCDD1L	SEMA6D	ADAM5	GPR135	SPATA9	C22ORF23	ARID4B	RPS3
AKAP13	HS3ST1	PTK2	CPNE6	ARG2	SERF1A	ADAM7	GPR64	SPATS1	C7ORF31	ARMC2-AS1	RPS5
AKR1C3	HS3ST3B1	PTPRA	CSRP3	ASTN2	SH3TC1	AIPL1	GPRASP1	SPERT	CALR3	ARPC5L	RSL24D1
ALDH1A3	HSD11B1	PTPRD	CUX1	ATP8B3	SHOX	AKAP1	GPX5	SPIN4	CCDC54	ATP5O	RSPH14
ANAPC10	HSD17B11	PWAR6	CYLD	B3GAT2	SLC22A7	AKAP4	GRIK5	SPINK2	CCDC71L	AURKA	SEC31B
ANGPTL1	HSD17B3	RAB41	CYP2C18	B4GALT2	SLC26A3	ALS2CR11	GSG1	SPTBN2	CDKN3	BAG2	SELENOF
ANKRD20A9P	HSD17B6	RALGAPA1	CYP8B1	BACH2	SLC39A8	AMOTL2	GSTM1	ST6GALNAC2	CENPL	BCKDHB	SEMG1
ANKRD7	HSD3B1	RARRES2	DHRS7	BBS4	SMCO1	ANKEF1	GSTTP2	STAM	CENPU	BCL9	SEMG2
ANP32A	HSD3B2	RBMS3	DMPK	BCL11A	SMG6	ANKRD13B	GTSF1L	STAT4	CEP76	BEX3	SEPP1
ANP32B	HSPA4L	RBMY2EP	DNAH1	BCL7A	SNAPC1	ANKRD5	GUCA1A	STK31	CFAP45	BEX4	SEPTIN10
ANTXR1	HSPA8	RBP1	DNAJC19	BCORL1	SNTG1	ANKRD53	HAAO	STON1-GTF2A1L	CFAP70	BLOC1S5	SEPTIN12
ANXA1	HSPB3	RBX1	DTNA	BICD1	SOCS4	ANKRD55	HEATR9	SUN3	CFAP74	BTBD6	SEPTIN5
APOL3	IER2	RDX	EDN2	BNC2	SORBS1	ANKRD7	HEMGN	SUN5	CKS2	C20ORF197	SF3B6
ARHGAP12	IER3	RFPL4A	FABP4	BRWD1	SOX11	ANO1	HIPK4	SYK	CLGN	C4ORF19	SGSH
ARHGEF6	IFI44L	RHBDF2	FBXW11	BZW2	SOX5	APBB3	HIST1H2AA	SYTL3	CRISP2	CACNA1I	SIAH1
ARL4A	IFIT1	ROBO1	GAD1	C11ORF1	SQSTM1	APOBEC4	HLA-DPB1	TBATA	DAZ1	CAMSAP1	SLC15A4
ASAP2	IGF1	RPA4	GIMAP6	C11ORF74	SRGAP2	AQP5	HMGB4	TBC1D21	DDX20	CARD16	SLC25A43
ASB13	IGF2	RPL15	GPM6A	C14ORF37	SRGAP2C	ARMC12	HOOK1	TBC1D3G	DHRS9	CBARP	SLC35A1
ATG2A	IGFBP4	RPL21P28	HEBP2	C16ORF87	SSX2	ARMC3	HOXB5	TBL3	DMRTB1	CBLN1	SLC38A9
ATM	IGFBP5	RPL23P8	IL12A	C19ORF57	STOX2	ARRB1	HSFY1	TCAIM	DNAI1	CBX1	SLC6A2
ATP1B3	IGFBP6	RPL31	ITPKC	C2ORF15	STRN	ASB17	HYDIN	TDRD3	DPY19L2	CBX3	SMCP
ATP5L2	IGSF6	RPL6	KAT6B	C5AR2	SYCE1L	ASPHD2	IGSF10	TEKT3	DZIP1	CCDC65	SNORA70
ATP6V0E1P2	IL16	RPS3A	KCNJ1	C5ORF24	SYNGR1	ATP1A2	IL13	TESMIN	EGR4	CCNI	SNORD139
AZI2	IL1A	RPS6KA3	KIAA1524	C5ORF51	SYNJ1	ATP1A4	INPP1	TEX26	FAM71E1	CCT4	SNORD68
B2M	IL1RAP	RRS1	LINC01108	CA13	SYTL3	ATP8B3	IQCF5	TEX29	FKBP6	CD38	SORCS1
BCCIP	ING4	SAMD9L	LINC01136	CACNA1E	SYTL4	BANF2	IRGC	TEX38	FNDC11	CD48	SPINK2
BCORL1	INHA	SC5D	LMOD2	CADPS	TAF4B	BAP1	ISG20L2	THBS1	FSCB	CD74	SPPL2C
BET1	INHBA	SCARNA7	LOC145783	CCDC9	TAS2R9	BBS5	ITGA1	THEG	FUCA2	CDC42EP3	SRGN
BHMT2	IPO7	SCARNA9	LOH12CR2	CCL16	TBPL2	BCL2L14	ITGA9	THYN1	GCA	CDK3	SRP9
BPI	IRGC	SDCBP	LPL	CCNG1	TC2N	BPIFA3	IZUMO4	TIMD4	GGH	CDR2	STK38
C11ORF63	ITM2A	SDHD	MAIP1	CCR1	TCF15	C10ORF62	JAKMIP1	TIMP4	GK2	CETN3	STRN3
C12ORF56	JAM3	SEPP1	MDP1	CDO1	TDG	C11ORF21	KCTD19	TMC7	GKAP1	CFAP221	SYCE3
C14ORF105	KCNK17	SFR1	MGAT2	CELF4	TEAD3	C11ORF52	KDM3A	TMCO5A	GMCL1P1	CHRNA5	SYNGR1
C15ORF48	KCNK4	SFRP1	MLLT1	CHRM3	TEFM	C11ORF71	KIAA1324	TMEM144	GTF2A2	CLDN11	TAF7L
C1D	KCNQ1	SFRP4	MSLN	CLDN6	TEP1	C12ORF25	KIAA1683	TMEM190	GTSF1	CLGN	TCEAL4
C1ORF115	KCNS3	SGOL1	MTNR1A	CLDN9	TERF1	C12ORF54	KIF2B	TMEM31	HELLS	COX6A1	TCEB2
C1ORF21	KIAA0484	SH3PXD2A	MYH6	CNKSR2	TMEM241	C14ORF119	KIR2DS2	TMEM64	HIP1	COX7B	TGFBI
C1QA	KIAA1199	SIX3	MYLK2	CNOT9	TMEM74	C16ORF78	KLF3-AS1	TNC	HMGB3	CSAD	TIMD4
C1QB	KIF1C	SIX6	MYOT	COL11A1	TNS2	C16ORF82	KLHL10	TNFAIP6	HRASLS	CSNK2B	TLR1
C1R	KIR2DL4	SKA2	NAP1L2	COL4A3	TRMU	C17ORF105	KLK5	TNFAIP8L1	HSPA4L	CST11	TM6SF1
C1S	KLHL9	SLC10A5	NOL6	CREB1	TRPM3	C17ORF53	KLRB1	TNP1	INPP4A	CTSD	TMCO2
C22ORF23	KMT2C	SLC16A2	NRP2	CT83	TTC5	C17ORF64	KNG1	TOR1A	IRGC	CUBN	TMEM17
C3ORF35	KRR1	SLC16A9	NTS	CUX2	TWISTNB	C17ORF74	KRT23	TPPP2	ITCH	CUTC	TMEM43
C6	KRT23	SLC25A2	NUS1	CYP19A1	UGT2A1	C19ORF45	KSR1	TRIM2	JKAMP	CXCR4	TNFRSF14
C6ORF166	KRT6A	SLC25A51	PHKG1	CYP20A1	UPF1	C1ORF108	LACRT	TRIM36	KCNK4	CYP7B1	TNP2
C6ORF203	KRTAP19-8	SLC37A3	PLN	DCLK1	VPS4A	C1ORF111	LAMP3	TRIM42	KIF15	DDX52	TOMM7
C8ORF49	KYAT3	SLC38A1	PLOD2	DCN	WBP1L	C1ORF158	LATS2	TSACC	KLHL1	DENR	TRAM1
C9ORF78	LAG3	SLC40A1	PLPP2	DICER1	WDR82	C1ORF192	LCA5L	TSKS	KPNA2	DGCR6L	TSACC
CA2	LAMA4	SLFN5	PMP22	DLD	WIF1	C1ORF194	LECT2	TSNAXIP1	KRT23	DHRS13	TSLP
CALB2	LAMTOR3	SLIRP	POU2F3	DLEU7	WISP3	C1ORF94	LELP1	TSPAN1	LDHAL6B	DHX15	TTC25
CASK	LDHC	SLITRK4	PRF1	DNAJB6	XKR4	C20ORF10	LILRA5	TSPAN16	LDHC	DKC1	TUBB4B
CAV2	LECT2	SMAD2	PRMT2	DNMT3A	ZAK	C20ORF141	LINC00238	TSSK2	LIMS3-LOC440895	DNMT1	TXNDC9
CBFB	LEPREL1	SMEK3P	PRRG4	EDARADD	ZC3H13	C20ORF173	LINC00301	TSSK3	LINC00467	DYNLRB2	UQCRFS1
CBR3	LHCGR	SMN1	QDPR	ELOVL4	ZNF234	C20ORF195	LINC00467	TSSK6	LINC00479	DYRK1A	VAV2
CBR4	LIMD1-AS1	SMPD1	RAB5B	EMD	ZNF302	C20ORF85	LINC00521	TTBK2	LINC01016	EEF1A1	VIMP
CBWD2	LIMS3-LOC440895	SNCA	RFTN1	ERC1	ZNF408	C22ORF23	LINC00938	TTC25	LOC100134040	EEF2	WASF1
CCL2	LINC00328	SNHG1	RPS6KA3	F7	ZNF454	C2ORF57	LOC100132147	TTC29	LRAT	EFCAB5	WEE1
CCNG1	LINC00643	SNORD113-3	RPS9	FAM172A	ZNF552	C3ORF22	LOC102725072	TTLL2	LSM14B	EHBP1	WHSC1L1
CD14	LINC00670	SNORD113-4	SELENOO	FAM212A	ZNF595	C3ORF30	LOC136242	TTLL3	LYAR	EI24	WRN
CD1E	LIPH	SNORD114-2	SLC25A6	FAM98B	ZNF786	C4ORF35	LOC157740	TULP2	MAEL	EID1	ZCCHC10
CD53	LIPT1	SNORD114-26	SLCO2A1	FBXL22		C5ORF60	LOC440149	TXNDC15	MAGEB2	EML4	ZFAND5
CDC37L1	LITAF	SNORD114-3	SLF1	FBXO38		C6ORF10	LOC440896	TXNDC2	MAN1A1	ENOPH1	ZFP2
CDKN3	LOC100130428	SNORD114-6	SNX13	FGF12		C6ORF106	LOC645961	UBAP2	MAPK8	EPB41L4A-AS1	ZFP36
CDR2	LOC100131541	SNORD13P2	SOD2	FKBPL		C6ORF204	LOC727924	UBL4B	MBTD1	ETFDH	ZFP91
CDS2	LOC100131943	SNORD56B	SPARCL1	FLRT1		C7ORF34	LOC728450	UBQLN3	MRPL42	EXOSC3	ZMIZ1
CELA2B	LOC100132099	SNORD58A	SRPK3	FOXJ1		C7ORF62	LRRC74A	UBQLNL	MS4A5	FAM162A	ZNF106
CENPC	LOC100132686	SNORD73A	SSH2	FRG1BP		C8ORF74	LRRK1	UNQ9391	MS4A6E	FAM175B	ZPBP
CENPI	LOC100287869	SNORD75	SSH3	FRG1JP		C9ORF131	LY6G5B	USP38	MTRF1L	FAM188A	ZRANB2
CEP76	LOC283481	SNORD78	TERF1	FSIP2		C9ORF173	LYPLA1	VCX2	MYBL1	FAM83F	ZW10
CFAP45	LOC389834	SNX2	TGFB1	FUS		C9ORF9	LYRM2	VCX3A	NKX3-1	FKBP14	
CFH	LOC63920	SPACA9	THPO	GABRB1		CA2	LYZL1	VCX3B	NUP62	FLVCR2	
CFHR1	LOC731157	SPAG6	TMOD2	GABRB3		CABLES2	LYZL2	VPRBP	OIP5	FN1	
CFHR3	LRIF1	SPARCL1	TMPRSS2	GDPD1		CABS1	LYZL4	VRK3	OLAH	FOPNL	
CFI	LRRN3	SPATA22	TMPRSS4	GJC2		CABYR	LYZL6	VTRNA1-1	OTX1	FTL	
CHST11	LRRTM3	SPATA7	TPO	GJD2		CAMKK2	MAP1A	VWA5B1	PAAF1	GAGE6	
CHST7	LRWD1	SPDYE1	TRHDE	GLIDR		CAPN11	MARCH_11	WBSCR28	PCSK4	GBP5	
CILP	LSM14B	SPINK2	TRIM2	GLYATL1		CAPZA3	MBL1P	WDR38	PCYT2	GIMAP6	
CKS2	LSM5	SPTLC3	UBE2QL1	GNAO1		CATSPER1	MC4R	WFDC9	PDE1B	GLIPR1L1	
CLEC2B	LSP1	SSMEM1	USP38	GPC2		CATSPER4	METRN	WSCD1	PDK1	GMFB	
CLGN	LTV1	STAG1	WRAP53	GPLD1		CATSPERG	MFSD6L	YPEL1	PIP4K2B	GNG10	
CLMP	LUM	STEAP1B	ZNF426	GPM6B		CAV2	MGAT4C	ZBTB32	PKP2	GNPNAT1	
CLN5	LYSMD3	STRADB	ZNF560	GRK5		CCDC110	MGC13057	ZC2HC1B	PLA2R1	GOLGA8N	
CMYA5	MAD2L1	STX10	ZNF738	GRM3		CCDC114	MMEL1	ZDHHC19	PLCZ1	GPR137B	
CNOT7	MAGEB16	SYCP3	ADCY1	GYS2		CCDC116	MORN3	ZDHHC20	PMS1	GPR183	
CNTNAP2	MAGEB2	TAF1D	ARHGEF7	HAND1		CCDC126	MRO	ZFAND1	POM121L9P	GPRIN2	
COL12A1	MAGEB6	TAPT1-AS1	ASRGL1	HBS1L		CCDC136	MROH2B	ZMAT3	PPP1R3F	GTF2A1L	
COMMD6	MAGOHB	TAS2R10	C19ORF60	HECW2		CCDC159	MROH5	ZMYND10	PRSS50	H2BFWT	
COPS2	MALAT1	TAS2R14	C5	HIF3A		CCDC185	MROH7-TTC4	ZNF10	RAB39B	HECA	
COX16	MAML2	TAS2R19	C8ORF33	HNRNPA0		CCDC54	MS4A6E	ZNF141	RFPL3S	HEXB	
CPM	MAP1B	TAS2R20	CACNA2D3	HNRNPR		CCDC7	MTNR1A	ZNF222	RGS22	HLA-DPA1	
CREB5	MAPKAPK5-AS1	TAS2R3	CCDC138	HNRNPU		CCER1	MTR	ZNF474	RIMBP3	HRASLS	
CRHR1	MARS	TAS2R31	CIDEC	HS3ST3A1		CCIN	MUC1	ZNF560	ROCK1	HSD17B7	
CRISP2	MCM4	TAS2R45	CISH	HYMAI		CCT8L2	MYL12B	ZNF683	SERPINA5	HSPBAP1	
CRYAB	MEF2C	TAS2R50	CLCN7	IAPP		CDC14A	MYOZ2	ZNF814	SGK494	HSPH1	
CRYGS	MEGF8	TBC1D3F	CORO2B	IBA57		CDK5R2	NAT2	ZNRF4	SH3RF2	ICAM1	
CRYZ	METTL15	TBX2	CS	IDNK		CEL	NCAN	ZXDC	SHPK	IFI30	
CSRNP3	METTL21EP	TCAM1P	CTNNB1	IFT57		CENPU	NEFM		SLC25A2	IMMT	
CTNNA3	METTL7A	TCN2	CYC1	IL22RA2		CEP170	NME5		SLC25A51	IMPA1	
CTPS2	MGARP	TCP11	DCP1B	IPCEF1		CFAP74	NOX3		SMCP	INO80C	
CTSK	MGC24103	TDRD7	DDR1-AS1	IQSEC3		CHD5	NPPA		SNHG6	ISCA1	
CXCL12	MGC70870	TET2	ENDOU	ISLR2		CHRM4	NR4A2		SOX5	ITPKA	
CXYORF2	MIR107	TEX101	EPX	KALRN		CISH	NSUN4		SPACA9	JAK2	
CYP11A1	MIR128-1	TEX11	ESRRA	KANK4		CLGN	NT5C1B		SPATA22	JAML	
CYP17A1	MIR145	TEX30	FAAH	KCNN3		CLPB	NUAK1		SPEF2	KHDRBS1	
DAPL1	MIR154	TEX37	GPSM1	KCP		CMTM2	NUDT12		SPINK2	KIN	
DCDC5	MIR15A	THSD7A	GSTM1	KIAA0408		CNN1	NUMBL		STK17B	KLHDC2	
DCN	MIR186	TKTL1	GSTT1	KLHL34		CNNM1	NUP210P1		TAF1B	KLHL10	
DCUN1D5	MIR18A	TKTL2	HIRIP3	LACC1		CNTN2	NYD-SP21		TBC1D3F	KRT23	
DDEF1	MIR197	TMA7	HLA-F	LBX2		COL19A1	OAZ3		TBL2	LANCL1	
DDIAS	MIR199A2	TMEM106B	HLF	LCN1		COL4A1	ODF1		TCAM1P	LIMS3-LOC440895	
DDX4	MIR21	TMEM99	ITLN1	LDB3		COX6B2	ODF2		TCP11	LINC00921	
DICER1	MIR214	TMPPE	KCNC4	LEP		CPA5	ODF3		TDRD7	LOC401052	
DIRAS3	MIR218-1	TMTC3	LOC101928203	LIN54		CPNE2	ODF3L1		TEX30	LPXN	
DKFZP779L1	MIR23B	TMX1	LYPLA1	LOC100128175		CPTP	OLAH		TKTL2	LRBA	
DLEU2	MIR27B	TNCRNA	MAP6D1	LOC100505609		CRISP2	OR2H1		TMEM191A	LRRFIP2	
DLK1	MIR29C	TNFRSF14	METRN	LOC101927331		CRYAA	OTUD7B		TMEM99	LSS	
DMKN	MIR30E	TOP2B	MS4A7	LOC101928622		CSNK1G2	OXCT2		TP53I3	LTA4H	
DMRTB1	MIR31	TP53TG3	NAA10	LOC283440		CST8	PBXIP1		TP53TG3	LYRM5	
DNAJB11	MIR32	TPD52L3	PLP1	LPAR4		CT83	PCK1		TPD52L3	MALAT1	
DNAJC12	MIR503	TRAF3	PPP1R1A	LRRC4C		CXCL16	PCSK4		TSSK2	MAPK6	
DNM3OS	MIR509	TRAK1	PPP2R2C	LRRFIP1		CXORF27	PCYT2		UBE2N	MCM3	
DOCK10	MIR99A	TRMT11	RGS14	LRTM1		CXORF61	PDCL2		WIPI1	METAP2	
DPM1	MIR99AHG	TRNT1	RNMT	LSP1		CXORF65	PDYN		ZFP64	METTL25	
DPPA3P2	MIRLET7A2	TTC16	RTN2	LYRM5		CYLC1	PDZD8		ZMYND12	MGP	
DRAM2	MIRLET7C	TUBB2A	S100A12	MCTP2		CYLC2	PFKP		ZNF233	MIF	
DSEL	MIRLET7F1	TUBB2B	S100B	MEGF10		CYP2B7P	PGK2		ZNF559	MIR6805	
DTNA	MIRLET7G	UBA6	SERPINI1	MFHAS1		DAW1	PHKG2			MPL	
EDN1	MLC1	UBE2N	SLC12A4	MIER3		DBT	PHOSPHO1			MRPL42	
EIF1AY	MMP26	UBLCP1	SLC3A2	MIGA1		DCST1	PILRB			MRPL43	
ELAVL3	MND1	UIMC1	SRGAP2C	MIR181A2HG		DCST2	PKIB			MRPS15	
EMX2	MORF4L1	UQCRB	STOML1	MIR9-2		DDI1	PLA2G6			MRPS2	
ENPP2	MPLKIP	USP51	TCL1A	MLC1		DDX4	PLCD4			MRPS33	
ENTPD1	MR1	USP9Y	TMEM151B	MLLT3		DEFB126	PLCZ1			MSMB	
ENY2	MRPL42	UTP11L	TMEM59	MOB3B		DKKL1	PMF1			MT1E	
EPB41L3	MRPL47	VAMP4	TRIM63	MOB3C		DMPK	POLE			MTERF1	
EPB41L4A	MS4A5	VAMP7	ZDHHC3	MOGAT1		DMRTB1	POM121L2			MTHFD2	
ERAP2	MS4A6A	VCX	ZNF317	MRC2		DNAH1	PPM1J			MTMR6	
ESCO2	MSMB	VCY		MRPL45		DNAH17	PPP1R32			NACA	
ETFDH	MSMO1	VNN2		MSH3		DNAI2	PPP3R2			NARS	
EXOC6	MT1E	VSX1		MYOZ3		DNAJC5B	PRDM1			NBAS	
EXPH5	MT1G	VTN		NAA16		DOC2A	PRKAR2A			NCKAP1L	
FAM103A1	MT1X	WDR75		NANOG		DOT1L	PRKCZ			NDUFA13	
FAM198B	MTMR1	WISP2		NASP		DPEP3	PRM1			NDUFAB1	
FAM3D	MYBL1	WNT11		NDP		DPH4	PRM2			NDUFB5	
FAM69A	MYCL1	XIST		NDST4		DRC7	PROCA1			NDUFC2	
FAT1	MYO1G	ZEB2		NEDD4L		DUS1L	PROX1			NEFH	
FCGR2B	NAA15	ZFAND1		NEK8		DUSP1	PRPH			NFXL1	
FKBP6	NAE1	ZFP36L2		NEMP1		DUSP13	PRR30			NSA2	
FLJ38894	NAMPT	ZNF141		NFATC2		DXO	PRSS54			NUDT4	
FLJ44451	NDRG2	ZNF189		NHLRC2		DYDC1	PTP4A1			NUP98	
FLRT2	NDUFS4	ZNF22		NIN		DYNLRB2	RASAL1			PAIP1	
FMOD	NKAPP1	ZNF233		NKAIN2		EAF1	RBMXL2			PAIP2	
FNBP1	NOLA1	ZNF260		NKTR		EDG5	RBX1			PATE1	
FOXG1	NOLC1	ZNF37A		NKX1-2		EFCAB14	REC8			PCGF5	
FPR1	NPLOC4	ZNF430		NKX3-1		EHD1	RGL2			PCM1	
FRG1	NPPB	ZNF486		NLRP3		EIF4E	RIMBP3			PCYT2	
FUSIP1	NR2F1	ZNF502		NNT		EIF4E2	RIMBP3C			PDCD10	
FXC1	NR2F2	ZNF503		NPAP1		EPHA6	RNASE11			PDHA2	
FZD4	NRDC	ZNF524		NPIPB5		ETAA1	RND2			PDK4	
GAK	NRK	ZNF525		NRSN1		FAM154A	RNF151			PEX19	
GALNT18	NSG1	ZNF627		NTRK3		FAM163A	ROPN1B			PHLDA2	
GAS5	NUP62	ZNF641		NUF2		FAM166A	ROPN1L			PIP	
GBP1	NUTM1	ZNF649		NUMBL		FAM183B	RPGRIP1			PJA2	
GDAP1	OCLM	ZNF682		NUP50		FAM185A	RWDD2A			PLRG1	
GFI1	OGN	ZNF737		NUP58		FAM186B	SAMD4A			PNPLA8	
GFRA2	OIP5	ZNF770		OLAH		FAM187B	SAXO1			PPDPF	
GGA2	OPCML	ZNF845		ONECUT2		FAM205A	SCD5			PPEF1	
GKAP1	OR10D3	ZSCAN2		OR12D2		FAM205B	SCP2D1			PPIB	
GLO1	OR10G8			PAQR6		FAM230C	SEC61A2			PPIL4	
GNG11	OR52H1			PBOV1		FAM60A	SEPTIN12			PPIL6	
GNPDA2	OR8G2			PCBP4		FAM71A	SH3GL3			PPM1D	
GPNMB	OSR2			PCDH9		FAM71B	SHCBP1L			PPP1R8	
GPR21	OSTC			PDE1A		FAM71C	SIRPD			PPP2CA	
GPR52	OXA1L			PDGFD		FAM71D	SLC16A7			PPP2R3A	
GPR75	P3H3			PEBP4		FAM71E2	SLC25A3			PRKAR1A	
GREB1	PAPSS2			PHKB		FAM71F1	SLC25A51			PRPS2	
GRIK2	PARP14			PHLDB2		FAM81B	SLC26A8			PRR14	
GRK5	PARP4			PNISR		FANCD2OS	SLC36A3			PSD3	
GSTA1	PCDH18			POLK		FBXO24	SLC4A11			PSMA5	
GSTA3	PCDHB18			POU2F3		FBXW10	SLC7A7			PSMA8	
GSTTP1	PCSK4			PPIL2		FEM1B	SLCO6A1			PSMB7	
GTF2A2	PDE1A			PPM1B		FER1L5	SLFNL1			PSMG1	
GTPBP10	PDE4D			PPP4R3A		FERMT1	SMCP			RAB28	
GYPB	PDE5A			PTER		FHL5	SNAP23			RACK1	
H19	PDE8B			PUS10		FLJ21408	SNX13			RAG1	
HAT1	PDGFRB			RAB18		FLJ40244	SOX30			RAPGEF6	
HEPH	PERP			RAB30		FNDC8	SOX5			RARB	
HERC4	PHF14			RAB40A		FOS	SPA17			RASA1	
HEXA-AS1	PIK3CA			RALGAPA2		FOXM1	SPACA3			RBM45	

Supplementary Table S2. Up- and Down- regulated Biological Process, Cellular Component, Molecular Function and KEGG Pathways in Maturation Arrest Azoospermia, Oligospermia and Teratospermia.

Biological Process of Up-regulated Genes in Marrest

	Term	GO ID	Count
1	carbohydrate derivative biosynthetic process	GO:1901137	11
2	cellular response to hormone stimulus	GO:0032870	10
3	lipid biosynthetic process	GO:0008610	10
4	response to steroid hormone	GO:0048545	10
5	activation of immune response	GO:0002253	9
6	aminoglycan metabolic process	GO:0006022	9
7	cellular response to lipid	GO:0071396	9
8	cellular response to organic cyclic compound	GO:0071407	9
9	glycosaminoglycan metabolic process	GO:0030203	9
10	organic hydroxy compound metabolic process	GO:1901615	9
11	regulation of cell growth	GO:0001558	9
12	response to inorganic substance	GO:0010035	9
13	response to radiation	GO:0009314	9
14	aminoglycan biosynthetic process	GO:0006023	8
15	cellular response to cytokine stimulus	GO:0071345	8
16	glycosaminoglycan biosynthetic process	GO:0006024	8
17	negative regulation of cell cycle	GO:0045786	8
18	organ morphogenesis	GO:0009887	8
19	positive regulation of secretion	GO:0051047	8
20	regulation of cell activation	GO:0050865	8
21	regulation of cell cycle process	GO:0010564	8
22	regulation of hormone levels	GO:0010817	8
23	regulation of leukocyte activation	GO:0002694	8
24	regulation of lymphocyte activation	GO:0051249	8
25	regulation of mitotic cell cycle	GO:0007346	8
26	response to alcohol	GO:0097305	8
27	skeletal system development	GO:0001501	8
28	small molecule biosynthetic process	GO:0044283	8
29	sulfur compound biosynthetic process	GO:0044272	8
30	sulfur compound metabolic process	GO:0006790	8
31	cellular hormone metabolic process	GO:0034754	7
32	cellular response to nitrogen compound	GO:1901699	7
33	cellular response to organonitrogen compound	GO:0071417	7
34	complement activation	GO:0006956	7
35	detection of chemical stimulus	GO:0009593	7
36	detection of chemical stimulus involved in sensory perception	GO:0050907	7
37	gamete generation	GO:0007276	7
38	hormone metabolic process	GO:0042445	7
39	humoral immune response	GO:0006959	7
40	immune response-activating signal transduction	GO:0002757	7
41	mitotic cell cycle	GO:0000278	7
42	multicellular organismal development	GO:0007275	7
43	multicellular organismal reproductive process	GO:0048609	7
44	negative regulation of growth	GO:0045926	7
45	negative regulation of phosphorylation	GO:0042326	7
46	negative regulation of transport	GO:0051051	7
47	nuclear division	GO:0000280	7
48	organelle fission	GO:0048285	7
49	organic hydroxy compound biosynthetic process	GO:1901617	7
50	protein activation cascade	GO:0072376	7
51	regulation of cell cycle phase transition	GO:1901987	7
52	regulation of mitotic cell cycle phase transition	GO:1901990	7
53	reproductive structure development	GO:0048608	7
54	response to drug	GO:0042493	7
55	response to metal ion	GO:0010038	7
56	steroid biosynthetic process	GO:0006694	7
57	steroid metabolic process	GO:0008202	7
58	alcohol biosynthetic process	GO:0046165	6
59	androgen biosynthetic process	GO:0006702	6
60	androgen metabolic process	GO:0008209	6
61	cellular component disassembly	GO:0022411	6
62	cellular response to inorganic substance	GO:0071241	6
63	cellular response to interferon-gamma	GO:0071346	6
64	cellular response to metal ion	GO:0071248	6
65	chromosome segregation	GO:0007059	6
66	cytokine-mediated signaling pathway	GO:0019221	6
67	extracellular matrix organization	GO:0030198	6
68	extracellular structure organization	GO:0043062	6
69	fatty acid metabolic process	GO:0006631	6
70	gonad development	GO:0008406	6
71	hormone biosynthetic process	GO:0042446	6
72	male gamete generation	GO:0048232	6
73	mitotic nuclear division	GO:0007067	6
74	modification-dependent macromolecule catabolic process	GO:0043632	6
75	modification-dependent protein catabolic process	GO:0019941	6
76	monocarboxylic acid metabolic process	GO:0032787	6
77	mRNA processing	GO:0006397	6
78	negative regulation of cell cycle phase transition	GO:1901988	6
79	negative regulation of cell cycle process	GO:0010948	6
80	negative regulation of immune system process	GO:0002683	6
81	negative regulation of mitotic cell cycle	GO:0045930	6
82	negative regulation of mitotic cell cycle phase transition	GO:1901991	6
83	positive regulation of kinase activity	GO:0033674	6
84	positive regulation of protein kinase activity	GO:0045860	6
85	positive regulation of secretion by cell	GO:1903532	6
86	proteolysis involved in cellular protein catabolic process	GO:0051603	6
87	regulation of immune effector process	GO:0002697	6
88	regulation of response to wounding	GO:1903034	6
89	response to estrogen	GO:0043627	6
90	response to interferon-gamma	GO:0034341	6
91	response to molecule of bacterial origin	GO:0002237	6
92	response to transition metal nanoparticle	GO:1990267	6
93	spermatogenesis	GO:0007283	6
94	ubiquitin-dependent protein catabolic process	GO:0006511	6
95	antigen processing and presentation	GO:0019882	5
96	antigen processing and presentation of peptide antigen	GO:0048002	5
97	ATP catabolic process	GO:0006200	5
98	brain development	GO:0007420	5
99	carboxylic acid biosynthetic process	GO:0046394	5
100	cell cycle checkpoint	GO:0000075	5
101	cellular process involved in reproduction in multicellular organism	GO:0022412	5
102	cellular response to cadmium ion	GO:0071276	5
103	cellular response to steroid hormone stimulus	GO:0071383	5
104	chemotaxis	GO:0006935	5
105	cholesterol biosynthetic process	GO:0006695	5
106	cholesterol metabolic process	GO:0008203	5
107	complement activation, classical pathway	GO:0006958	5
108	detection of chemical stimulus involved in sensory perception of bitter taste	GO:0001580	5
109	detection of chemical stimulus involved in sensory perception of taste	GO:0050912	5
110	DNA integrity checkpoint	GO:0031570	5
111	fatty acid biosynthetic process	GO:0006633	5
112	glycerolipid metabolic process	GO:0046486	5
113	interferon-gamma-mediated signaling pathway	GO:0060333	5
114	male gonad development	GO:0008584	5
115	monocarboxylic acid biosynthetic process	GO:0072330	5
116	mRNA catabolic process	GO:0006402	5
117	mucopolysaccharide metabolic process	GO:1903510	5
118	negative regulation of cell activation	GO:0050866	5
119	negative regulation of leukocyte activation	GO:0002695	5
120	negative regulation of lymphocyte activation	GO:0051250	5
121	negative regulation of response to external stimulus	GO:0032102	5
122	negative regulation of secretion	GO:0051048	5
123	negative regulation of secretion by cell	GO:1903531	5
124	nuclear-transcribed mRNA catabolic process	GO:0000956	5
125	nucleoside monophosphate catabolic process	GO:0009125	5
126	organic acid biosynthetic process	GO:0016053	5
127	positive regulation of cell activation	GO:0050867	5
128	positive regulation of cell cycle	GO:0045787	5
129	positive regulation of cell cycle process	GO:0090068	5
130	positive regulation of cell development	GO:0010720	5
131	positive regulation of leukocyte activation	GO:0002696	5
132	positive regulation of lymphocyte activation	GO:0051251	5
133	positive regulation of MAPK cascade	GO:0043410	5
134	positive regulation of protein maturation	GO:1903319	5
135	positive regulation of protein processing	GO:0010954	5
136	positive regulation of proteolysis	GO:0045862	5
137	protein folding	GO:0006457	5
138	purine nucleoside monophosphate catabolic process	GO:0009128	5
139	purine ribonucleoside monophosphate catabolic process	GO:0009169	5
140	regulation of cellular response to stress	GO:0080135	5
141	regulation of epithelial cell proliferation	GO:0050678	5
142	regulation of inflammatory response	GO:0050727	5
143	regulation of lipid metabolic process	GO:0019216	5
144	regulation of peptidyl-tyrosine phosphorylation	GO:0050730	5
145	regulation of smooth muscle cell proliferation	GO:0048660	5
146	regulation of T cell activation	GO:0050863	5
147	response to cadmium ion	GO:0046686	5
148	response to estradiol	GO:0032355	5
149	response to lipopolysaccharide	GO:0032496	5
150	response to peptide hormone	GO:0043434	5
151	ribonucleoside monophosphate catabolic process	GO:0009158	5
152	RNA catabolic process	GO:0006401	5
[illegible]	[illegible]	[illegible]	[illegible]

Biological Proccess of Up-regulated Genes in Oligospermia

	Term	GO ID	Count
1	muscle system process	GO:0003012	8
2	synaptic transmission	GO:0007268	8
3	cation homeostasis	GO:0055080	7
4	metal ion homeostasis	GO:0055065	7
5	muscle contraction	GO:0006936	7
6	positive regulation of defense response	GO:0031349	7
7	regulation of system process	GO:0044057	7
8	cellular divalent inorganic cation homeostasis	GO:0072503	6
9	cellular metal ion homeostasis	GO:0006875	6
10	divalent inorganic cation homeostasis	GO:0072507	6
11	positive regulation of cell development	GO:0010720	6
12	regulation of membrane potential	GO:0042391	6
13	response to drug	GO:0042493	6
14	response to inorganic substance	GO:0010035	6
15	calcium ion homeostasis	GO:0055074	5
16	calcium ion transport	GO:0006816	5
17	cellular calcium ion homeostasis	GO:0006874	5
18	divalent inorganic cation transport	GO:0072511	5
19	divalent metal ion transport	GO:0070838	5
20	leukocyte migration	GO:0050900	5
21	regulation of actin filament-based process	GO:0032970	5
22	regulation of anatomical structure size	GO:0090066	5
23	regulation of blood circulation	GO:1903522	5
24	regulation of cell morphogenesis involved in differentiation	GO:0010769	5
25	regulation of heart contraction	GO:0008016	5
26	regulation of innate immune response	GO:0045088	5
27	regulation of metal ion transport	GO:0010959	5
28	response to metal ion	GO:0010038	5
29	striated muscle contraction	GO:0006941	5
30	xenobiotic metabolic process	GO:0006805	5
31	circulatory system process	GO:0003013	4
32	monocarboxylic acid biosynthetic process	GO:0072330	4
33	muscle tissue development	GO:0060537	4
34	negative regulation of intracellular transport	GO:0032387	4
35	neuron migration	GO:0001764	4
36	neuron projection morphogenesis	GO:0048812	4
37	positive regulation of cell morphogenesis involved in differentiation	GO:0010770	4
38	protein acylation	GO:0043543	4
39	protein dephosphorylation	GO:0006470	4
40	regulation of G-protein coupled receptor protein signaling pathway	GO:0008277	4
41	regulation of ion homeostasis	GO:2000021	4
42	regulation of muscle contraction	GO:0006937	4
43	regulation of muscle system process	GO:0090257	4
44	regulation of protein targeting	GO:1903533	4
45	regulation of the force of heart contraction	GO:0002026	4
46	response to wounding	GO:0009611	4
47	striated muscle tissue development	GO:0014706	4
48	calcium-mediated signaling	GO:0019722	3
49	lipid homeostasis	GO:0055088	3
50	memory	GO:0007613	3
51	modification of morphology or physiology of other organism	GO:0035821	3
52	modification of morphology or physiology of other organism involved in symbiotic interaction	GO:0051817	3
53	myeloid leukocyte activation	GO:0002274	3
54	peptidyl-tyrosine dephosphorylation	GO:0035335	3
55	regulation of blood vessel size	GO:0050880	3
56	regulation of heart rate	GO:0002027	3
57	regulation of sodium ion transport	GO:0002028	3
58	regulation of tube size	GO:0035150	3
59	skeletal muscle tissue development	GO:0007519	3
60	vascular process in circulatory system	GO:0003018	3
61	acylglycerol catabolic process	GO:0046464	2
62	adherens junction assembly	GO:0034333	2
63	cardiac muscle tissue development	GO:0048738	2
64	glutathione derivative biosynthetic process	GO:1901687	2
65	glutathione derivative metabolic process	GO:1901685	2
66	glycerolipid catabolic process	GO:0046503	2
67	hair cell differentiation	GO:0035315	2
68	hydrogen peroxide metabolic process	GO:0042743	2
69	long-term memory	GO:0007616	2
70	long-term synaptic potentiation	GO:0060291	2
71	mast cell activation	GO:0045576	2
72	membrane assembly	GO:0071709	2
73	modification by symbiont of host morphology or physiology	GO:0044003	2
74	modulation by organism of defense response of other organism involved in symbiotic interaction	GO:0052255	2
75	modulation by organism of immune response of other organism involved in symbiotic interaction	GO:0052552	2
76	modulation by symbiont of host cellular process	GO:0044068	2
77	modulation by symbiont of host defense response	GO:0052031	2
78	modulation by symbiont of host immune response	GO:0052553	2
79	modulation by virus of host morphology or physiology	GO:0019048	2
80	modulation by virus of host process	GO:0019054	2
81	muscle adaptation	GO:0043500	2
82	myofibril assembly	GO:0030239	2
83	negative regulation of calcium ion transport	GO:0051926	2
84	negative regulation of establishment of protein localization to plasma membrane	GO:0090005	2
85	negative regulation of NF-kappaB import into nucleus	GO:0042347	2
86	negative regulation of protein localization to plasma membrane	GO:1903077	2
87	negative regulation of transcription factor import into nucleus	GO:0042992	2
88	neuromuscular junction development	GO:0007528	2
89	neuromuscular synaptic transmission	GO:0007274	2
90	neutral lipid catabolic process	GO:0046461	2
91	N-terminal protein amino acid modification	GO:0031365	2
92	positive regulation by organism of defense response of other organism involved in symbiotic interaction	GO:0052510	2
93	positive regulation by symbiont of host defense response	GO:0052509	2
94	positive regulation of histone acetylation	GO:0035066	2
95	positive regulation of neural precursor cell proliferation	GO:2000179	2
96	positive regulation of peptidyl-lysine acetylation	GO:2000758	2
97	positive regulation of protein acetylation	GO:1901985	2
98	protein import into nucleus, translocation	GO:0000060	2
99	protein palmitoylation	GO:0018345	2
100	regulation of actin filament-based movement	GO:1903115	2
101	regulation of calcium ion import	GO:0090279	2
102	regulation of DNA biosynthetic process	GO:2000278	2
103	regulation of filopodium assembly	GO:0051489	2
104	regulation of glycoprotein biosynthetic process	GO:0010559	2
105	regulation of glycoprotein metabolic process	GO:1903018	2
106	regulation of histone acetylation	GO:0035065	2
107	regulation of interleukin-17 production	GO:0032660	2
108	regulation of lamellipodium assembly	GO:0010591	2
109	regulation of lamellipodium organization	GO:1902743	2
110	regulation of membrane depolarization	GO:0003254	2
111	regulation of mitochondrial membrane potential	GO:0051881	2
112	regulation of telomerase activity	GO:0051972	2
113	release of cytochrome c from mitochondria	GO:0001836	2
114	response to defenses of other organism involved in symbiotic interaction	GO:0052173	2
115	response to host	GO:0075136	2
116	response to host defenses	GO:0052200	2
117	response to host immune response	GO:0052572	2
118	response to immune response of other organism involved in symbiotic interaction	GO:0052564	2
119	response to lithium ion	GO:0010226	2
120	skeletal muscle adaptation	GO:0043501	2
121	striated muscle adaptation	GO:0014888	2
122	triglyceride catabolic process	GO:0019433	2

Biological Proccess of Up-regulated Genes in Teratospermia

	Term	GO ID	Count
1	negative regulation of transport	GO:0051051	7
2	regulation of neuron differentiation	GO:0045664	7
3	cellular response to nitrogen compound	GO:1901699	6
4	cellular response to organic cyclic compound	GO:0071407	6
5	negative regulation of cell development	GO:0010721	6
6	negative regulation of nervous system development	GO:0051961	6
7	negative regulation of neurogenesis	GO:0050768	6
8	positive regulation of cell development	GO:0010720	6
9	positive regulation of nervous system development	GO:0051962	6
10	positive regulation of neurogenesis	GO:0050769	6
11	negative regulation of response to external stimulus	GO:0032102	5
12	nervous system development	GO:0007399	5
13	positive regulation of cell migration	GO:0030335	5
14	positive regulation of cell motility	GO:2000147	5
15	positive regulation of cellular component movement	GO:0051272	5
16	positive regulation of locomotion	GO:0040017	5
17	regulation of neuron projection development	GO:0010975	5
18	sulfur compound metabolic process	GO:0006790	5
19	aminoglycan biosynthetic process	GO:0006023	4
20	aminoglycan metabolic process	GO:0006022	4
21	cell junction organization	GO:0034330	4
22	cell-cell junction organization	GO:0045216	4
23	cellular carbohydrate metabolic process	GO:0044262	4
24	DNA replication	GO:0006260	4
25	glycosaminoglycan biosynthetic process	GO:0006024	4
26	glycosaminoglycan metabolic process	GO:0030203	4
27	mucopolysaccharide metabolic process	GO:1903510	4
28	negative regulation of protein transport	GO:0051224	4
29	negative regulation of secretion	GO:0051048	4
30	negative regulation of secretion by cell	GO:1903531	4
31	positive regulation of neuron differentiation	GO:0045666	4
32	regulation of cysteine-type endopeptidase activity involved in apoptotic process	GO:0043281	4
33	regulation of protein secretion	GO:0050708	4
34	action potential	GO:0001508	3
35	activation of cysteine-type endopeptidase activity	GO:0097202	3
36	activation of cysteine-type endopeptidase activity involved in apoptotic process	GO:0006919	3
37	cytokine production	GO:0001816	3
38	embryonic heart tube morphogenesis	GO:0003143	3
39	epithelial cell development	GO:0002064	3
40	epithelial tube morphogenesis	GO:0060562	3
41	heart looping	GO:0001947	3
42	membrane depolarization	GO:0051899	3
43	mitotic cell cycle checkpoint	GO:0007093	3
44	negative regulation of gliogenesis	GO:0014014	3
45	negative regulation of protein secretion	GO:0050709	3
46	polyol metabolic process	GO:0019751	3
47	positive regulation of developmental growth	GO:0048639	3
48	regulation of dendrite development	GO:0050773	3
49	regulation of gliogenesis	GO:0014013	3
50	regulation of neuron migration	GO:2001222	3
51	response to cAMP	GO:0051591	3
52	response to organophosphorus	GO:0046683	3
53	telomere maintenance	GO:0000723	3
54	telomere organization	GO:0032200	3
55	tube morphogenesis	GO:0035239	3
56	zymogen activation	GO:0031638	3
57	2'-deoxyribonucleotide metabolic process	GO:0009394	2
58	activation of Ras GTPase activity	GO:0032856	2
59	acylglycerol homeostasis	GO:0055090	2
60	alditol metabolic process	GO:0019400	2
61	calcium-independent cell-cell adhesion via plasma membrane cell-adhesion molecules	GO:0016338	2
62	cardiac muscle cell action potential	GO:0086001	2
63	cardiac muscle cell action potential involved in contraction	GO:0086002	2
64	cellular response to cAMP	GO:0071320	2
65	cellular response to sterol	GO:0036315	2
66	cGMP metabolic process	GO:0046068	2
67	chemokine-mediated signaling pathway	GO:0070098	2
68	deoxyribonucleotide metabolic process	GO:0009262	2
69	deoxyribose phosphate metabolic process	GO:0019692	2
70	eating behavior	GO:0042755	2
71	glycerol metabolic process	GO:0006071	2
72	mismatch repair	GO:0006298	2
73	mRNA stabilization	GO:0048255	2
74	negative regulation of blood pressure	GO:0045776	2
75	negative regulation of cytokine secretion	GO:0050710	2
76	negative regulation of dendrite development	GO:2000171	2
77	negative regulation of glial cell proliferation	GO:0060253	2
78	negative regulation of ion transmembrane transporter activity	GO:0032413	2
79	negative regulation of NF-kappaB import into nucleus	GO:0042347	2
80	negative regulation of protein localization to cell surface	GO:2000009	2
81	negative regulation of systemic arterial blood pressure	GO:0003085	2
82	negative regulation of transcription factor import into nucleus	GO:0042992	2
83	N-terminal protein amino acid modification	GO:0031365	2
84	placenta development	GO:0001890	2
85	positive regulation of multicellular organism growth	GO:0040018	2
86	positive regulation of sodium ion transport	GO:0010765	2
87	quinone metabolic process	GO:1901661	2
88	regulation of dendritic spine development	GO:0060998	2
89	regulation of glial cell proliferation	GO:0060251	2
90	regulation of membrane depolarization	GO:0003254	2
91	regulation of NF-kappaB import into nucleus	GO:0042345	2
92	regulation of protein localization to cell surface	GO:2000008	2
93	regulation of sodium ion transmembrane transport	GO:1902305	2
94	regulation of sodium ion transmembrane transporter activity	GO:2000649	2
95	response to sterol	GO:0036314	2
96	RNA stabilization	GO:0043489	2
97	RNA-dependent DNA replication	GO:0006278	2
98	triglyceride homeostasis	GO:0070328	2

Biological Process of Down-regulated Genes in Marrest

	Term	GO ID	Count
1	multicellular organismal reproductive process	GO:0048609	19
2	gamete generation	GO:0007276	18
3	male gamete generation	GO:0048232	18
4	spermatogenesis	GO:0007283	18
5	multicellular organismal development	GO:0007275	13
6	cellular process involved in reproduction in multicellular organism	GO:0022412	12
7	germ cell development	GO:0007281	9
8	spermatid development	GO:0007286	9
9	regulation of homeostatic process	GO:0032844	8
10	positive regulation of homeostatic process	GO:0032846	7
11	sperm motility	GO:0030317	7
12	extracellular matrix organization	GO:0030198	6
13	extracellular structure organization	GO:0043062	6
14	fertilization	GO:0009566	6
15	microtubule-based movement	GO:0007018	6
16	microtubule-based process	GO:0007017	6
17	response to peptide	GO:1901652	6
18	response to peptide hormone	GO:0043434	6
19	small molecule biosynthetic process	GO:0044283	6
20	calcium ion homeostasis	GO:0055074	5
21	cellular calcium ion homeostasis	GO:0006874	5
22	cellular divalent inorganic cation homeostasis	GO:0072503	5
23	divalent inorganic cation homeostasis	GO:0072507	5
24	muscle system process	GO:0003012	5
25	negative regulation of intracellular signal transduction	GO:1902532	5
26	positive regulation of secretion	GO:0051047	5
27	regulation of membrane potential	GO:0042391	5
28	single-organism behavior	GO:0044708	5
29	cell recognition	GO:0008037	4
30	cell-cell recognition	GO:0009988	4
31	cilium movement	GO:0003341	4
32	cytosolic calcium ion homeostasis	GO:0051480	4
33	epithelial cilium movement	GO:0003351	4
34	kidney development	GO:0001822	4
35	microtubule cytoskeleton organization	GO:0000226	4
36	muscle contraction	GO:0006936	4
37	ncRNA processing	GO:0034470	4
38	negative regulation of neuron apoptotic process	GO:0043524	4
39	negative regulation of neuron death	GO:1901215	4
40	positive regulation of leukocyte differentiation	GO:1902107	4
41	regulation of cyclic nucleotide metabolic process	GO:0030799	4
42	regulation of ion homeostasis	GO:2000021	4
43	regulation of leukocyte differentiation	GO:1902105	4
44	regulation of neuron apoptotic process	GO:0043523	4
45	regulation of synaptic transmission	GO:0050804	4
46	response to alcohol	GO:0097305	4
47	second-messenger-mediated signaling	GO:0019932	4
48	single fertilization	GO:0007338	4
49	sperm-egg recognition	GO:0035036	4
50	adult behavior	GO:0030534	3
51	binding of sperm to zona pellucida	GO:0007339	3
52	calcium-mediated signaling	GO:0019722	3
53	carbohydrate catabolic process	GO:0016052	3
54	cell maturation	GO:0048469	3
55	cell wall macromolecule catabolic process	GO:0016998	3
56	cell wall macromolecule metabolic process	GO:0044036	3
57	cellular response to metal ion	GO:0071248	3
58	developmental maturation	GO:0021700	3
59	DNA conformation change	GO:0071103	3
60	DNA packaging	GO:0006323	3
61	glycolytic process	GO:0006096	3
62	granulocyte chemotaxis	GO:0071621	3
63	granulocyte migration	GO:0097530	3
64	hydrogen transport	GO:0006818	3
65	integrin-mediated signaling pathway	GO:0007229	3
66	leukocyte chemotaxis	GO:0030595	3
67	membrane depolarization	GO:0051899	3
68	multi-multicellular organism process	GO:0044706	3
69	multi-organism reproductive process	GO:0044703	3
70	myeloid leukocyte migration	GO:0097529	3
71	negative regulation of MAPK cascade	GO:0043409	3
72	neutrophil chemotaxis	GO:0030593	3
73	neutrophil migration	GO:1990266	3
74	peptidyl-tyrosine dephosphorylation	GO:0035335	3
75	positive regulation of bone remodeling	GO:0046852	3
76	positive regulation of bone resorption	GO:0045780	3
77	positive regulation of cyclic nucleotide biosynthetic process	GO:0030804	3
78	positive regulation of cyclic nucleotide metabolic process	GO:0030801	3
79	positive regulation of lymphocyte differentiation	GO:0045621	3
80	positive regulation of nucleotide biosynthetic process	GO:0030810	3
81	positive regulation of purine nucleotide biosynthetic process	GO:1900373	3
82	positive regulation of tissue remodeling	GO:0034105	3
83	protein modification by small protein removal	GO:0070646	3
84	proton transport	GO:0015992	3
85	pyruvate metabolic process	GO:0006090	3
86	regulation of blood vessel size	GO:0050880	3
87	regulation of bone remodeling	GO:0046850	3
88	regulation of bone resorption	GO:0045124	3
89	regulation of calcium ion transport into cytosol	GO:0010522	3
90	regulation of cyclic nucleotide biosynthetic process	GO:0030802	3
91	regulation of exocytosis	GO:0017157	3
92	regulation of signal transduction by p53 class mediator	GO:1901796	3
93	regulation of tissue remodeling	GO:0034103	3
94	regulation of tube size	GO:0035150	3
95	reproduction	GO:0000003	3
96	response to calcium ion	GO:0051592	3
97	response to ketone	GO:1901654	3
98	response to retinoic acid	GO:0032526	3
99	response to temperature stimulus	GO:0009266	3
100	rRNA metabolic process	GO:0016072	3
101	rRNA processing	GO:0006364	3
102	sensory perception of chemical stimulus	GO:0007606	3
103	single-organism carbohydrate catabolic process	GO:0044724	3
104	vascular process in circulatory system	GO:0003018	3
105	acrosome reaction	GO:0007340	2
106	ATP-dependent chromatin remodeling	GO:0043044	2
107	benzene-containing compound metabolic process	GO:0042537	2
108	bone morphogenesis	GO:0060349	2
109	branched-chain amino acid catabolic process	GO:0009083	2
110	branched-chain amino acid metabolic process	GO:0009081	2
111	calcium ion-dependent exocytosis	GO:0017156	2
112	cellular response to heat	GO:0034605	2
113	chromosome condensation	GO:0030261	2
114	cilium or flagellum-dependent cell motility	GO:0001539	2
115	DNA damage response, signal transduction by p53 class mediator resulting in transcription of p21 class mediator	GO:0006978	2
116	DNA damage response, signal transduction resulting in transcription	GO:0042772	2
117	endosome organization	GO:0007032	2
118	fusion of sperm to egg plasma membrane	GO:0007342	2
119	histone exchange	GO:0043486	2
120	ionotropic glutamate receptor signaling pathway	GO:0035235	2
121	multicellular organism reproduction	GO:0032504	2
122	NAD metabolic process	GO:0019674	2
123	negative regulation of epidermal growth factor receptor signaling pathway	GO:0042059	2
124	negative regulation of interleukin-8 production	GO:0032717	2
125	negative regulation of steroid metabolic process	GO:0045939	2
126	nucleosome disassembly	GO:0006337	2
127	one-carbon metabolic process	GO:0006730	2
128	pancreas development	GO:0031016	2
129	phospholipid transport	GO:0015914	2
130	plasma membrane fusion	GO:0045026	2
131	positive regulation of alpha-beta T cell differentiation	GO:0046638	2
132	positive regulation of B cell differentiation	GO:0045579	2
[illegible]	[illegible]	[illegible]	[illegible]

Biological Proccess of Down-regulated Genes in Oligospermia

	Term	GO ID	Count
1	multicellular organismal development	GO:0007275	11
2	multicellular organismal reproductive process	GO:0048609	10
3	gamete generation	GO:0007276	9
4	male gamete generation	GO:0048232	8
5	spermatogenesis	GO:0007283	8
6	cellular process involved in reproduction in multicellular organism	GO:0022412	6
7	chromatin modification	GO:0016568	6
8	nuclear division	GO:0000280	5
9	organelle fission	GO:0048285	5
10	fertilization	GO:0009566	4
11	germ cell development	GO:0007281	4
12	protein folding	GO:0006457	4
13	chromatin assembly or disassembly	GO:0006333	3
14	chromatin remodeling	GO:0006338	3
15	chromatin remodeling at centromere	GO:0031055	3
16	DNA alkylation	GO:0006305	3
17	DNA methylation	GO:0006306	3
18	DNA methylation or demethylation	GO:0044728	3
19	DNA modification	GO:0006304	3
20	gene silencing	GO:0016458	3
21	meiotic cell cycle process	GO:1903046	3
22	meiotic nuclear division	GO:0007126	3
23	polyol metabolic process	GO:0019751	3
24	protein-DNA complex assembly	GO:0065004	3
25	regulation of gene expression, epigenetic	GO:0040029	3
26	spermatid development	GO:0007286	3
27	ATP biosynthetic process	GO:0006754	2
28	CENP-A containing nucleosome assembly	GO:0034080	2
29	chromosome organization involved in meiosis	GO:0070192	2
30	cytoplasmic pattern recognition receptor signaling pathway	GO:0002753	2
31	DNA methylation involved in gamete generation	GO:0043046	2
32	DNA replication-independent nucleosome assembly	GO:0006336	2
33	DNA replication-independent nucleosome organization	GO:0034724	2
34	gene silencing by RNA	GO:0031047	2
35	histone exchange	GO:0043486	2
36	nucleotide-binding domain, leucine rich repeat containing receptor signaling pathway	GO:0035872	2
37	piRNA metabolic process	GO:0034587	2
38	primary alcohol metabolic process	GO:0034308	2
39	protein K63-linked ubiquitination	GO:0070534	2
40	retinoic acid metabolic process	GO:0042573	2
41	retinol metabolic process	GO:0042572	2
42	sperm motility	GO:0030317	2
43	synapsis	GO:0007129	2

Biological Proccess of Down-regulated Genes in eratospermia

	Term	GO ID	Count
1	gene expression	GO:0010467	24
2	translation	GO:0006412	21
3	protein localization to organelle	GO:0033365	18
4	translational elongation	GO:0006414	18
5	establishment of protein localization to organelle	GO:0072594	17
6	mRNA catabolic process	GO:0006402	17
7	nuclear-transcribed mRNA catabolic process	GO:0000956	17
8	protein targeting	GO:0006605	17
9	RNA catabolic process	GO:0006401	17
10	single-organism cellular localization	GO:1902580	17
11	cellular component disassembly	GO:0022411	16
12	cotranslational protein targeting to membrane	GO:0006613	16
13	establishment of protein localization to endoplasmic reticulum	GO:0072599	16
14	establishment of protein localization to membrane	GO:0090150	16
15	nuclear-transcribed mRNA catabolic process, nonsense-mediated decay	GO:0000184	16
16	protein localization to endoplasmic reticulum	GO:0070972	16
17	protein localization to membrane	GO:0072657	16
18	protein targeting to ER	GO:0045047	16
19	protein targeting to membrane	GO:0006612	16
20	SRP-dependent cotranslational protein targeting to membrane	GO:0006614	16
21	translational initiation	GO:0006413	16
22	cellular protein complex disassembly	GO:0043624	15
23	macromolecular complex disassembly	GO:0032984	15
24	protein complex disassembly	GO:0043241	15
25	translational termination	GO:0006415	15
26	viral life cycle	GO:0019058	15
27	viral transcription	GO:0019083	15
28	cellular response to cytokine stimulus	GO:0071345	9
29	lipid biosynthetic process	GO:0008610	9
30	regulation of cytokine production	GO:0001817	9
31	cytokine-mediated signaling pathway	GO:0019221	8
32	generation of precursor metabolites and energy	GO:0006091	8
33	protein folding	GO:0006457	8
34	chromatin modification	GO:0016568	7
35	positive regulation of GTP catabolic process	GO:0033126	7
36	positive regulation of GTPase activity	GO:0043547	7
37	positive regulation of kinase activity	GO:0033674	7
38	posttranscriptional regulation of gene expression	GO:0010608	7
39	regulation of translation	GO:0006417	7
40	alcohol metabolic process	GO:0006066	6
41	covalent chromatin modification	GO:0016569	6
42	histone modification	GO:0016570	6
43	regulation of Ras GTPase activity	GO:0032318	6
44	antigen processing and presentation	GO:0019882	5
45	carboxylic acid biosynthetic process	GO:0046394	5
46	cell cycle phase transition	GO:0044770	5
47	electron transport chain	GO:0022900	5
48	GTP catabolic process	GO:0006184	5
49	GTP metabolic process	GO:0046039	5
50	guanosine-containing compound catabolic process	GO:1901069	5
51	guanosine-containing compound metabolic process	GO:1901068	5
52	mitotic cell cycle phase transition	GO:0044772	5
53	monocarboxylic acid biosynthetic process	GO:0072330	5
54	organic acid biosynthetic process	GO:0016053	5
55	positive regulation of cell activation	GO:0050867	5
56	positive regulation of leukocyte activation	GO:0002696	5
57	positive regulation of lymphocyte activation	GO:0051251	5
58	regulation of protein stability	GO:0031647	5
59	respiratory electron transport chain	GO:0022904	5
60	antigen processing and presentation of exogenous antigen	GO:0019884	4
61	antigen processing and presentation of exogenous peptide antigen	GO:0002478	4
62	antigen processing and presentation of exogenous peptide antigen via MHC class II	GO:0019886	4
63	antigen processing and presentation of peptide antigen via MHC class II	GO:0002495	4
64	antigen processing and presentation of peptide or polysaccharide antigen via MHC class II	GO:0002504	4
65	cellular component biogenesis	GO:0044085	4
66	cellular response to topologically incorrect protein	GO:0035967	4
67	cellular response to unfolded protein	GO:0034620	4
68	endoplasmic reticulum unfolded protein response	GO:0030968	4
69	ER-nucleus signaling pathway	GO:0006984	4
70	fatty acid biosynthetic process	GO:0006633	4
71	fatty acid derivative metabolic process	GO:1901568	4
72	icosanoid metabolic process	GO:0006690	4
[illegible]	[illegible]	[illegible]	[illegible]

Cellular Component of Up-regulated Genes in Marrest

	Term	GO ID	Count
1	extracellular vesicular exosome	GO:0070062	35
2	extracellular region	GO:0005576	27
3	extracellular space	GO:0005615	26
4	cytosol	GO:0005829	19
5	extracellular matrix	GO:0031012	10
6	integral component of plasma membrane	GO:0005887	10
7	cell surface	GO:0009986	8
8	proteinaceous extracellular matrix	GO:0005578	8
9	endoplasmic reticulum membrane	GO:0005789	7
10	Golgi membrane	GO:0000139	7
11	blood microparticle	GO:0072562	6
12	receptor complex	GO:0043235	6
13	adherens junction	GO:0005912	5
14	anchoring junction	GO:0070161	5
15	collagen trimer	GO:0005581	5
16	cytoplasmic vesicle membrane	GO:0030659	5
17	cytoplasmic vesicle part	GO:0044433	5
18	Golgi lumen	GO:0005796	5
19	MHC protein complex	GO:0042611	5
20	neuronal cell body	GO:0043025	5
21	vacuolar part	GO:0044437	5
22	vesicle membrane	GO:0012506	5
23	chromosomal region	GO:0098687	4
24	coated vesicle membrane	GO:0030662	4
25	endocytic vesicle membrane	GO:0030666	4
26	endosomal part	GO:0044440	4
27	ER to Golgi transport vesicle membrane	GO:0012507	4
28	growth cone	GO:0030426	4
29	lysosomal lumen	GO:0043202	4
30	site of polarized growth	GO:0030427	4
31	transport vesicle membrane	GO:0030658	4
32	ubiquitin ligase complex	GO:0000151	4
33	vacuolar lumen	GO:0005775	4
34	clathrin-coated endocytic vesicle membrane	GO:0030669	3
35	clathrin-coated vesicle membrane	GO:0030665	3
36	cullin-RING ubiquitin ligase complex	GO:0031461	3
37	cytoplasmic ribonucleoprotein granule	GO:0036464	3
38	heterochromatin	GO:0000792	3
39	integral component of endoplasmic reticulum membrane	GO:0030176	3
40	integral component of lumenal side of endoplasmic reticulum membrane	GO:0071556	3
41	integral component of mitochondrial membrane	GO:0032592	3
42	MHC class II protein complex	GO:0042613	3
43	mitochondrial membrane part	GO:0044455	3
44	motile cilium	GO:0031514	3
45	ribonucleoprotein granule	[illegible]	[illegible]
46	ribosomal subunit	[illegible]	[illegible]
47	ribosome	[illegible]	[illegible]
48	sperm part	[illegible]	[illegible]
49	trans-Golgi network membrane	[illegible]	[illegible]
50	acrosomal membrane	[illegible]	[illegible]
51	axolemma	[illegible]	[illegible]
52	chromatoid body	[illegible]	[illegible]
53	condensed chromosome, centromeric [illegible]	[illegible]	[illegible]
54	condensed nuclear chromosome, centromeric region	[illegible]	[illegible]
55	costamere	[illegible]	[illegible]
56	Cul3-RING ubiquitin ligase complex	[illegible]	[illegible]
57	cytosolic large ribosomal subunit	[illegible]	[illegible]
58	endosome lumen	[illegible]	[illegible]
59	extrinsic component of cytoplasmic si[illegible] of plasma membrane	[illegible]	[illegible]
60	extrinsic component of plasma memb[illegible]	[illegible]	[illegible]
61	integral component of mitochondrial outer membrane	[illegible]	[illegible]
62	juxtaparanode region of axon	[illegible]	[illegible]
63	MHC class I protein complex	[illegible]	[illegible]
64	microbody membrane	[illegible]	[illegible]
65	neuron projection membrane	[illegible]	[illegible]
66	P granule	[illegible]	[illegible]
67	pericentric heterochromatin	[illegible]	[illegible]
68	peroxisomal membrane	[illegible]	[illegible]
69	proteasome core complex	[illegible]	[illegible]
70	proteasome core complex, alpha-subu[illegible] complex	[illegible]	[illegible]
71	RNA polymerase II transcription factor [illegible]	[illegible]	[illegible]
72	secretory granule membrane	[illegible]	[illegible]
73	sex chromosome	[illegible]	[illegible]
74	SNARE complex	[illegible]	[illegible]
75	XY body	[illegible]	[illegible]

Cellular Component of Up-regulated Genes in Oligospermia

	Term	GO ID	Count
1	contractile fiber part	GO:0044449	6
2	axon	GO:0030424	5
3	sarcomere	GO:0030017	3
4	sarcoplasmic reticulum	GO:0016529	3
5	Z disc	GO:0030018	3
6	axon initial segment	GO:0043194	2
7	extrinsic component of cytoplasmic side of plasma membrane	GO:0031234	2
8	organelle subcompartment	GO:0031984	2
9	T-tubule	GO:0030315	2

Cellular Component of Up-regulated Genes in Teratospermia

	Term	GO ID	Count
1	nucleolus	GO:0005730	17
2	synapse part	GO:0044456	8
3	synaptic membrane	GO:0097060	8
4	postsynaptic membrane	GO:0045211	7
5	transcription factor complex	GO:0005667	6
6	cell-cell junction	GO:0005911	5
7	chromosomal region	GO:0098687	4
8	synapse	GO:0045202	4
9	chromosome, telomeric region	GO:0000781	3
10	primary cilium	GO:0072372	3
11	trans-Golgi network	GO:0005802	3
12	Z disc	GO:0030018	3
13	connexon complex	GO:0005922	2
14	exon-exon junction complex	GO:0035145	2

Molecular Function of Up-regulated Genes in Marrest

	Term	GO ID	Count
1	growth factor activity	GO:0008083	7
2	enzyme activator activity	GO:0008047	6
3	growth factor binding	GO:0019838	6
4	bitter taste receptor activity	GO:0033038	5
5	glycosaminoglycan binding	GO:0005539	5
6	hormone activity	GO:0005179	5
7	oxidoreductase activity, acting on CH-OH group of donors	GO:0016614	5
8	oxidoreductase activity, acting on the CH-OH group of donors, NAD or NADP as acceptor	GO:0016616	5
9	taste receptor activity	GO:0008527	5
10	amide binding	GO:0033218	4
11	antigen binding	GO:0003823	4
12	cell adhesion molecule binding	GO:0050839	4
13	heparin binding	GO:0008201	4
14	insulin-like growth factor binding	GO:0005520	4
15	iron ion binding	GO:0005506	4
16	peptide antigen binding	GO:0042605	4
17	peptide binding	GO:0042277	4
18	serine-type endopeptidase activity	GO:0004252	4
19	steroid dehydrogenase activity	GO:0016229	4
20	steroid dehydrogenase activity, acting on the CH-OH group of donors, NAD or NADP as acceptor	GO:0033764	4
21	cargo receptor activity	GO:0038024	3
22	collagen binding	GO:0005518	3
23	double-stranded DNA binding	GO:0003690	3
24	frizzled binding	GO:0005109	3
25	histone binding	GO:0042393	3
26	monooxygenase activity	GO:0004497	3
27	scavenger receptor activity	GO:0005044	3
28	structural constituent of cytoskeleton	GO:0005200	3
29	structural constituent of ribosome	GO:0003735	3
30	unfolded protein binding	GO:0051082	3
31	1-phosphatidylinositol-3-kinase activity	GO:0016303	2
32	acid-thiol ligase activity	GO:0016878	2
33	carboxypeptidase activity	GO:0004180	2
34	CoA-ligase activity	GO:0016405	2
35	ferrous iron binding	GO:0008198	2
36	glutathione transferase activity	GO:0004364	2
37	histone kinase activity	GO:0035173	2
38	insulin receptor binding	GO:0005158	2
39	insulin-like growth factor receptor binding	GO:0005159	2
40	intramolecular oxidoreductase activity	GO:0016860	2
41	intramolecular oxidoreductase activity, transposing C=C bonds	GO:0016863	2
42	ligase activity, forming carbon-sulfur bonds	GO:0016877	2
43	MHC class II receptor activity	GO:0032395	2
[illegible]	[illegible]	[illegible]	[illegible]

Molecular Function of Up-regulated Genes in Oligospermia

	Term	GO ID	Count
1	actin binding	GO:0003779	7
2	hormone activity	GO:0005179	4
3	oxygen binding	GO:0019825	3
4	cytokine binding	GO:0019955	3
5	tropomyosin binding	GO:0005523	2
6	neuropeptide hormone activity	GO:0005184	2
7	calmodulin-dependent protein kinase activity	GO:0004683	2

Molecular Function of Up-regulated Genes in Teratospermia

	Term		Count
1	sequence-specific DNA binding RNA polymerase II transcription factor activity	GO:0000981	6
2	transcription regulatory region sequence-specific DNA binding	GO:0000976	6
3	core promoter proximal region DNA binding	GO:0001159	4
4	core promoter proximal region sequence-specific DNA binding	GO:0000987	4
5	Ras GTPase binding	GO:0017016	4
6	RNA polymerase II core promoter proximal region sequence-specific DNA binding	GO:0000978	4
7	RNA polymerase II core promoter proximal region sequence-specific DNA binding transcription factor activity	GO:0000982	4
8	small GTPase binding	GO:0031267	4
9	channel regulator activity	GO:0016247	3
10	damaged DNA binding	GO:0003684	3
11	double-stranded DNA binding	GO:0003690	3
12	enhancer binding	GO:0035326	3
13	enhancer sequence-specific DNA binding	GO:0001158	3
14	phosphoric diester hydrolase activity	GO:0008081	3
15	protein kinase C binding	GO:0005080	3
16	Rab GTPase binding	GO:0017137	3
17	RNA polymerase II transcription factor binding	GO:0001085	3
18	alcohol dehydrogenase NADP+) activity	GO:0008106	2
19	aldo-keto reductase NADP) activity	GO:0004033	2
20	calcium-dependent phospholipid binding	GO:0005544	2
21	cGMP binding	GO:0030553	2
22	chemokine binding	GO:0019956	2
23	chemokine receptor activity	GO:0004950	2
24	cis-trans isomerase activity	GO:0016859	2
25	cyclic nucleotide binding	GO:0030551	2
26	gap junction channel activity	GO:0005243	2
27	G-protein coupled chemoattractant receptor activity	GO:0001637	2
28	mismatched DNA binding	GO:0030983	2
29	peptidyl-prolyl cis-trans isomerase activity	GO:0003755	2
30	protein self-association	GO:0043621	2
31	sodium channel regulator activity	GO:0017080	2
32	telomeric DNA binding	GO:0042162	2
33	wide pore channel activity	GO:0022829	2

KEGG Pathway of Up-regulated Genes in MArrest

	Term	ID	Count
1	MicroRNAs in cancer	hsa05206	19
2	Staphylococcus aureus infection	hsa05150	8
3	HTLV-I infection	hsa05166	7
4	Complement and coagulation cascades	hsa04610	6
5	Ovarian steroidogenesis	hsa04913	6
6	Steroid hormone biosynthesis	hsa00140	6
7	Taste transduction	hsa04742	6
8	Epstein-Barr virus infection	hsa05169	5
9	Mineral absorption	hsa04978	5
10	Neuroactive ligand-receptor interaction	hsa04080	5
11	Oocyte meiosis	hsa04114	5
12	Pertussis	hsa05133	5
13	Rheumatoid arthritis	hsa05323	5
14	Systemic lupus erythematosus	hsa05322	5
15	ABC transporters	hsa02010	4
16	Antigen processing and presentation	hsa04612	4
17	Cell cycle	hsa04110	4
18	Phagosome	hsa04145	4
19	Protein processing in endoplasmic reticulum	hsa04141	4
20	Aldosterone synthesis and secretion	hsa04925	3
21	Allograft rejection	hsa05330	3
22	Asthma	hsa05310	3
23	Autoimmune thyroid disease	hsa05320	3
24	Graft-versus-host disease	hsa05332	3
25	Inflammatory bowel disease (IBD)	hsa05321	3
26	Intestinal immune network for IgA production	hsa04672	3
27	Leishmaniasis	hsa05140	3
28	Longevity regulating pathway - multiple species	hsa04213	3
29	Propanoate metabolism	hsa00640	3
30	Protein digestion and absorption	hsa04974	3
31	Renin-angiotensin system	hsa04614	3
32	Type I diabetes mellitus	hsa04940	3
33	Viral myocarditis	hsa05416	3
34	Butanoate metabolism	hsa00650	2
35	Pentose phosphate pathway	hsa00030	2
36	Pyruvate metabolism	hsa00620	2
37	Steroid biosynthesis	hsa00100	2
38	Terpenoid backbone biosynthesis	hsa00900	2

KEGG Pathway of Up-regulated Genes in Oligospermia

	Term	GO ID	Count
1	Adrenergic signaling in cardiomyocytes	hsa04261	6
2	Calcium signaling pathway	hsa04020	6
3	cGMP-PKG signaling pathway	hsa04022	4
4	Dilated cardiomyopathy	hsa05414	4
5	Oxytocin signaling pathway	hsa04921	4
6	Arrhythmogenic right ventricular cardiomyopathy (ARVC)	hsa05412	3
7	Cardiac muscle contraction	hsa04260	3
8	Chemical carcinogenesis	hsa05204	3
9	Hypertrophic cardiomyopathy (HCM)	hsa05410	3
10	Long-term potentiation	hsa04720	3
11	PPAR signaling pathway	hsa03320	3
12	Type I diabetes mellitus	hsa04940	3

KEEG Pathway of Up-regulated Genes in Teratospermia

	Term	ID	Count
1	mRNA surveillance pathway	hsa03015	4
2	Morphine addiction	hsa05032	3

Cellular Component of Down-regulated Genes in Marrest

	Term	GO ID	Count
[illegible]	[illegible]	[illegible]	[illegible]

Cellular Component of Down-regulated Genes in Oligospermia

	Term	GO ID	Count
1	sperm part	GO:0097223	4
2	chromosomal region	GO:0098687	3
3	chromosome, centromeric region	GO:0000775	3
4	nuclear transcription factor complex	GO:0044798	3
5	acrosomal membrane	GO:0002080	2
6	acrosomal vesicle	GO:0001669	2
7	chromatoid body	GO:0033391	2
8	P granule	GO:0043186	2

Cellular Component of Down-regulated Genes in Teratospermia

	Term	GO ID	Count
1	extracellular vesicular exosome	[illegible]	[illegible]
2	cytosol	[illegible]	[illegible]
3	ribosomal subunit	[illegible]	[illegible]
4	cytosolic part	[illegible]	[illegible]
5	mitochondrion	[illegible]	[illegible]
6	adherens junction	[illegible]	[illegible]
7	anchoring junction	[illegible]	[illegible]
8	cell-substrate adherens junction	[illegible]	[illegible]
9	cell-substrate junction	[illegible]	[illegible]
10	focal adhesion	[illegible]	[illegible]
11	ribosome	[illegible]	[illegible]
12	cytosolic large ribosomal subunit	[illegible]	[illegible]
13	large ribosomal subunit	[illegible]	[illegible]
14	small ribosomal subunit	[illegible]	[illegible]
15	endoplasmic reticulum membrane	[illegible]	[illegible]
16	vacuolar part	[illegible]	[illegible]
17	ciliary part	[illegible]	[illegible]
18	cytosolic small ribosomal subunit	[illegible]	[illegible]
19	lysosomal lumen	[illegible]	[illegible]
20	mitochondrial inner membrane	[illegible]	[illegible]
21	mitochondrial membrane part	[illegible]	[illegible]
22	organelle inner membrane	[illegible]	[illegible]
23	vacuolar lumen	[illegible]	[illegible]
24	external side of plasma membrane	[illegible]	[illegible]
25	integral component of endoplasmic reticulum membrane	[illegible]	[illegible]
26	integral component of organelle membrane	[illegible]	[illegible]
27	mitochondrial matrix	[illegible]	[illegible]
28	secretory granule	[illegible]	[illegible]
29	side of membrane	[illegible]	[illegible]
30	ciliary basal body	[illegible]	[illegible]
31	cytoplasmic membrane-bounded vesicle lumen	[illegible]	[illegible]
32	platelet alpha granule lumen	[illegible]	[illegible]
33	secretory granule lumen	[illegible]	[illegible]
34	vesicle lumen	[illegible]	[illegible]
35	chromosome, centromeric region	[illegible]	[illegible]
36	growth cone	[illegible]	[illegible]
37	integral component of lumenal side of endoplasmic reticulum membrane	[illegible]	[illegible]
38	mitochondrial small ribosomal subunit	[illegible]	[illegible]
39	organellar small ribosomal subunit	[illegible]	[illegible]
40	oxidoreductase complex	[illegible]	[illegible]
41	site of polarized growth	[illegible]	[illegible]
42	spindle	[illegible]	[illegible]
43	centriolar satellite	[illegible]	[illegible]
[illegible]	[illegible]	[illegible]	[illegible]

Molecular Function of Down-regulated Genes in Marrest

	Term	GO ID	Count
[illegible]	[illegible]	[illegible]	[illegible]

Molecular Function of Down-regulated Genes in Oligospermia

	Term	GO ID	Count
1	calcium ion binding	GO:0005509	8
2	histone deacetylase binding	GO:0042826	3
3	isoprenoid binding	GO:0019840	2
4	nuclear hormone receptor binding	GO:0035257	3
5	protein binding involved in protein folding	GO:0044183	2
6	retinoic acid binding	GO:0001972	2
7	retinoid binding	GO:0005501	2
8	TBP-class protein binding	GO:0017025	2

Molecular Function of Down-regulated Genes in Teratospermia

	Term	GO ID	Count
1	structural constituent of ribosome	GO:0003735	18
2	guanyl nucleotide binding	GO:0019001	6
3	guanyl ribonucleotide binding	GO:0032561	6
4	GTPase activity	GO:0003924	5
5	isomerase activity	GO:0016853	5
6	cis-trans isomerase activity	GO:0016859	3
7	cytokine binding	GO:0019955	3
8	cytokine receptor activity	GO:0004896	3
9	GDP binding	GO:0019003	3
10	hydrogen ion transmembrane transporter activity	GO:0015078	3
11	peptidyl-prolyl cis-trans isomerase activity	GO:0003755	3
12	translation elongation factor activity	GO:0003746	3
13	translation factor activity, nucleic acid binding	GO:0008135	3
14	translation regulator activity	GO:0045182	3
15	virus receptor activity	GO:0001618	3
16	alpha-tubulin binding	GO:0043014	2
17	ARF GTPase activator activity	GO:0008060	2
18	carbohydrate transmembrane transporter activity	GO:0015144	2
19	carbohydrate transporter activity	GO:1901476	2
20	cytochrome-c oxidase activity	GO:0004129	2
21	estradiol 17-beta-dehydrogenase activity	GO:0004303	2
22	heme-copper terminal oxidase activity	GO:0015002	2
23	histone kinase activity	GO:0035173	2
24	intramolecular transferase activity	GO:0016866	2
25	organic hydroxy compound transmembrane transporter activity	GO:1901618	2
26	oxidoreductase activity, acting on a heme group of donors	GO:0016675	2
27	oxidoreductase activity, acting on a heme group of donors, oxygen as acceptor	GO:0016676	2
28	oxidoreductase activity, acting on the aldehyde or oxo group of donors, disulfide as acceptor	GO:0016624	2
29	oxidoreductase activity, acting on the CH-NH group of donors	GO:0016645	2
30	peptidase activator activity	GO:0016504	2
31	protein kinase A binding	GO:0051018	2
32	Rac GTPase binding	GO:0048365	2
33	rRNA binding	GO:0019843	2
34	steroid dehydrogenase activity	GO:0016229	2
35	steroid dehydrogenase activity, acting on the CH-OH group of donors, NAD or NADP as acceptor	GO:0033764	2
36	threonine-type endopeptidase activity	GO:0004298	2
37	threonine-type peptidase activity	GO:0070003	2
38	translation activator activity	GO:0008494	2
39	translation regulator activity, nucleic acid binding	GO:0090079	2

KEGG Pathway of Up-regulated Genes in MArrest

	Term	ID	Count
1	Metabolic pathways	hsa01100	7
2	Neuroactive ligand-receptor interaction	hsa04080	6
3	PI3K-Akt signaling pathway	hsa04151	6
4	Focal adhesion	hsa04510	5
5	HIF-1 signaling pathway	hsa04066	4
6	Huntington's disease	hsa05016	4
7	Arachidonic acid metabolism	hsa00590	3
8	ECM-receptor interaction	hsa04512	3
9	Glycolysis / Gluconeogenesis	hsa00010	3
10	Nicotine addiction	hsa05033	3
11	Retrograde endocannabinoid signaling	hsa04723	3
12	Biosynthesis of amino acids	hsa01230	2
13	Central carbon metabolism in cancer	hsa05230	2
14	Fat digestion and absorption	hsa04975	2
15	Fatty acid biosynthesis	hsa00061	2
16	Linoleic acid metabolism	hsa00591	2
17	PPAR signaling pathway	hsa03320	2
18	Proximal tubule bicarbonate reclamation	hsa04964	2
19	Starch and sucrose metabolism	hsa00500	2
20	Thyroid hormone synthesis	hsa04918	2
21	Butirosin and neomycin biosynthesis	hsa00524	1

KEGG Pathway of Down-regulated Genes in Oligospermia

	Term	GO ID	Count
1	Metabolic pathways	hsa01100	10
2	Inositol phosphate metabolism	hsa00562	3
3	Phosphatidylinositol signaling system	hsa04070	3
4	Fatty acid biosynthesis	hsa00061	2
5	Propanoate metabolism	hsa00640	2
6	Pyruvate metabolism	hsa00620	2
7	Cysteine and methionine metabolism	hsa00270	2

KEEG Pathway of Down-regulated Genes in Teratospermia

	Term	ID	Count
1	Ribosome	hsa03010	16
2	Alzheimer's disease	hsa05010	6
3	Oxidative phosphorylation	hsa00190	5
4	Parkinson's disease	hsa05012	5
5	Huntington's disease	hsa05016	5
6	Non-alcoholic fatty liver disease (NAFLD)	hsa04932	4
7	Antigen processing and presentation	hsa04612	3
8	Pentose phosphate pathway	hsa00030	2
9	Propanoate metabolism	hsa00640	2
10	Glycosaminoglycan degradation	hsa00531	2

Supplementary Table S3. Differentially expressed genes of Post Meiotic Arrest, Meiotic Arrest, Pre Meiotic Arrest and Sertoly Cell Only Syndrome

Up- Regulated Genes							Down- Regulated Genes					
**Post MA**	**MA**		**Pre MA**		**SCOS**		**Post MA**	**MA**	**Pre MA**		**SCOS**	
AAMDC	ACAT2	STAG1	ABCA9	PSME1	ABHD4	PHKG1	ABHD1	ACSBG2	ABHD1	SMARCE1	ABHD1	PRM1
ABCA10	ACSS2	STEAP1B	ADAM28	PTPRD	ABRA	phyhip	ACTG2	ACTL7A	ACE	SMCP	ACSBG2	PRM2
ABCA6	ACSS3	STRADB	ADAMTS12	PTPRE	ACKR4	PIK3C3	ADAM29	ACTRT2	ACR	SPACA3	ACTL7A	PRR30
ABCA8	AK3L1	STX10	ADAMTS5	PTPRM	ACOT2	PIN4P1	ADAM7	ADAM29	ACRBP	SPACA4	ACTL7B	Prss21
ABCA9	ANAPC10	SYCP3	AKT3	RAB41	ACSS1	PLA2G4A	AKAP1	AKAP1	ACRV1	SPANXB1	ADAD1	PSMG1
ACSM3	ANP32A	TAF1D	AMFR	RBMS3	ADAMTS10	PLA2G7	ANKRD13B	AKAP4	ACSBG2	SPATA16	ADAM18	PWP1
ADAMTS5	ANP32B	TAS2R3	AMTN	RBMY2EP	ADAMTS5	PLCB4	ANKRD53	ANKRD53	ACTL7A	SPATA3	ADAM29	RACGAP1
ANKRD20A9P	ANXA1	TAS2R45	ANAPC5	RBP1	ADCY1	PLEK2	ANO1	ARMC12	ACTL7B	SPATA9	ADORA3	RBM26-AS1
ANTXR1	ARHGAP12	TAS2R50	ANTXR2	RECK	AFG3L2	PLP1	APBB3	ATP1A4	ADAM2	SPATS1	AKAP4	RBMXL2
ATP5L2	ARHGEF6	TCN2	ANXA1	RFPL4A	AGER	plxna1	ARMC12	BANF2	ADAM29	SPERT	ALDH1A2	RFC3
C3orf35	ASB13	TET2	AP1G1	RGS1	AKT1	PLXNA3	ATP1A4	BPIFA3	ADAM3A	ST6GALNAC2	ALS2CR11	RGS22
C6orf203	ATG2A	TEX11	APCDD1	ROBO1	ANGPTL1	PLXNC1	ATP8B3	C10orf62	ADAM5	STAT4	ANKRD13A	RIMBP3C
C8orf49	ATM	TMEM106B	ARHGEF6	ROBO2	ANKRD33B	POLR2F	BAP1	C16orf82	ADAMTS3	SYT7	ANKRD7	RIMS1
CAV2	ATP1B3	TMTC3	ARMCX1	RPA4	ANXA1	PP12719	C16orf82	C17orf105	ADRA1D	TACR3	AQP5	ROPN1
CBWD2	AZI2	TMX1	ARMCX3	RPS27L	AP1S2	PP14571	C19orf45	C17orf74	AHCYL1	TAF4	ARMC12	ROPN1B
CLN5	BCCIP	TncRNA	ASPHD2	RPS4Y1	APOBEC2	PPFIBP2	C1orf192	C19orf45	AKAP4	TEKT2	ARMC4	ROPN1L
COMMD6	BCORL1	TOP2B	AYTL1	RPS6KA3	APOL3	PPP1R1A	C20orf173	C1orf111	ALOX15	TEKT3	ART3	RSHL1
COX16	BET1	TRAF3	B2M	RRS1	ARHGEF6	PRAGMIN	C2orf57	C2orf57	ANKRD5	TES	ASAP2	RUBCNL
CRYGS	C1D	TRNT1	BARHL1	RTP4	ATP8B4	PRKACB	C5orf60	C3orf30	ANKRD55	TEX38	ASRGL1	SAP30
CSRNP3	C6orf166	TUBB2A	BCL6B	SAMD9L	ATRX	PRLR	C7orf34	C5orf60	APOBEC4	TFAP2A	ASTN2-AS1	SEC23B
CTSK	C9orf78	TUBB2B	BHLHB2	SAMHD1	B2M	PROK1	C8orf74	C7orf34	AQP5	TMC5	ATP1A3	sec24a
DKFZP779L18	CASK	UBLCP1	BIVM	SCRG1	BRCC3	PRPS2	C9orf131	C8orf74	BANF2	TMC7	ATP23	SEPTIN10
DNM3OS	CAV2	UIMC1	BNIP3	SDCBP	BTN3A3	PRR4	CABLES2	C9orf131	BCAT1	TMCO5A	BAG6	SEPTIN12
DOCK10	CBFB	USP51	C10orf116	SERPINI1	C10orf71	PRRG4	CATSPER1	C9orf173	BCL2L14	TMEM144	BAIAP2L1	SESN3
DPM1	CBR4	UTP11L	C12orf29	SHE	C15orf52	PSPN	CATSPER4	CCIN	BPIFA3	TMEM189	BANF2	SLC26A8
DRAM2	CBWD2	VAMP4	C12orf41	SIN3B	c19orf60	PTPN11	CATSPERG	CLPB	BRIP1	TMEM190	BCL2L14	SLC5A6
ERAP2	CCNG1	VAMP7	C1orf21	SLC16A14	C21orf62	PWAR6	CCDC114	CPA5	C10orf62	TMEM31	BIRC5	SLC6A16
ESCO2	CDC37L1	VNN2	C1orf57	SLC25A4	C5	QDPR	CCDC116	CXCL16	C13orf28	TMPRSS6	BOLL	SMCP
FAM103A1	CDS2	WDR75	C6	SLC37A3	C7	rab22a	CCDC159	DNAH17	C14orf129	TNFAIP6	BPIFA3	SNHG11
FLJ38894	CENPC	WNT11	C6orf203	SLC38A1	CACNA1S	RAB5B	CCER1	EIF4E	C16orf78	TNP1	BRDT	SOX30
FLRT2	CNOT7	XIST	C9orf130	SLC8A3	CALB2	RARRES2	CEL	FAM166A	C17orf74	TNP2	BTG4	SPACA4
FRG1	COPS2	ZEB2	C9orf78	SLCO4C1	CALCRL	RBMS3	CFAP74	FAM187B	C1orf108	TOX3	C10orf62	SPAG5
FZD4	COX16	ZFAND1	CAPN13	SLFN5	CAMK2N1	RGS14	CHD5	FAM205A	C1orf111	TPPP2	C10orf68	SPAG6
GLO1	CPM	ZNF141	CASP4	SMC5	CAPN2	RNF152	CHRM4	FAM71A	C1orf158	TREML2	c11orf70	SPANXB1
GPR21	CRYAB	ZNF189	CAV2	SNHG1	CAPN5	RNMT	CLPB	FAM71B	C1orf194	TRPM5	C12orf25	SPATA3
GPR52	CTPS2	ZNF22	CBR3	SNORD113-3	cass4	ROBO2	CNN1	FAM71F1	C1orf34	TSACC	C16orf71	SPATA8
GPR75	CXYorf2	ZNF260	CCDC93	SNORD113-4	CCDC102A	RP13-347D8.3	CNTN2	FANCD2OS	C1orf94	TSKS	C16orf78	SPC25
GSTA1	DCUN1D5	ZNF37A	CCL2	SNORD114-2	CCDC138	RPS9	COX6B2	FBXO24	C20orf10	TSPAN1	C17orf105	SPCS3
GSTA3	DDEF1	ZNF430	CD14	SNORD114-26	CCNG1	RTN2	CPA5	FER1L5	C20orf141	TSSK2	C17orf39	SPINK2
GTF2A2	DDIAS	ZNF486	CDH19	SNORD114-3	CD14	SAMD9	CPNE2	FNDC8	C20orf195	TTBK2	C17orf51	SPRTN
HEXA-AS1	DICER1	ZNF502	CENPI	SNORD114-6	CD36	SAMD9L	CPTP	FSCB	C20orf85	TUBA8	C17orf74	STAG3
HMGB1	DLEU2	ZNF503	CFI	SNORD56B	CD48	SAPS2	CSNK1G2	FSCN3	C22orf23	TULP2	C1orf105	STK31
HMGCR	DMKN	ZNF525	CFL2	SNORD73A	CD99	SDS	DCST1	FTMT	C3orf22	UBQLN3	C1orf158	SYCP1
HMGCS1	DNAJB11	ZNF641	CHI3L1	SNORD78	CDK5	SEPP1	DCST2	GAPDHS	C4orf35	UGT1A1	C1orf194	SYT6
HNRNPU	DNAJC12	ZNF649	CHRNA1	SNX22	CEBPA-AS1	SFTPD	DEFB126	GGN	C5AR1	UNQ9391	C1QTNF3	TARDBP
HSD17B3	DPM1	ZNF682	CHST11	SPTLC3	CFH	SGCE	DNAH1	GSG1	C5orf60	UPK3A	C1R	TCAM1P
HSD17B6	DPPA3P2	ZNF737	CLCN5	SS18L1	CFI	SH2B1	DNAH17	GUCA1A	C6orf10	USF1	C20orf85	TCP10L
IGSF6	EIF1AY	ZNF770	CLEC2B	STMN2	CFLAR	SH3TC1	DNAI2	KIF2B	C6orf204	USP2	C22orf23	TCP11
IPO7	ENTPD1	ZNF845	CMYA5	TACSTD1	CHL1	SHD	DOC2A	KLHL10	C7orf34	WBSCR28	C5orf60	TCTE1
KLHL9	ENY2		CNKSR3	TBC1D13	CIDEC	SLC15A2	DOT1L	LELP1	C7orf62	ZC2HC1B	C6orf10	TDRG1
KRT6A	EPB41L4A		COL27A1	TBC1D9	CKMT2	SLC25A14	DPEP3	LINC00521	C9orf131	ZDHHC19	C7orf31	TEKT3
LAMA4	ESCO2		CPA3	TBL1XR1	CLEC10A	SLC27A1	DRC7	LOC100132147	CA13		C9orf131	TEX14
LHCGR	ETFDH		CREB5	TET2	CLEC4M	SLC30A3	DUS1L	LYZL1	CABS1		C9orf72	TEX30
LIMD1-AS1	EXOC6		CRYZ	TEX11	COBLL1	SLC38A1	DUSP1	LYZL6	CAMKK2		CALR3	TEX38
LINC00328	FAM103A1		CSNK1E	TLOC1	COL27A1	SLC44A1	DXO	MROH7-TTC4	CAPN11		CAPN11	TFDP3
LINC00643	FAM69A		CTHRC1	TMEM158	CPA3	SLC4A4	FAM163A	MS4A6E	CAPS		CAPZA3	TKTL1
LIPT1	FLJ38894		CTNNA3	TMEM43	CPED1	SLFN5	FAM166A	NT5C1B	CAPZA3		CASC5	TKTL2
LOC100130428	FLJ44451		CTSA	TncRNA	CRK	snca	FAM183B	OAZ3	CCDC126		CCDC136	TMEM99
LOC100131541	FNBP1		CTSG	TRAF3	CRYAB	SNX2	FAM186B	ODF1	CCIN		CCDC65	TMUB2
LOC100132099	FPR1		CTSK	TRO	CRYZ	SORBS2	FAM187B	ODF3L1	CCR9		CCIN	TNFAIP6
LOC100287869	FRG1		CUL5	TSPO	CSDC2	SOX11	FAM205A	PHOSPHO1	CCT8L2		CCNA1	TNP1
LOC731157	FUSIP1		CXorf39	UBE2A	CTBS	SP100	FAM205B	PPP1R32	CDC14A		CCNB2	TP53I3
MALAT1	FXC1		CXorf48	USP31	CTNNB1	SPARCL1	FAM71E2	PRM1	CDY1		CDC20B	TPPP2
MAML2	GAK		CYP7B1	USP9Y	CTSC	ST3GAL4	FAM71F1	PRM2	CLPB		CDKN3	TPTE
METTL15	GAS5		DAZ1	VCX	CTSD	STAG2	FBXO24	PROCA1	CMTM2		CEP72	TPTE2P3
METTL21EP	GDAP1		DCK	VCY	CTSK	STEAP4	FBXW10	PRR30	CNTF		CERS3	TRIM36
MGARP	GFI1		DDX3Y	VIPR1	CUX1	TAS2R14	FER1L5	PRSS54	CRISP2		CETN1	TRMT13
MGC24103	GGA2		DDX58	VTN	CYBB	TAS2R4	FOS	RNF151	CSNK1G2		CETN3	TSACC
MIR107	GNPDA2		DNAJB11	WDR41	CYLD	TAX1BP3	FOXM1	SAMD4A	CST8		CFAP61	TSP50
MIR128-1	GPR21		DNASE1L3	WNT8B	CYP8B1	TCF3	FSCB	SIRPD	CTNNA3		CKS2	TSPAN2
MIR145	GSTA1		DSCR3	WTAP	D4S234E	TCL1A	GAPDHS	SLC36A3	CXorf27		CMTM2	TTC25
MIR154	GSTA3		DSEL	ZEB2	DAB2	TERF1	GEMIN4	SLFNL1	CYLC1		CNTD1	TUBA3E
MIR15A	GTPBP10		DTNA	ZNF226	DAO	TET2	GGN	SMCP	CYLC2		COL10A1	TUBE1
MIR18A	HAT1		EDN1	ZNF277	DCP1B	TFPI	GLT1D1	SPACA4	DCC		CRISP2	TULP2
MIR197	HERC4		ELAVL3	ZNF641	DDR2	THOC2	GLT6D1	SPATA18	DDI1		CT45A10	UBQLN3
MIR199A2	HIGD1A		ENPP2	ZNF770	DEF8	THPO	GPR64	SPATA20	DGKA		CXorf27	VCY
MIR21	HIST1H2AC		FAM114A1	ZNF805	DIRAS3	THY1	GPX5	SPATA3	DKKL1		CXorf61	WBSCR28
MIR214	HIST1H2BH		FAM129A		DNAH1	TM4SF18	GRIK5	SPATA31A7	DNAH17		CYB5D1	WEE1
MIR218-1	HIST2H2AB		FAM20C		DPT	TMEM53	GUCA1A	SPATA31C1	DNAJC5B		CYLC1	XAGE1B
MIR23B	HLA-DPA1		FARP1		DTNA	TMPRSS4	HEATR9	SPERT	DNM1		DAZ1	YBX2
MIR27B	HLA-DQA1		FAT1		DUSP26	TNFSF10	HIPK4	ST6GALNAC2	DPH4		DDX25	YPEL1
MIR29C	HSD11B1		FEM1B		EDDM3A	TPO	IL13	SUN5	DRC7		DDX39A	zfp64
MIR30E	HSD17B11		FHOD3		EDEM2	TRBV19	INPP1	TBATA	DUSP13		DDX4	ZMYND10
MIR31	HSPA8		FLJ38894		EIF2AK2	TREM1	KCTD19	TEX38	EAF1		DESI2	ZNF254
MIR32	HSPB3		FLJ39632		EMCN	TRIM16	KIAA1683	THEG	EDG5		DKFZp434K19	ZPBP2
MIR503	IER2		FLJ44451		ENDOU	TRIM2	KIF2B	TMEM31	EHD1		DKKL1	
MIR509	IER3		FLRT2		EPX	TRIM38	KIR2DS2	TPPP2	EIF4E		DNAH17	
MIR99A	IFIT1		GABRG1		ERAP2	TRIM63	KLHL10	TRIM42	EOMES		DNAH8	
MIR99AHG	IL16		GABRP		F2R	TTC37	KSR1	TSPAN1	ETFDH		DNAI1	
MIRLET7A2	IL1A		GAK		FAM13A	TUBA1C	LOC102725072	TSPAN16	EZH1		DRC7	
MIRLET7C	IL1RAP		GATA2		FAM167B	UBE2D3	LOC157740	TSSK3	FAM154A		DTL	
MIRLET7F1	ING4		GBP1		FAM46A	UGDH	LRRC74A	TSSK6	FAM166A		DYNC2LI1	
MIRLET7G	IPO7		GCLC		FBLN5	USP38	LY6G5B	TXNDC2	FAM205A		DYNLRB2	
MPLKIP	JAM3		GLO1		fgf22	VNN2	LYZL1	UBQLN3	FAM230C		EIF5A2	
MSMO1	KCNK17		GPR128		FGL2	WISP2	LYZL6	UBQLNL	FAM46C		ELAVL2	
NKAPP1	KCNS3		GRIK2		FLJ36840	ZBED1	MMEL1	WBSCR28	FAM71A		FAM112B	
NRK	KIAA0484		HERC4		FMO1	ZEB2	MROH5	ZC2HC1B	FAM71F1		FAM166A	
OCLM	KIAA1199		HIPK3		FRRS1	ZKSCAN1	MROH7-TTC4	ZDHHC19	FCRL2		FAM205A	
OPCML	KIF1C		HIST1H4H		FXYD6	ZNF189	MUC1	ZNF683	FEM1B		FAM230B	
OR10G8	KIR2DL4		HIVEP2		GADD45G	ZNF426	NEFM	ZNRF4	FHL5		FAM230C	
OR52H1	KLHL9		HLA-DMB		GAS6	ZNF560	NR4A2		FLJ20160		FAM71A	
OR8G2	KRR1		HLA-DPB1		GBP1	ZNF737	NSUN4		FLJ40244		FAM71F1	
PCDH18	KRT6A		HLA-DQA1		GGA2	ZNF738	NT5C1B		FNDC8		FKBP6	
PCDHB18	KRTAP19-8		HLA-DRA		GLIPR1	ZNF91	NUP210P1		FSCN3		FLJ23049	
PDE4D	KYAT3		HLA-G		GNL1		ODF3		FTMT		FLJ25758	
PNPLA8	LAMTOR3		HMBOX1		GPD1		ODF3L1		GABRB1		FNDC8	
PP12719	LINC00328		HMGB1		GPM6A		OR2H1		GAPDHS		FSCN3	
PRKAA2	LITAF		HMGCLL1		GPR1		PCSK4		GFRA2		FTMT	
PSMA2	LOC100130428		HOMER1		GPX8		PHKG2		GGN		FUT1	
PWAR6	LOC100131541		HSD17B14		GSTM1		PILRB		GINS1		GALNT18	
RPL21P28	LOC283481		HSPB2		GstT1		PLA2G6		GP2		GAPDHS	
RPL23P8	LOC389834		ICK		GZMK		PLCD4		GPR135		GCK	
RPL31	LOC63920		IER2		HEXB		POLE		GPX5		GGN	
RPL6	LRIF1		IFI44L		HIST1H2AC		PPM1J		GRIA2		GK2	
RPS3A	LRRN3		ITGB7		HLA-DPA1		PPP1R32		GRIA3		GMPS	
SC5D	LSM5		ITM2A		HLA-DPB1		PRKCZ		GSG1		GSG1	
SCARNA7	LTV1		KCNAB3		HLA-DQA1		PROCA1		GTSF1L		GTF2A2	
SCARNA9	LYSMD3		KIAA1217		HLA-DRA		PRPH		HDAC11		GTSF1	
SDHD	MAD2L1		KIAA1468		HMGA1		PRR30		HK2		GTSF1L	
SLC10A5	MAGOHB		KLF1		HNMT		PRSS54		HMGB4		H3F3B	
SLIRP	MALAT1		KLF6		HOMER1		REC8		HOOK1		hCG_1815491	
SMAD2	MAP1B		KLHDC5		HOXA10		RGL2		HSFY1		HELLS	
SMN1	MAPKAPK5-AS1		KRR1		HPGD		RND2		HSPG2		HIP1	
SNCA	MARS		KRTAP19-8		HPGDS		RNF151		IL1F5		HIST1H1T	
SNORD13P2	MEGF8		LARP6		HSD17B11		SIRPD		IPO7		HIST1H2AA	
SNORD75	METTL15		LEPREL1		HSD3B2		SLC36A3		IRGC		HIST3H3	
SNORD78	MGC24103		LHCGR		IFI44L		SLC4A11		ISG20L2		HMGB3	
SNX2	MIR107		LINC00670		IFITM1		SLC7A7		JRKL		HOOK1	
STRADB	MIR145		LIPH		IGF1		SLFNL1		KIAA1324		HRASLS5	
TAPT1-AS1	MIR15A		LOC100131541		IGFBP5		SMCP		KLK13		HSF5	
TAS2R10	MIR199A2		LOC100131943		IGSF21		SPACA4		KLK5		HSFY1	
TAS2R14	MIR21		LOC100132686		IL12A		SPATA18		KRT17		HSPA2	
TAS2R19	MIR27B		LOC399959		IL1RL2		SPATA20		KRT23		HSPB9	
TAS2R20	MIR30E		LOC644353		IL32		SPATA31C1		LAMP3		HUS1	
TAS2R3	MIR32		LOC729852		ITPR1		SPERT		LATS2		INPP4A	
TAS2R31	MIR503		LOC730102		ITPR2		SPTBN2		LECT2		INTS6	
TAS2R45	MIR509		LRRC17		JMJD2A		SUN5		LELP1		IRGC	
TAS2R50	MIR99A		LRRN2		KAT6B		TBATA		LINC00238		KAT6B	
TET2	MIRLET7A2		LRRTM3		KCNQ1		TBC1D3G		LINC00301		KCTD19	
THSD7A	MIRLET7C		MAGEA8		KEL		TBL3		LOC136242		KDM4D	
TMA7	MIRLET7F1		MAGEB16		KHSRP		TEX38		LOC157740		KIAA1666	
TMPPE	MIRLET7G		MAGEB6		KIDINS220		THBS1		LOC440896		KNTC1	
TMX1	MLC1		MARCH_1		KYAT3		THEG		LOC728450		KPNA2	
TOP2B	MMP26		MDH1		LAMTOR3		TNC		LRIT1		LDHAL6B	
TRMT11	MND1		MEF2C		LDHA		TNFAIP8L1		LSR		ldhc	
UIMC1	MPLKIP		MGC24103		LMOD2		TPPP2		LYPLA1		LELP1	
UQCRB	MRPL47		MGC70863		LOC100130428		TRIM42		LYZL1		LINC00238	
ZEB2	MS4A6A		MGC70870		LOC100131541		TSNAXIP1		LYZL2		LINC00301	
ZNF189	MSMO1		MID1		LOC145783		TSSK6		LYZL6		LINC00905	
ZNF22	MTMR1		MIR145		LOC389831		TTLL3		MAPK8IP3		LOC100132319	
ZNF627	MYCL1		MIR15A		LOC402778		TXNDC2		MARCH_11		LOC102724728	
ZNF737	MYO1G		MIR186		LOC595101		UBL4B		MED26		LOC102725072	
	NAA15		MIR199A2		LONRF2		VRK3		MIRN21		LOC145945	
	NAE1		MIR21		LPL		VTRNA1-1		MMEL1		LOC149837	
	NAMPT		MIR27B		LRFN5		VWA5B1		MOGAT2		LOC157740	
	NDUFS4		MIR30E		LRRN2		WDR38		MORN3		LOC196993	
	NOLA1		MIR32		LUM		WFDC9		MROH2B		LOC286187	
	NOLC1		MIR503		LYVE1		WSCD1		MS4A6E		LOC727924	
	NPLOC4		MIR509		LYZ		ZBTB32		MSLN		LOC729461	
	OR10D3		MIR99A		MALAT1		ZDHHC19		MYH9		LOC81691	
	OR10G8		MIRLET7A2		MAMDC2		ZNF474		MYO18B		LRIT1	
	OR8G2		MIRLET7C		MAN2A2		ZNF683		NXPH1		LRRC46	
	OSTC		MIRLET7F1		MDP1		ZNRF4		NYD-SP21		LRWD1	
	OXA1L		MIRLET7G		MEG3				OAZ3		LUZP4	
	PDE8B		MMP15		MEIS1				ODF1		LY86	
	PHF14		MR1		MGC24103				ODF2		LYPD4	
	PIK3CA		MRPL47		MGP				ODF3L1		LYZL6	
	PLDN		MRS2L		MIR145				OR8B8		MAEL	
	PLEKHG4		MS4A4A		MIR199A2				PAPPA2		MAGEA1	
	PNPLA8		MS4A6A		MIR21				PCYT2		MAGEA4	
	PNRC2		MSRB2		MIR27B				PDCD4		MAPK8	
	PP12719		MTMR1		MIR30E				PDYN		MBTD1	
	PRKAA2		MYO5A		MIR32				PDZD8		MME	
	PRKACB		NAV2		MIR99A				PFKP		MORF4L1	
	PRKRIR		NEDD8		MIR99AHG				PGAM2		MROH2B	
	PSMA2		NEUROG3		MIRLET7A2				PGK2		MS4A6E	
	PSMA3		NOLA1		MIRLET7C				PKIB		MTL5	
	PSMC6		NRK		MIRLET7G				PLA2G12B		NCAPH	
	PTPRA		NRP1		MLLT1				PMP2		NEK2	
	PWAR6		OLFML2A		MLXIPL				PODNL1		NF1	
	RALGAPA1		OR2A9P		MME				POM121L2		NFATC4	
	RBX1		OR8G2		MPEG1				PPP3R2		NRP1	
	RDX		OSM		MR1				PRKCDBP		NRXN2	
	RPL15		OXA1L		MS4A7				PRM1		NUP210L	
	RPL21P28		PAGE1		MTMR4				PRM2		NUP62	
	RPL6		PARP14		MTNR1A				PROX1		OAZ3	
	RPS3A		PARP4		MYL7				PRR30		ODF1	
	SC5D		PBEF1		MYLK2				PRSS8		OIP5	
	SCARNA7		PCA3		MYO7A				PTMA		OTX1	
	SCARNA9		PDE5A		NAA10				PTP4A1		PAAF1	
	SDCBP		PDLIM3		NAP1L3				RIMBP3C		PAFAH1B2	
	SDHD		PDZK1		NDUFC1				RNF125		PAGE5	
	SFR1		PELI1		NFIB				RNF151		PAPOLA	
	SGOL1		PFKL		NRP2				ROPN1B		PBK	
	SIX6		PHF14		NTS				RPS6KA1		PCAT4	
	SKA2		PIP5K1C		NUS1				RSHL1		PDHA2	
	SLC37A3		PLA2G4C		OGN				SCN3B		PFN4	
	SMEK3P		PLEK		OMD				SEMG2		PGAM2	
	SMN1		PLLP		OXA1L				SEPTIN12		PGK2	
	SMPD1		PP12719		P2RX7				SIRPD		PGR	
	SNORD113-4		PQLC3		PARP14				SIRPG		PHF14	
	SNORD58A		PRELID1		PCMTD1				SLC11A2		PHF7	
	SNORD73A		PRKCB1		PCSK5				SLC16A7		PIWIL1	
	SNORD78		PRR11		PERP				SLC26A8		PLA2R1	
	SNX2		PRR13		PGCP				SLC2A11		PLK1	
	SPTLC3		PSMD10		PGM2				SLPI		PRKAR2A	

Supplementary Table S4. Up- and Down- regulated Biological Process, Cellular Component, Molecular Function and KEGG Pathways in Post MA, MA, Pre MA and SCOS.

Biological Process of Up-regulated Genes in PostMA

	Term	GO ID	Count
1	lipid biosynthetic process	GO:0008610	10
2	organic hydroxy compound metabolic process	GO:1901615	9
3	alcohol metabolic process	GO:0006066	8
4	small molecule biosynthetic process	GO:0044283	8
5	detection of chemical stimulus	GO:0009593	7
6	detection of chemical stimulus involved in sensory perception	GO:0050907	7
7	organic hydroxy compound biosynthetic process	GO:1901617	7
8	steroid biosynthetic process	GO:0006694	7
9	steroid metabolic process	GO:0008202	7
10	alcohol biosynthetic process	GO:0046165	6
11	cellular component disassembly	GO:0022411	6
12	fatty acid metabolic process	GO:0006631	6
13	monocarboxylic acid metabolic process	GO:0032787	6
14	ATP catabolic process	GO:0006200	5
15	carboxylic acid biosynthetic process	GO:0046394	5
16	cholesterol biosynthetic process	GO:0006695	5
17	cholesterol metabolic process	GO:0008203	5
18	detection of chemical stimulus involved in sensory perception of bitter taste	GO:0001580	5
19	detection of chemical stimulus involved in sensory perception of taste	GO:0050912	5
20	fatty acid biosynthetic process	GO:0006633	5
21	monocarboxylic acid biosynthetic process	GO:0072330	5
22	nucleoside monophosphate catabolic process	GO:0009125	5
23	organic acid biosynthetic process	GO:0016053	5
24	purine nucleoside monophosphate catabolic process	GO:0009128	5
25	purine ribonucleoside monophosphate catabolic process	GO:0009169	5
26	regulation of cellular response to stress	GO:0080135	5
27	ribonucleoside monophosphate catabolic process	GO:0009158	5
28	sterol biosynthetic process	GO:0016126	5
29	sterol metabolic process	GO:0016125	5
30	brain development	GO:0007420	4
31	cellular amide metabolic process	GO:0043603	4
32	peptide metabolic process	GO:0006518	4
33	positive regulation of stress-activated MAPK cascade	GO:0032874	4
34	positive regulation of stress-activated protein kinase signaling cascade	GO:0070304	4
35	regulation of stress-activated MAPK cascade	GO:0032872	4
36	regulation of stress-activated protein kinase signaling cascade	GO:0070302	4
37	cellular protein complex disassembly	GO:0043624	3
38	cotranslational protein targeting to membrane	GO:0006613	3
39	establishment of protein localization to endoplasmic reticulum	GO:0072599	3
40	glutathione metabolic process	GO:0006749	3
41	isoprenoid metabolic process	GO:0006720	3
42	macromolecular complex disassembly	GO:0032984	3
43	monocarboxylic acid transport	GO:0015718	3
44	nuclear-transcribed mRNA catabolic process, nonsense-mediated decay	GO:0000184	3
45	positive regulation of carbohydrate metabolic process	GO:0045913	3
46	positive regulation of cellular carbohydrate metabolic process	GO:0010676	3
47	positive regulation of JNK cascade	GO:0046330	3
48	protein complex disassembly	GO:0043241	3
49	protein localization to endoplasmic reticulum	GO:0070972	3
50	protein targeting to ER	GO:0045047	3
51	protein targeting to membrane	GO:0006612	3
52	regulation of cellular carbohydrate metabolic process	GO:0010675	3
53	regulation of JNK cascade	GO:0046328	3
54	sensory perception of chemical stimulus	GO:0007606	3
55	SRP-dependent cotranslational protein targeting to membrane	GO:0006614	3
56	translational elongation	GO:0006414	3
57	translational initiation	GO:0006413	3
58	translational termination	GO:0006415	3
59	viral life cycle	GO:0019058	3
60	viral transcription	GO:0019083	3
61	androgen biosynthetic process	GO:0006702	2
62	androgen metabolic process	GO:0008209	2
63	cellular response to gonadotropin stimulus	GO:0071371	2
64	fatty acid transport	GO:0015908	2
65	genitalia development	GO:0048806	2
66	glutathione derivative biosynthetic process	GO:1901687	2
67	glutathione derivative metabolic process	GO:1901685	2
68	hormone biosynthetic process	GO:0042446	2
69	isoprenoid biosynthetic process	GO:0008299	2
70	long-chain fatty acid transport	GO:0015909	2
71	male genitalia development	GO:0030539	2
72	negative regulation of blood circulation	GO:1903523	2
73	positive regulation of alcohol biosynthetic process	GO:1902932	2
74	positive regulation of inositol phosphate biosynthetic process	GO:0060732	2
75	regulation of alcohol biosynthetic process	GO:1902930	2
76	regulation of inositol phosphate biosynthetic process	GO:0010919	2
77	reproductive system development	GO:0061458	2
78	response to cholesterol	GO:0070723	2
79	response to gonadotropin	GO:0034698	2
80	response to sterol	GO:0036314	2
81	sensory perception of taste	GO:0050909	2
82	substrate adhesion-dependent cell spreading	GO:0034446	2

Biological Process of Up-regulated Genes in MA

	Term	GO ID	Count
1	lipid biosynthetic process	GO:0008610	10
2	negative regulation of cell cycle	GO:0045786	8
3	regulation of cell cycle process	GO:0010564	8
4	regulation of mitotic cell cycle	GO:0007346	8
5	mitotic cell cycle	GO:0000278	7
6	nuclear division	GO:0000280	7
7	organelle fission	GO:0048285	7
8	regulation of cell cycle phase transition	GO:1901987	7
9	regulation of mitotic cell cycle phase transition	GO:1901990	7
10	chromosome segregation	GO:0007059	6
11	mitotic nuclear division	GO:0007067	6
12	mRNA processing	GO:0006397	6
13	negative regulation of cell cycle phase transition	GO:1901988	6
14	negative regulation of cell cycle process	GO:0010948	6
15	negative regulation of mitotic cell cycle	GO:0045930	6
16	negative regulation of mitotic cell cycle phase transition	GO:1901991	6
17	alcohol metabolic process	GO:0006066	5
18	carboxylic acid biosynthetic process	GO:0046394	5
19	cell cycle checkpoint	GO:0000075	5
20	DNA integrity checkpoint	GO:0031570	5
21	fatty acid biosynthetic process	GO:0006633	5
22	fatty acid metabolic process	GO:0006631	5
23	glycerolipid metabolic process	GO:0046486	5
24	monocarboxylic acid biosynthetic process	GO:0072330	5
25	mRNA catabolic process	GO:0006402	5
26	nuclear-transcribed mRNA catabolic process	GO:0000956	5
27	organic acid biosynthetic process	GO:0016053	5
28	positive regulation of cell cycle	GO:0045787	5
29	positive regulation of cell cycle process	GO:0090068	5
30	protein folding	GO:0006457	5
31	response to peptide hormone	GO:0043434	5
32	RNA catabolic process	GO:0006401	5
33	chromosome organization	GO:0051276	4
34	glycerolipid biosynthetic process	GO:0045017	4
35	glycerophospholipid biosynthetic process	GO:0046474	4
36	glycerophospholipid metabolic process	GO:0006650	4
37	lipid transport	GO:0006869	4
38	meiotic cell cycle process	GO:1903046	4
39	mitotic cell cycle checkpoint	GO:0007093	4
40	negative regulation of intracellular transport	GO:0032387	4
41	phospholipid biosynthetic process	GO:0008654	4
42	regulation of cell cycle G1/S phase transition	GO:1902806	4
43	regulation of cell division	GO:0051302	4
44	regulation of cellular ketone metabolic process	GO:0010565	4
45	regulation of G1/S transition of mitotic cell cycle	GO:2000045	4
46	regulation of mitosis	GO:0007088	4
47	regulation of nuclear division	GO:0051783	4
48	steroid biosynthetic process	GO:0006694	4
49	steroid metabolic process	GO:0008202	4
50	alcohol biosynthetic process	GO:0046165	3
51	cholesterol biosynthetic process	GO:0006695	3
52	cholesterol metabolic process	GO:0008203	3
53	'de novo' posttranslational protein folding	GO:0051084	3
54	'de novo' protein folding	GO:0006458	3
55	DNA damage checkpoint	GO:0000077	3
56	DNA damage response, signal transduction by p53 class mediator	GO:0030330	3
57	DNA damage response, signal transduction by p53 class mediator resulting in cell cycle arrest	GO:0006977	3
58	double-strand break repair	GO:0006302	3
59	fatty acid transport	GO:0015908	3
60	intracellular signal transduction involved in G1 DNA damage checkpoint	GO:1902400	3
61	long-chain fatty acid transport	GO:0015909	3
62	mitotic DNA integrity checkpoint	GO:0044774	3
63	monocarboxylic acid transport	GO:0015718	3
64	negative regulation of cell cycle G1/S phase transition	GO:1902807	3
65	negative regulation of G1/S transition of mitotic cell cycle	GO:2000134	3
66	nuclear-transcribed mRNA catabolic process, nonsense-mediated decay	GO:0000184	3
67	nucleosome organization	GO:0034728	3
68	phosphatidylinositol biosynthetic process	GO:0006661	3
69	phosphatidylinositol metabolic process	GO:0046488	3
70	positive regulation of cell cycle arrest	GO:0071158	3
71	positive regulation of mitotic cell cycle	GO:0045931	3
72	protein-DNA complex subunit organization	GO:0071824	3
73	regulation of cell cycle arrest	GO:0071156	3
74	response to ionizing radiation	GO:0010212	3
75	ribonucleoprotein complex assembly	GO:0022618	3
76	rRNA metabolic process	GO:0016072	3
77	rRNA processing	GO:0006364	3
78	signal transduction by p53 class mediator	GO:0072331	3
79	signal transduction in response to DNA damage	GO:0042770	3
80	signal transduction involved in cell cycle checkpoint	GO:0072395	3
81	signal transduction involved in DNA damage checkpoint	GO:0072422	3
82	signal transduction involved in DNA integrity checkpoint	GO:0072401	3
83	signal transduction involved in mitotic cell cycle checkpoint	GO:0072413	3
84	signal transduction involved in mitotic DNA damage checkpoint	GO:1902402	3
85	signal transduction involved in mitotic DNA integrity checkpoint	GO:1902403	3
86	signal transduction involved in mitotic G1 DNA damage checkpoint	GO:0072431	3
87	sterol biosynthetic process	GO:0016126	3
88	sterol metabolic process	GO:0016125	3
89	translational initiation	GO:0006413	3
90	acid secretion	GO:0046717	2
91	arachidonic acid secretion	GO:0050482	2
92	detection of chemical stimulus involved in sensory perception of bitter taste	GO:0001580	2
93	detection of chemical stimulus involved in sensory perception of taste	GO:0050912	2
94	exonucleolytic nuclear-transcribed mRNA catabolic process involved in deadenylation-dependent decay	GO:0043928	2
95	fatty acid derivative transport	GO:1901571	2
96	G2 DNA damage checkpoint	GO:0031572	2
97	glutathione derivative biosynthetic process	GO:1901687	2
98	glutathione derivative metabolic process	GO:1901685	2
99	glutathione metabolic process	GO:0006749	2
100	glycerolipid catabolic process	GO:0046503	2
101	Golgi to plasma membrane protein transport	GO:0043001	2
102	Golgi to plasma membrane transport	GO:0006893	2
103	histone phosphorylation	GO:0016572	2
104	histone-serine phosphorylation	GO:0035404	2
105	icosanoid secretion	GO:0032309	2
106	icosanoid transport	GO:0071715	2
107	meiotic chromosome segregation	GO:0045132	2
108	mitotic G2/M transition checkpoint	GO:0044818	2
109	mitotic spindle assembly checkpoint	GO:0007094	2
110	mitotic spindle checkpoint	GO:0071174	2
111	morphogenesis of an epithelial sheet	GO:0002011	2
112	negative regulation of cell cycle G2/M phase transition	GO:1902750	2
113	negative regulation of chromosome segregation	GO:0051985	2
114	negative regulation of extrinsic apoptotic signaling pathway in absence of ligand	GO:2001240	2
115	negative regulation of G2/M transition of mitotic cell cycle	GO:0010972	2
116	negative regulation of lipid biosynthetic process	GO:0051055	2
117	negative regulation of metaphase/anaphase transition of cell cycle	GO:1902100	2
118	negative regulation of mitosis	GO:0045839	2
119	negative regulation of mitotic metaphase/anaphase transition	GO:0045841	2
120	negative regulation of mitotic sister chromatid segregation	GO:0033048	2
121	negative regulation of mitotic sister chromatid separation	GO:2000816	2
122	negative regulation of oxidoreductase activity	GO:0051354	2
123	negative regulation of signal transduction in absence of ligand	GO:1901099	2
124	negative regulation of sister chromatid segregation	GO:0033046	2
125	negative regulation of stem cell proliferation	GO:2000647	2
126	negative regulation of steroid biosynthetic process	GO:0010894	2
127	negative regulation of steroid metabolic process	GO:0045939	2
128	neuron apoptotic process	GO:0051402	2
129	neuron death	GO:0070997	2
130	nuclear-transcribed mRNA catabolic process, exonucleolytic	GO:0000291	2
131	organelle fusion	GO:0048284	2
132	phosphatidylinositol-3-phosphate biosynthetic process	GO:0036092	2
133	positive regulation of axon extension	GO:0045773	2
134	protein depolymerization	GO:0051261	2
135	protein neddylation	GO:0045116	2
136	reciprocal DNA recombination	GO:0035825	2
137	reciprocal meiotic recombination	GO:0007131	2
138	regulation of cell cycle G2/M phase transition	GO:1902749	2
139	regulation of fatty acid biosynthetic process	GO:0042304	2
140	regulation of G2/M transition of mitotic cell cycle	GO:0010389	2
141	regulation of metaphase/anaphase transition of cell cycle	GO:1902099	2
142	regulation of mitotic metaphase/anaphase transition	GO:0030071	2
143	regulation of mitotic sister chromatid segregation	GO:0033047	2
144	regulation of mitotic sister chromatid separation	GO:0010965	2
145	regulation of sister chromatid segregation	GO:0033045	2
146	regulation of TOR signaling	GO:0032006	2
147	resolution of meiotic recombination intermediates	GO:0000712	2
148	segmentation	GO:0035282	2
149	somitogenesis	GO:0001756	2
150	spindle assembly checkpoint	GO:0071173	2
151	spindle checkpoint	GO:0031577	2
152	vesicle fusion	GO:0006906	2

Biological Process of Up-regulated Genes in SCOC

	Term	GO ID	Count
1	activation of immune response	GO:0002253	10
2	regulation of cytokine production	GO:0001817	10
3	antigen processing and presentation	GO:0019882	9
4	antigen processing and presentation of peptide antigen	GO:0048002	9
5	cellular response to cytokine stimulus	GO:0071345	9
6	aminoglycan metabolic process	GO:0006022	8
7	blood coagulation	GO:0007596	8
8	coagulation	GO:0050817	8
9	cytokine-mediated signaling pathway	GO:0019221	8
10	hemostasis	GO:0007599	8
11	immune response-activating signal transduction	GO:0002757	8
12	monocarboxylic acid metabolic process	GO:0032787	8
13	positive regulation of kinase activity	GO:0033674	8
14	positive regulation of MAPK cascade	GO:0043410	8
15	positive regulation of protein kinase activity	GO:0045860	8
16	regulation of cell activation	GO:0050865	8
17	regulation of leukocyte activation	GO:0002694	8
18	activation of protein kinase activity	GO:0032147	7
19	antigen processing and presentation of exogenous antigen	GO:0019884	7
20	antigen processing and presentation of exogenous peptide antigen	GO:0002478	7
21	cellular response to nitrogen compound	GO:1901699	7
22	cellular response to organonitrogen compound	GO:0071417	7
23	endocytosis	GO:0006897	7
24	glycosaminoglycan metabolic process	GO:0030203	7
25	negative regulation of protein modification process	GO:0031400	7
26	negative regulation of transport	GO:0051051	7
27	positive regulation of cytokine production	GO:0001819	7
28	regulation of establishment of protein localization	GO:0070201	7
29	regulation of immune effector process	GO:0002697	7
30	regulation of intracellular transport	GO:0032386	7
31	regulation of lymphocyte activation	GO:0051249	7
32	regulation of response to wounding	GO:1903034	7
33	response to drug	GO:0042493	7
34	response to inorganic substance	GO:0010035	7
35	sulfur compound metabolic process	GO:0006790	7
36	synaptic transmission	GO:0007268	7
37	aminoglycan catabolic process	GO:0006026	6
38	axon guidance	GO:0007411	6
39	carbohydrate derivative metabolic process	GO:1901137	6
40	cellular response to interferon-gamma	GO:0071346	6
41	generation of precursor metabolites and energy	GO:0006091	6
42	growth	GO:0040007	6
43	interferon-gamma-mediated signaling pathway	GO:0060333	6
44	mucopolysaccharide metabolic process	GO:1903510	6
45	neuron projection guidance	GO:0097485	6
46	neurotrophin signaling pathway	GO:0038179	6
47	neurotrophin TRK receptor signaling pathway	GO:0048011	6
48	positive regulation of secretion	GO:0051047	6
49	regulation of cytoskeleton organization	GO:0051493	6
50	regulation of protein transport	GO:0051223	6
51	regulation of T cell activation	GO:0050863	6
52	response to interferon-gamma	GO:0034341	6
53	aminoglycan biosynthetic process	GO:0006023	5
54	anatomical structure homeostasis	GO:0060249	5
55	antigen processing and presentation of peptide antigen via MHC class I	GO:0002474	5
56	carbohydrate homeostasis	GO:0033500	5
57	carboxylic acid biosynthetic process	GO:0046394	5
58	chemotaxis	GO:0006935	5
59	defense response to other organism	GO:0098542	5
60	epithelial cell differentiation	GO:0030855	5
61	glucose homeostasis	GO:0042593	5
62	glycosaminoglycan biosynthetic process	GO:0006024	5
63	glycosaminoglycan catabolic process	GO:0006027	5
64	hexose metabolic process	GO:0019318	5
65	immune response-activating cell surface receptor signaling pathway	GO:0002429	5
66	leukocyte migration	GO:0050900	5
67	lipid catabolic process	GO:0016042	5
68	monosaccharide metabolic process	GO:0005996	5
69	muscle contraction	GO:0006936	5
70	muscle system process	GO:0003012	5
71	negative regulation of cytokine production	GO:0001818	5
72	negative regulation of multi-organism process	GO:0043901	5
73	negative regulation of protein phosphorylation	GO:0001933	5
74	organic acid biosynthetic process	GO:0016053	5
75	platelet activation	GO:0030168	5
76	positive regulation of cell activation	GO:0050867	5
77	positive regulation of leukocyte activation	GO:0002696	5
78	positive regulation of locomotion	GO:0040017	5
79	positive regulation of lymphocyte activation	GO:0051251	5
80	positive regulation of secretion by cell	GO:1903532	5
81	positive regulation of sequence-specific DNA binding transcription factor activity	GO:0051091	5
82	positive regulation of T cell activation	GO:0050870	5
83	protein maturation	GO:0051604	5
84	protein processing	GO:0016485	5
85	receptor-mediated endocytosis	GO:0006898	5
86	regulation of cell morphogenesis involved in differentiation	GO:0010769	5
87	regulation of extrinsic apoptotic signaling pathway	GO:2001236	5
88	regulation of hormone levels	GO:0010817	5
89	regulation of inflammatory response	GO:0050727	5
90	regulation of leukocyte proliferation	GO:0070663	5
91	regulation of lymphocyte proliferation	GO:0050670	5
92	regulation of mononuclear cell proliferation	GO:0032944	5
93	regulation of multi-organism process	GO:0043900	5
94	regulation of symbiosis, encompassing mutualism through parasitism	GO:0043903	5
95	response to metal ion	GO:0010038	5
96	response to oxidative stress	GO:0006979	5
97	synapse organization	GO:0050808	5
98	taxis	GO:0042330	5
99	activation of MAPKK activity	GO:0000186	4
100	antigen processing and presentation of exogenous peptide antigen via MHC class II	GO:0019886	4
101	antigen processing and presentation of peptide or polysaccha	GO:0002504	4
102	antigen receptor-mediated signaling pathway	GO:0050851	4
103	cellular amide metabolic process	GO:0043603	4
104	coenzyme metabolic process	GO:0006732	4
105	developmental growth	GO:0048589	4
106	energy derivation by oxidation of organic compounds	GO:0015980	4
107	glucose metabolic process	GO:0006006	4
108	hormone metabolic process	GO:0042445	4
109	keratan sulfate catabolic process	GO:0042340	4
110	keratan sulfate metabolic process	GO:0042339	4
111	lymphocyte costimulation	GO:0031294	4
112	monocarboxylic acid biosynthetic process	GO:0072330	4
113	negative regulation of defense response	GO:0031348	4
114	negative regulation of response to external stimulus	GO:0032102	4
115	negative regulation of secretion	GO:0051048	4
116	negative regulation of secretion by cell	GO:1903531	4
117	neuron migration	GO:0001764	4
118	organic hydroxy compound biosynthetic process	GO:1901617	4
119	ossification	GO:0001503	4
120	positive regulation of I-kappaB kinase/NF-kappaB signaling	GO:0043123	4
121	positive regulation of ion transport	GO:0043270	4
122	regulation of behavior	GO:0050795	4
123	regulation of calcium ion transport	GO:0051924	4
124	regulation of chemotaxis	GO:0050920	4
125	regulation of cysteine-type endopeptidase activity	GO:2000116	4
126	regulation of cysteine-type endopeptidase activity involved in apoptotic process	GO:0043281	4
127	regulation of cytokine secretion	GO:0050707	4
128	regulation of I-kappaB kinase/NF-kappaB signaling	GO:0043122	4
129	regulation of interleukin-1 production	GO:0032652	4
130	regulation of microtubule cytoskeleton organization	GO:0070507	4
131	regulation of microtubule-based process	GO:0032886	4
132	regulation of nucleocytoplasmic transport	GO:0046822	4
133	regulation of ossification	GO:0030278	4
134	regulation of protein secretion	GO:0050708	4
135	regulation of T cell proliferation	GO:0042129	4
136	regulation of type I interferon production	GO:0032479	4
137	regulation of viral process	GO:0050792	4
138	response to organophosphorus	GO:0046683	4
139	striated muscle contraction	GO:0006941	4
140	sulfur compound biosynthetic process	GO:0044272	4
141	sulfur compound catabolic process	GO:0044273	4
142	T cell costimulation	GO:0031295	4
143	T cell receptor signaling pathway	GO:0050852	4
144	tissue homeostasis	GO:0001894	4
145	xenobiotic metabolic process	GO:0006805	4
146	alpha-amino acid catabolic process	GO:1901606	3
147	antigen processing and presentation of exogenous peptide antigen via MHC class I	GO:0042590	3
148	antigen processing and presentation of exogenous peptide antigen via MHC class I, TAP-dependent	GO:0002479	3
149	antigen processing and presentation of peptide antigen via MHC class II	GO:0002495	3
150	behavioral response to cocaine	GO:0048148	3
151	cell recognition	GO:0008037	3
152	cell-matrix adhesion	GO:0007160	3
153	endosome organization	GO:0007032	3
154	establishment of synaptic vesicle localization	GO:0097480	3
155	establishment of vesicle localization	GO:0051650	3
156	fatty acid transport	GO:0015908	3
157	keratan sulfate biosynthetic process	GO:0018146	3
158	long-chain fatty acid transport	GO:0015909	3
159	monocarboxylic acid transport	GO:0015718	3
160	myeloid leukocyte migration	GO:0097529	3
161	negative regulation of axonogenesis	GO:0050771	3
162	negative regulation of binding	GO:0051100	3
163	negative regulation of cell morphogenesis involved in differentiation	GO:0010771	3
164	negative regulation of cysteine-type endopeptidase activity	GO:2000117	3
165	negative regulation of cysteine-type endopeptidase activity involved in apoptotic process	GO:0043154	3
166	negative regulation of extrinsic apoptotic signaling pathway	GO:2001237	3
167	negative regulation of intracellular transport	GO:0032387	3
168	negative regulation of neuron projection development	GO:0010977	3
169	negative regulation of viral process	GO:0048525	3
170	neutral lipid metabolic process	GO:0006638	3
171	peptide metabolic process	GO:0006518	3
172	phenol-containing compound metabolic process	GO:0018958	3
173	positive regulation of behavior	GO:0048520	3
174	positive regulation of calcium ion transport	GO:0051928	3
175	positive regulation of chemotaxis	GO:0050921	3
176	positive regulation of cytokine secretion	GO:0050715	3
177	positive regulation of endocytosis	GO:0045807	3
178	positive regulation of glucose import	GO:0046326	3
179	positive regulation of glucose transport	GO:0010828	3
180	positive regulation of leukocyte proliferation	GO:0070665	3
181	positive regulation of lymphocyte proliferation	GO:0050671	3
182	positive regulation of mononuclear cell proliferation	GO:0032946	3
183	positive regulation of ossification	GO:0045778	3
184	positive regulation of osteoblast differentiation	GO:0045669	3
185	positive regulation of protein secretion	GO:0050714	3
186	positive regulation of stress-activated MAPK cascade	GO:0032874	3
187	positive regulation of stress-activated protein kinase signaling cascade	GO:0070304	3
188	positive regulation of T cell proliferation	GO:0042102	3
189	post-embryonic development	GO:0009791	3
190	reactive oxygen species metabolic process	GO:0072593	3
191	regeneration	GO:0031099	3
192	regulation of acute inflammatory response	GO:0002673	3
193	regulation of blood coagulation	GO:0030193	3
194	regulation of cellular response to oxidative stress	GO:1900407	3
195	regulation of coagulation	GO:0050818	3
196	regulation of defense response to virus	GO:0050688	3
197	regulation of DNA binding	GO:0051101	3
198	regulation of DNA replication	GO:0006275	3
199	regulation of glucose import	GO:0046324	3
200	regulation of glucose transport	GO:0010827	3
201	regulation of hemostasis	GO:1900046	3
202	regulation of interleukin-1 beta production	GO:0032651	3
203	regulation of interleukin-1 secretion	GO:0050704	3
204	regulation of interleukin-6 production	GO:0032675	3
205	regulation of osteoblast differentiation	GO:0045667	3
206	regulation of peptidyl-serine phosphorylation	GO:0033135	3
207	regulation of reactive oxygen species metabolic process	GO:2000377	3
208	regulation of response to biotic stimulus	GO:0002831	3
209	regulation of response to oxidative stress	GO:1902882	3
210	regulation of smooth muscle cell proliferation	GO:0048660	3
211	regulation of viral genome replication	GO:0045069	3
212	regulation of wound healing	GO:0061041	3
213	response to amino acid	GO:0043200	3
214	response to ammonium ion	GO:0060359	3
215	response to cocaine	GO:0042220	3
216	response to interleukin-1	GO:0070555	3
217	semaphorin-plexin signaling pathway	GO:0071526	3
218	synaptic vesicle transport	GO:0048489	3
219	7-methylguanosine mRNA capping	GO:0006370	2
220	7-methylguanosine RNA capping	GO:0009452	2
221	adherens junction assembly	GO:0034333	2
222	adhesion of symbiont to host	GO:0044406	2
223	adhesion of symbiont to host cell	GO:0044650	2
224	alditol phosphate metabolic process	GO:0052646	2
225	angiotensin maturation	GO:0002003	2
226	apoptotic cell clearance	GO:0043277	2
227	apoptotic process involved in development	GO:1902742	2
228	arachidonic acid secretion	GO:0050482	2
229	axon extension	GO:0048675	2
230	calcium ion import	GO:0070509	2
231	catecholamine biosynthetic process	GO:0042423	2
232	catechol-containing compound biosynthetic process	GO:0009713	2
233	cellular response to glucose stimulus	GO:0071333	2
234	cellular response to hexose stimulus	GO:0071331	2
235	central nervous system neuron development	GO:0021954	2
236	chemical homeostasis within a tissue	GO:0048875	2
237	collagen fibril organization	GO:0030199	2
238	detection of chemical stimulus involved in sensory perception of bitter taste	GO:0001580	2
239	detection of chemical stimulus involved in sensory perception of taste	GO:0050912	2
240	dopamine biosynthetic process	GO:0042416	2
241	dopamine metabolic process	GO:0042417	2
242	embryonic digestive tract development	GO:0048566	2
243	ephrin receptor signaling pathway	GO:0048013	2
244	extracellular matrix assembly	GO:0085029	2
245	fatty acid derivative transport	GO:1901571	2
246	G2 DNA damage checkpoint	GO:0031572	2
247	genetic imprinting	GO:0071514	2
248	glucan biosynthetic process	GO:0009250	2
249	glutathione derivative biosynthetic process	GO:1901687	2
250	glutathione derivative metabolic process	GO:1901685	2
251	glycogen biosynthetic process	GO:0005978	2
252	hair cell differentiation	GO:0035315	2
253	hyaluronan catabolic process	GO:0030214	2
254	hyaluronan metabolic process	GO:0030212	2
255	hydrogen peroxide catabolic process	GO:0042744	2
256	hydrogen peroxide metabolic process	GO:0042743	2
257	icosanoid secretion	GO:0032309	2
258	icosanoid transport	GO:0071715	2
259	lamellipodium assembly	GO:0030032	2
260	lamellipodium organization	GO:0097581	2
261	layer formation in cerebral cortex	GO:0021819	2
262	lipid storage	GO:0019915	2
263	long-term memory	GO:0007616	2
264	long-term synaptic potentiation	GO:0060291	2
265	lung cell differentiation	GO:0060479	2
266	lung epithelial cell differentiation	GO:0060487	2
267	lung secretory cell differentiation	GO:0061140	2
268	motor neuron axon guidance	GO:0008045	2
269	muscle adaptation	GO:0043500	2
270	myotube cell development	GO:0014904	2
271	negative chemotaxis	GO:0050919	2
272	negative regulation of amine transport	GO:0051953	2
273	negative regulation of androgen receptor signaling pathway	GO:0060766	2
274	negative regulation of axon extension	GO:0030517	2
275	negative regulation of chemotaxis	GO:0050922	2
276	negative regulation of defense response to virus	GO:0050687	2
277	negative regulation of DNA binding	GO:0043392	2
278	negative regulation of ERK1 and ERK2 cascade	GO:0070373	2
279	negative regulation of insulin secretion	GO:0046676	2
280	negative regulation of intracellular steroid hormone receptor signaling pathway	GO:0033144	2
281	negative regulation of lipid transport	GO:0032369	2
282	negative regulation of nucleoside metabolic process	GO:0045978	2
283	negative regulation of peptide hormone secretion	GO:0090278	2
284	negative regulation of peptide secretion	GO:0002792	2
285	negative regulation of response to biotic stimulus	GO:0002832	2
286	negative regulation of viral genome replication	GO:0045071	2
287	neuromuscular synaptic transmission	GO:0007274	2
288	neuron projection extension	GO:1990138	2
289	oligosaccharide catabolic process	GO:0009313	2
290	oligosaccharide metabolic process	GO:0009311	2
291	peptide cross-linking	GO:0018149	2
292	peptide hormone processing	GO:0016486	2
293	phenol-containing compound biosynthetic process	GO:0046189	2
294	phosphatidic acid biosynthetic process	GO:0006654	2
295	phosphatidic acid metabolic process	GO:0046473	2
296	positive regulation of blood coagulation	GO:0030194	2
297	positive regulation of calcium ion transport into cytosol	GO:0010524	2
298	positive regulation of cell killing	GO:0031343	2
299	positive regulation of cellular response to insulin stimulus	GO:1900078	2
300	positive regulation of coagulation	GO:0050820	2
301	positive regulation of fat cell differentiation	GO:0045600	2
302	positive regulation of hemostasis	GO:1900048	2
303	positive regulation of interleukin-1 beta production	GO:0032731	2
304	positive regulation of interleukin-1 beta secretion	GO:0050718	2
305	positive regulation of interleukin-1 production	GO:0032732	2
306	positive regulation of interleukin-1 secretion	GO:0050716	2
307	positive regulation of leukocyte mediated cytotoxicity	GO:0001912	2
308	positive regulation of phagocytosis	GO:0050766	2
309	positive regulation of release of sequestered calcium ion into cytosol	GO:0051281	2
310	positive regulation of T cell mediated cytotoxicity	GO:0001916	2
311	positive regulation of T cell mediated immunity	GO:0002711	2
312	positive regulation of tumor necrosis factor production	GO:0032760	2
313	positive regulation of vasoconstriction	GO:0045907	2
314	prostate gland growth	GO:0060736	2
315	protein K48-linked ubiquitination	GO:0070936	2
316	protein kinase B signaling	GO:0043491	2
317	protein localization to synapse	GO:0035418	2
318	regulation of androgen receptor signaling pathway	GO:0060765	2
319	regulation of calcium ion import	GO:0090279	2
320	regulation of calcium-mediated signaling	GO:0050848	2
321	regulation of catecholamine secretion	GO:0050433	2
322	regulation of complement activation	GO:0030449	2
323	regulation of defense response to virus by virus	GO:0050690	2
324	regulation of DNA biosynthetic process	GO:2000278	2
325	regulation of DNA-dependent DNA replication	GO:0090329	2
326	regulation of dopamine secretion	GO:0014059	2
327	regulation of endocrine process	GO:0044060	2
328	regulation of gene expression by genetic imprinting	GO:0006349	2
329	regulation of glomerular filtration	GO:0003093	2
330	regulation of glucose import in response to insulin stimulus	GO:2001273	2
331	regulation of interleukin-1 beta secretion	GO:0050706	2
332	regulation of leukocyte mediated cytotoxicity	GO:0001910	2
333	regulation of microtubule polymerization	GO:0031113	2
334	regulation of protein activation cascade	GO:2000257	2
335	regulation of protein export from nucleus	GO:0046825	2
336	regulation of release of cytochrome c from mitochondria	GO:0090199	2
337	regulation of response to reactive oxygen species	GO:1901031	2
338	regulation of sulfur metabolic process	GO:0042762	2
339	regulation of superoxide metabolic process	GO:0090322	2
340	regulation of T cell mediated cytotoxicity	GO:0001914	2
341	regulation of viral entry into host cell	GO:0046596	2
342	renal system process involved in regulation of blood volume	GO:0001977	2
343	renal system process involved in regulation of systemic arterial blood pressure	GO:0003071	2
344	response to cold	GO:0009409	2
345	response to interferon-alpha	GO:0035455	2
346	response to X-ray	GO:0010165	2
347	retina homeostasis	GO:0001895	2
348	RNA capping	GO:0036260	2
349	serine family amino acid catabolic process	GO:0009071	2
350	serine family amino acid metabolic process	GO:0009069	2
351	skeletal muscle adaptation	GO:0043501	2
352	skeletal muscle fiber development	GO:0048741	2
353	striated muscle adaptation	GO:0014888	2
354	synaptic transmission, dopaminergic	GO:0001963	2
355	synaptic vesicle endocytosis	GO:0048488	2
356	T cell differentiation in thymus	GO:0033077	2
357	T cell mediated immunity	GO:0002456	2
358	viral genome replication	GO:0019079	2
359	virion attachment to host cell	GO:0019062	2
360	xenobiotic catabolic process	GO:0042178	2

Biological Process of Down-regulated Genes in PostMA

	Term	GO ID	Count
1	multicellular organismal reproductive process	GO:0048609	14
2	gamete generation	GO:0007276	13
3	male gamete generation	GO:0048232	13
4	spermatogenesis	GO:0007283	13
5	multicellular organismal development	GO:0007275	8
6	cellular process involved in reproduction in multicellular organism	GO:0022412	6
7	microtubule-based process	GO:0007017	6
8	negative regulation of intracellular signal transduction	GO:1902532	5
9	regulation of membrane potential	GO:0042391	5
10	single-organism behavior	GO:0044708	5
11	cell recognition	GO:0008037	4
12	fertilization	GO:0009566	4
13	microtubule cytoskeleton organization	GO:0000226	4
14	microtubule-based movement	GO:0007018	4
15	ncRNA processing	GO:0034470	4
16	regulation of synaptic transmission	GO:0050804	4
17	response to alcohol	GO:0097305	4
18	sperm motility	GO:0030317	4
19	adult behavior	GO:0030534	3
20	cell maturation	GO:0048469	3
21	cell-cell recognition	GO:0009988	3
22	hydrogen transport	GO:0006818	3
23	membrane depolarization	GO:0051899	3
24	multi-multicellular organism process	GO:0044706	3
25	multi-organism reproductive process	GO:0044703	3
26	negative regulation of MAPK cascade	GO:0043409	3
27	proton transport	GO:0015992	3
28	regulation of exocytosis	GO:0017157	3
29	regulation of signal transduction by p53 class mediator	GO:1901796	3
30	response to calcium ion	GO:0051592	3
31	response to ketone	GO:1901654	3
32	response to retinoic acid	GO:0032526	3
33	response to temperature stimulus	GO:0009266	3
34	rRNA metabolic process	GO:0016072	3
35	rRNA processing	GO:0006364	3
36	sperm-egg recognition	GO:0035036	3
37	cell wall macromolecule catabolic process	GO:0016998	2
38	cell wall macromolecule metabolic process	GO:0044036	2
39	cellular response to heat	GO:0034605	2
40	cilium or flagellum-dependent cell motility	GO:0001539	2
41	DNA damage response, signal transduction by p53 class mediator resulting in transcription of p21 class mediator	GO:0006978	2
42	DNA damage response, signal transduction resulting in transcription	GO:0042772	2
43	multicellular organism reproduction	GO:0032504	2
44	phospholipid transport	GO:0015914	2
45	positive regulation of macrophage activation	GO:0043032	2
46	regulation of fatty acid transport	GO:2000191	2
47	regulation of macrophage activation	GO:0043030	2
48	regulation of organic acid transport	GO:0032890	2
49	regulation of transforming growth factor beta production	GO:0071634	2
50	reproduction	GO:0000003	2
51	response to progesterone	GO:0032570	2
52	response to testosterone	GO:0033574	2
53	spermatid development	GO:0007286	2

Biological Process of Down-regulated Genes in MA

	Term	GO ID	Count
1	multicellular organismal reproductive process	GO:0048609	19
2	gamete generation	GO:0007276	18
3	male gamete generation	GO:0048232	18
4	spermatogenesis	GO:0007283	18
5	multicellular organismal development	GO:0007275	13
6	cellular process involved in reproduction in multicellular organism	GO:0022412	5
7	germ cell development	GO:0007281	5
8	spermatid development	GO:0007286	5
9	sperm motility	GO:0030317	4
10	cell wall macromolecule catabolic process	GO:0016998	3
11	cell wall macromolecule metabolic process	GO:0044036	3
12	DNA conformation change	GO:0071103	3
13	DNA packaging	GO:0006323	3
14	fertilization	GO:0009566	3
15	peptidyl-tyrosine dephosphorylation	GO:0035335	3
16	acrosome reaction	GO:0007340	2
17	calcium ion-dependent exocytosis	GO:0017156	2
18	chromosome condensation	GO:0030261	2
19	positive regulation of lyase activity	GO:0051349	2
20	positive regulation of striated muscle cell differentiation	GO:0051155	2
21	regulation of cell cycle checkpoint	GO:1901976	2
22	regulation of lyase activity	GO:0051339	2
23	reproduction	GO:0000003	2
24	sperm-egg recognition	GO:0035036	2

Biological Process of Down-regulated Genes in SCOS

	Term	GO ID	Count
1	multicellular organismal reproductive process	GO:0048609	17
2	gamete generation	GO:0007276	16
3	male gamete generation	GO:0048232	16
4	multicellular organismal development	GO:0007275	16
5	spermatogenesis	GO:0007283	16
6	nuclear division	GO:0000280	14
7	organelle fission	GO:0048285	14
8	cellular process involved in reproduction in multicellular organism	GO:0022412	11
9	microtubule-based process	GO:0007017	10
10	mitotic nuclear division	GO:0007067	10
11	sperm motility	GO:0030317	9
12	chromosome organization	GO:0051276	8
13	germ cell development	GO:0007281	8
14	spermatid development	GO:0007286	8
15	mitotic cell cycle	GO:0000278	7
16	cell cycle phase transition	GO:0044770	6
17	microtubule cytoskeleton organization	GO:0000226	6
18	mitotic cell cycle phase transition	GO:0044772	6
19	response to light stimulus	GO:0009416	6
20	brain development	GO:0007420	5
21	carbohydrate catabolic process	GO:0016052	5
22	cell cycle G2/M phase transition	GO:0044839	5
23	G2/M transition of mitotic cell cycle	GO:0000086	5
24	meiotic cell cycle process	GO:1903046	5
25	meiotic nuclear division	GO:0007126	5
26	posttranscriptional regulation of gene expression	GO:0010608	5
27	response to UV	GO:0009411	5
28	single-organism carbohydrate catabolic process	GO:0044724	5
29	chromatin remodeling	GO:0006338	4
30	gene silencing	GO:0016458	4
31	kidney development	GO:0001822	4
32	male meiosis I	GO:0007141	4
33	meiosis I	GO:0007127	4
34	microtubule-based movement	GO:0007018	4
35	negative regulation of secretion	GO:0051048	4
36	nucleosome organization	GO:0034728	4
37	protein-DNA complex subunit organization	GO:0071824	4
38	regulation of cyclin-dependent protein serine/threonine kinase activity	GO:0000079	4
39	regulation of translation	GO:0006417	4
40	antigen processing and presentation of exogenous peptide antigen via MHC class II	GO:0019886	3
41	antigen processing and presentation of peptide antigen via MHC class II	GO:0002495	3
42	antigen processing and presentation of peptide or polysaccharide antigen via MHC class II	GO:0002504	3
43	ATP-dependent chromatin remodeling	GO:0043044	3
44	cell aging	GO:0007569	3
45	cell division	GO:0051301	3
46	chromosome condensation	GO:0030261	3
47	chromosome segregation	GO:0007059	3
48	DNA conformation change	GO:0071103	3
49	DNA packaging	GO:0006323	3
50	gene silencing by RNA	GO:0031047	3
51	glycolytic process	GO:0006096	3
52	histone exchange	GO:0043486	3
53	internal peptidyl-lysine acetylation	GO:0018393	3
54	male meiosis	GO:0007140	3
55	negative regulation of binding	GO:0051100	3
56	piRNA metabolic process	GO:0034587	3
57	pyruvate metabolic process	GO:0006090	3
58	regulation of endothelial cell proliferation	GO:0001936	3
59	regulation of gene expression, epigenetic	GO:0040029	3
60	regulation of mitosis	GO:0007088	3
61	regulation of nuclear division	GO:0051783	3
62	spindle checkpoint	GO:0031577	3
63	astrocyte development	GO:0014002	2
64	ATP biosynthetic process	GO:0006754	2
65	attachment of spindle microtubules to kinetochore	GO:0008608	2
66	axonemal dynein complex assembly	GO:0070286	2
67	binding of sperm to zona pellucida	GO:0007339	2
68	calcium ion-dependent exocytosis	GO:0017156	2
69	CENP-A containing nucleosome assembly	GO:0034080	2
70	chromatin remodeling at centromere	GO:0031055	2
71	chromosome organization involved in meiosis	GO:0070192	2
72	cilium movement	GO:0003341	2
73	DNA alkylation	GO:0006305	2
74	DNA methylation	GO:0006306	2
75	DNA methylation involved in gamete generation	GO:0043046	2
76	DNA replication-independent nucleosome assembly	GO:0006336	2
77	DNA replication-independent nucleosome organization	GO:0034724	2
78	epithelial cilium movement	GO:0003351	2
79	G2 DNA damage checkpoint	GO:0031572	2
80	meiotic cell cycle	GO:0051321	2
81	membrane disassembly	GO:0030397	2
82	microtubule anchoring	GO:0034453	2
83	mitotic nuclear envelope disassembly	GO:0007077	2
84	mitotic sister chromatid segregation	GO:0000070	2
85	mitotic spindle organization	GO:0007052	2
86	NADP metabolic process	GO:0006739	2
87	negative regulation of cellular carbohydrate metabolic process	GO:0010677	2
88	negative regulation of gluconeogenesis	GO:0045721	2
89	negative regulation of Ras protein signal transduction	GO:0046580	2
90	negative regulation of small GTPase mediated signal transduction	GO:0051058	2
91	negative regulation of stem cell proliferation	GO:2000647	2
92	negative regulation of transcription factor import into nucleus	GO:0042992	2
93	neural tube development	GO:0021915	2
94	nuclear envelope disassembly	GO:0051081	2
95	nuclear envelope organization	GO:0006998	2
96	patterning of blood vessels	GO:0001569	2
97	positive regulation of glycolytic process	GO:0045821	2
98	positive regulation of translation	GO:0045727	2
99	positive regulation of translational initiation	GO:0045948	2
100	purine nucleoside monophosphate biosynthetic process	GO:0009127	2
101	purine nucleoside triphosphate biosynthetic process	GO:0009145	2
102	purine ribonucleoside monophosphate biosynthetic process	GO:0009168	2
103	purine ribonucleoside triphosphate biosynthetic process	GO:0009206	2
104	regulation of carbohydrate catabolic process	GO:0043470	2
105	regulation of cellular carbohydrate catabolic process	GO:0043471	2
106	regulation of chromosome segregation	GO:0051983	2
107	regulation of gluconeogenesis	GO:0006111	2
108	regulation of glycolytic process	GO:0006110	2
109	regulation of long-term neuronal synaptic plasticity	GO:0048169	2
110	regulation of translational initiation	GO:0006446	2
111	replicative senescence	GO:0090399	2
112	ribonucleoside triphosphate biosynthetic process	GO:0009201	2
113	sister chromatid segregation	GO:0000819	2
114	sperm-egg recognition	GO:0035036	2
115	spindle assembly checkpoint	GO:0071173	2
116	sulfur compound transport	GO:0072348	2
117	synaptonemal complex organization	GO:0070193	2

Cellular Component of Up-regulated Genes in PostMA

	Term	GO ID	Count
1	endoplasmic reticulum membrane	GO:0005789	7
2	mitochondrial membrane part	GO:0044455	3
3	ribosomal subunit	GO:0044391	3
4	ribosome	GO:0005840	3
5	cytosolic large ribosomal subunit	GO:0022625	2
6	microbody membrane	GO:0031903	2
7	peroxisomal membrane	GO:0005778	2
8	RNA polymerase II transcription factor complex	GO:0090575	2
9	secretory granule membrane	GO:0030667	2

Cellular Component of Up-regulated Genes in MA

	Term	GO ID	Count
1	cytosol	GO:0005829	19
2	endoplasmic reticulum membrane	GO:0005789	7
3	vacuolar part	GO:0044437	5
4	chromosomal region	GO:0098687	4
5	cullin-RING ubiquitin ligase complex	GO:0031461	3
6	cytoplasmic ribonucleoprotein granule	GO:0036464	3
7	heterochromatin	GO:0000792	3
8	integral component of mitochondrial membrane	GO:0032592	3
9	ribonucleoprotein granule	GO:0035770	3
10	condensed chromosome, centromeric region	GO:0000779	2
11	condensed nuclear chromosome, centromeric region	GO:0000780	2
12	Cul3-RING ubiquitin ligase complex	GO:0031463	2
13	pericentric heterochromatin	GO:0005721	2
14	proteasome core complex	GO:0005839	2
15	proteasome core complex, alpha-subunit complex	GO:0019773	2
16	SNARE complex	GO:0031201	2

Cellular Component of Up-regulated Genes in SCOS

	Term	GO ID	Count
1	extracellular vesicular exosome	GO:0070062	29
2	extracellular region	GO:0005576	21
3	extracellular space	GO:0005615	15
4	Golgi apparatus	GO:0005794	13
5	integral component of plasma membrane	GO:0005887	11
6	vacuolar part	GO:0044437	10
7	cell surface	GO:0009986	9
8	cytoplasmic vesicle part	GO:0044433	8
9	cytoplasmic vesicle membrane	GO:0030659	7
10	endocytic vesicle membrane	GO:0030666	7
11	vesicle membrane	GO:0012506	7
12	contractile fiber part	GO:0044449	6
13	endosomal part	GO:0044440	6
14	extracellular matrix	GO:0031012	6
15	MHC protein complex	GO:0042611	6
16	proteinaceous extracellular matrix	GO:0005578	6
17	axon	GO:0030424	5
18	coated vesicle membrane	GO:0030662	5
19	ER to Golgi transport vesicle membrane	GO:0012507	5
20	lysosomal lumen	GO:0043202	5
21	lysosomal membrane	GO:0005765	5
22	lysosome	GO:0005764	5
23	lytic vacuole	GO:0000323	5
24	transport vesicle membrane	GO:0030658	5
25	vacuolar lumen	GO:0005775	5
26	vacuolar membrane	GO:0005774	5
27	vacuole	GO:0005773	5
28	clathrin-coated endocytic vesicle membrane	GO:0030669	4
29	clathrin-coated vesicle membrane	GO:0030665	4
30	endocytic vesicle	GO:0030139	4
31	endosome membrane	GO:0010008	4
32	growth cone	GO:0030426	4
33	integral component of endoplasmic reticulum membrane	GO:0030176	4
34	integral component of lumenal side of endoplasmic reticulum membrane	GO:0071556	4
35	MHC class II protein complex	GO:0042613	4
36	protein-DNA complex	GO:0032993	4
37	site of polarized growth	GO:0030427	4
38	trans-Golgi network membrane	GO:0032588	4
39	cell cortex	GO:0005938	3
40	endosome lumen	GO:0031904	3
41	Golgi lumen	GO:0005796	3
42	postsynaptic density	GO:0014069	3
43	dendritic shaft	GO:0043198	2
44	extrinsic component of cytoplasmic side of plasma membrane	GO:0031234	2
45	MHC class I protein complex	GO:0042612	2
46	neuron projection membrane	GO:0032589	2
47	phagocytic vesicle membrane	GO:0030670	2
48	sarcomere	GO:0030017	2

Molecular Function of Up-regulated Genes in PostMA

	Term	GO ID	Count
1	bitter taste receptor activity	GO:0033038	5
2	taste receptor activity	GO:0008527	5
1	iron ion binding	GO:0005506	4
1	histone binding	GO:0042393	3
1	oxidoreductase activity, acting on CH-OH group of donors	GO:0016614	3
1	oxidoreductase activity, acting on the CH-OH group of donors, NAD or NADP as acceptor	GO:0016616	3
1	structural constituent of ribosome	GO:0003735	3
1	ferrous iron binding	GO:0008198	2
1	glutathione transferase activity	GO:0004364	2
1	R-SMAD binding	GO:0070412	2
1	steroid dehydrogenase activity	GO:0016229	2
1	steroid dehydrogenase activity, acting on the CH-OH group of donors, NAD or NADP as acceptor	GO:0033764	2
1	transferase activity, transferring alkyl or aryl (other than methyl) groups	GO:0016765	2

Molecular Function of Up-regulated Genes in MA

	Term	GO ID	Count
1	unfolded protein binding	GO:0051082	3
2	1-phosphatidylinositol-3-kinase activity	GO:0016303	2
3	acid-thiol ligase activity	GO:0016878	2
4	bitter taste receptor activity	GO:0033038	2
5	CoA-ligase activity	GO:0016405	2
6	frizzled binding	GO:0005109	2
7	glutathione transferase activity	GO:0004364	2
8	histone kinase activity	GO:0035173	2
9	intramolecular oxidoreductase activity	GO:0016860	2
10	ligase activity, forming carbon-sulfur bonds	GO:0016877	2
11	neurexin family protein binding	GO:0042043	2
12	phosphatidylinositol 3-kinase activity	GO:0035004	2
13	phosphatidylinositol kinase activity	GO:0052742	2
14	retinoic acid receptor binding	GO:0042974	2
15	SNARE binding	GO:0000149	2
16	syntaxin binding	GO:0019905	2
17	taste receptor activity	GO:0008527	2
18	threonine-type endopeptidase activity	GO:0004298	2
19	threonine-type peptidase activity	GO:0070003	2
20	transferase activity, transferring alkyl or aryl (other than methyl) groups	GO:0016765	2

Molecular Function of Up-regulated Genes in SCOS

	Term	GO ID	Count
1	zinc ion binding	GO:0008270	13
2	endopeptidase activity	GO:0004175	9
3	amide binding	GO:0033218	7
4	peptide binding	GO:0042277	7
5	phospholipid binding	GO:0005543	6
6	antigen binding	GO:0003823	5
7	growth factor activity	GO:0008083	5
8	metallopeptidase activity	GO:0008237	5
9	peptide antigen binding	GO:0042605	5
10	cargo receptor activity	GO:0038024	4
11	growth factor binding	GO:0019838	4
12	antioxidant activity	GO:0016209	3
13	collagen binding	GO:0005518	3
14	integrin binding	GO:0005178	3
15	MHC class II receptor activity	GO:0032395	3
16	NAD binding	GO:0051287	3
17	oxidoreductase activity, acting on peroxide as acceptor	GO:0016684	3
18	peroxidase activity	GO:0004601	3
19	phospholipase activity	GO:0004620	3
20	protein complex scaffold	GO:0032947	3
21	semaphorin receptor activity	GO:0017154	3
22	ADP binding	GO:0043531	2
23	AMP binding	GO:0016208	2
24	bitter taste receptor activity	GO:0033038	2
25	calcium-dependent cysteine-type endopeptidase activit	GO:0004198	2
26	calcium-dependent phospholipid binding	GO:0005544	2
27	calmodulin-dependent protein kinase activity	GO:0004683	2
28	glutamate receptor binding	GO:0035254	2
29	glutathione peroxidase activity	GO:0004602	2
30	glutathione transferase activity	GO:0004364	2
31	insulin receptor binding	GO:0005158	2
32	insulin-like growth factor binding	GO:0005520	2
33	mRNA 3'-UTR binding	GO:0003730	2
34	pattern recognition receptor activity	GO:0038187	2
35	phospholipase A2 activity	GO:0004623	2
36	proteoglycan binding	GO:0043394	2
37	scaffold protein binding	GO:0097110	2
38	signaling pattern recognition receptor activity	GO:0008329	2
39	taste receptor activity	GO:0008527	2
40	tau-protein kinase activity	GO:0050321	2

KEGG Pathway of Up-regulated Genes in PostMA

	Term	ID	Count
1	MicroRNAs in cancer	hsa05206	19
2	Taste transduction	hsa04742	6
3	ABC transporters	hsa02010	4
4	Butanoate metabolism	hsa00650	2
5	Steroid biosynthesis	hsa00100	2
6	Terpenoid backbone biosynthesis	hsa00900	2

KEGG Pathway of Up-regulated Genes in MA

	Term	ID	Count
1	MicroRNAs in cancer	hsa05206	17
2	Epstein-Barr virus infection	hsa05169	5
3	Oocyte meiosis	hsa04114	5
4	Cell cycle	hsa04110	4
5	Protein processing in endoplasmic reticulum	hsa04141	4
6	Antigen processing and presentation	hsa04612	3
7	Longevity regulating pathway - multiple species	hsa04213	3
8	Propanoate metabolism	hsa00640	3
9	Taste transduction	hsa04742	3
10	Steroid biosynthesis	hsa00100	2

KEGG Pathway of Up-regulated Genes in SCOS

	Term	ID	Count
1	MicroRNAs in cancer	hsa05206	12
2	Apoptosis	hsa04210	6
3	Epstein-Barr virus infection	hsa05169	6
4	Herpes simplex infection	hsa05168	6
5	Influenza A	hsa05164	6
6	Staphylococcus aureus infection	hsa05150	6
7	Antigen processing and presentation	hsa04612	5
8	Neurotrophin signaling pathway	hsa04722	5
9	Proteoglycans in cancer	hsa05205	5
10	Rheumatoid arthritis	hsa05323	5
11	Tuberculosis	hsa05152	5
12	Vascular smooth muscle contraction	hsa04270	5
13	Allograft rejection	hsa05330	4
14	Asthma	hsa05310	4
15	Autoimmune thyroid disease	hsa05320	4
16	Glutamatergic synapse	hsa04724	4
17	GnRH signaling pathway	hsa04912	4
18	Graft-versus-host disease	hsa05332	4
19	Hematopoietic cell lineage	hsa04640	4
20	Inflammatory bowel disease IBD)	hsa05321	4
21	Inflammatory mediator regulation of TRP channels	hsa04750	4
22	Insulin resistance	hsa04931	4
23	Intestinal immune network for IgA production	hsa04672	4
24	Leishmaniasis	hsa05140	4
25	Lysosome	hsa04142	4
26	Phagosome	hsa04145	4
27	Platelet activation	hsa04611	4
28	Serotonergic synapse	hsa04726	4
29	Thyroid hormone synthesis	hsa04918	4
30	Toxoplasmosis	hsa05145	4
31	Type I diabetes mellitus	hsa04940	4
32	Viral myocarditis	hsa05416	4
33	Adipocytokine signaling pathway	hsa04920	3
34	Arachidonic acid metabolism	hsa00590	3
35	Chronic myeloid leukemia	hsa05220	3
36	Gastric acid secretion	hsa04971	3
37	Glutathione metabolism	hsa00480	3
38	Longevity regulating pathway - multiple species	hsa04213	3
39	Long-term depression	hsa04730	3
40	Long-term potentiation	hsa04720	3
41	Ovarian steroidogenesis	hsa04913	3
42	PPAR signaling pathway	hsa03320	3
43	Protein digestion and absorption	hsa04974	3
44	Renal cell carcinoma	hsa05211	3
45	Renin secretion	hsa04924	3
46	Pentose phosphate pathway	hsa00030	2
47	Renin-angiotensin system	hsa04614	2

Cellular Component of Down-regulated Genes in PostMA

	Term	GO ID	Count
1	axonemal dynein complex	GO:0005858	4
2	axoneme part	GO:0044447	6
3	CatSper complex	GO:0036128	2
4	ciliary membrane	GO:0060170	2
5	ciliary part	GO:0044441	9
6	cytoplasmic dynein complex	GO:0005868	4
7	dynein complex	GO:0030286	4
8	extracellular region	GO:0005576	14
9	ion channel complex	GO:0034702	5
10	microtubule associated complex	GO:0005875	4
11	outer dense fiber	GO:0001520	2
12	plasma membrane region	GO:0098590	5
13	sperm fibrous sheath	GO:0035686	2
14	sperm part	GO:0097223	3
15	sperm principal piece	GO:0097228	2
16	transmembrane transporter complex	GO:1902495	6
17	transporter complex	GO:1990351	6
18	voltage-gated calcium channel complex	GO:0005891	2

Cellular Component of Down-regulated Genes in MA

	Term	GO ID	Count
1	cytoplasmic membrane-bounded vesicle	GO:0016023	8
2	sperm part	GO:0097223	8
3	ciliary part	GO:0044441	7
4	secretory granule	GO:0030141	6
5	cilium	GO:0005929	5
6	acrosomal vesicle	GO:0001669	4
7	motile cilium	GO:0031514	4
8	axoneme part	GO:0044447	3
9	centriole	GO:0005814	3
10	microtubule organizing center part	GO:0044450	3
11	sperm fibrous sheath	GO:0035686	3
12	sperm principal piece	GO:0097228	3
13	cAMP-dependent protein kinase complex	GO:0005952	2
14	germ cell nucleus	GO:0043073	2
15	male germ cell nucleus	GO:0001673	2
16	outer dense fiber	GO:0001520	2

Cellular Component of Down-regulated Genes in SCOS

	Term	GO ID	Count
1	nucleoplasm	GO:0005654	11
2	cilium	GO:0005929	8
3	DNA packaging complex	GO:0044815	7
4	motile cilium	GO:0031514	7
5	sperm part	GO:0097223	7
6	ciliary part	GO:0044441	6
7	DNA bending complex	GO:1990104	6
8	microtubule	GO:0005874	6
9	nucleosome	GO:0000786	6
10	protein-DNA complex	GO:0032993	6
11	acrosomal vesicle	GO:0001669	5
12	kinetochore	GO:0000776	5
13	microtubule cytoskeleton	GO:0015630	5
14	perinuclear region of cytoplasm	GO:0048471	5
15	secretory granule	GO:0030141	5
16	germ cell nucleus	GO:0043073	4
17	male germ cell nucleus	GO:0001673	4
18	chromatoid body	GO:0033391	3
19	cytoplasmic ribonucleoprotein granule	GO:0036464	3
20	microtubule associated complex	GO:0005875	3
21	microtubule organizing center part	GO:0044450	3
22	midbody	GO:0030496	3
23	ribonucleoprotein granule	GO:0035770	3
24	sperm fibrous sheath	GO:0035686	3
25	spindle	GO:0005819	3
26	axoneme	GO:0005930	2
27	axoneme part	GO:0044447	2
28	condensed chromosome kinetochore	GO:0000777	2
29	COPII vesicle coat	GO:0030127	2
30	cytoplasmic dynein complex	GO:0005868	2
31	dynein complex	GO:0030286	2
32	intercellular bridge	GO:0045171	2
33	P granule	GO:0043186	2
34	sperm flagellum	GO:0036126	2
35	sperm principal piece	GO:0097228	2
36	synaptonemal complex	GO:0000795	2
37	vesicle coat	GO:0030120	2

Molecular Function of Down-regulated Genes in PostMA

	Term	GO ID	Count
1	zinc ion binding	GO:0008270	10
2	metallopeptidase activity	GO:0008237	4
3	magnesium ion binding	GO:0000287	4
4	carboxylic ester hydrolase activity	GO:0052689	3
5	glycoprotein binding	GO:0001948	3
6	microtubule motor activity	GO:0003777	3
7	metalloendopeptidase activity	GO:0004222	3
8	ion gated channel activity	GO:0022839	2
9	lysozyme activity	GO:0003796	2
10	metalloexopeptidase activity	GO:0008235	2
11	phospholipid transporter activity	GO:0005548	2
12	RNA polymerase II core promoter sequence-specific DNA binding	GO:0000979	2
13	calcium activated cation channel activity	GO:0005227	2

Molecular Function of Down-regulated Genes in MA

	Term	GO ID	Count
1	protein serine/threonine kinase activity	GO:0004674	6
2	structural constituent of cytoskeleton	GO:0005200	4
3	hydrolase activity, acting on glycosyl bonds	GO:0016798	3
4	hydrolase activity, hydrolyzing O-glycosyl compounds	GO:0004553	3
5	lysozyme activity	GO:0003796	3
6	magnesium ion binding	GO:0000287	3
7	protein tyrosine phosphatase activity	GO:0004725	3
8	acetylglucosaminyltransferase activity	GO:0008375	2
9	protein kinase A binding	GO:0051018	2
10	protein tyrosine/serine/threonine phosphatase activity	GO:0008138	2

Molecular Function of Down-regulated Genes in SCOS

	Term	GO ID	Count
1	actin binding	GO:0003779	5
2	histone deacetylase binding	GO:0042826	3
3	small conjugating protein binding	GO:0032182	3
4	structural constituent of cytoskeleton	GO:0005200	3
5	ubiquitin binding	GO:0043130	3
6	chaperone binding	GO:0051087	2
7	clathrin binding	GO:0030276	2
8	mRNA 3'-UTR binding	GO:0003730	2
9	oxidoreductase activity, acting on the aldehyde or oxo g	GO:0016620	2
10	polyubiquitin binding	GO:0031593	2
11	proteasome binding	GO:0070628	2
12	protein tyrosine/serine/threonine phosphatase activity	GO:0008138	2
13	translation activator activity	GO:0008494	2
14	translation regulator activity	GO:0045182	2
15	translation regulator activity, nucleic acid binding	GO:0090079	2

KEGG Pathway of Down-regulated Genes in PostMA

	Term	ID	Count
1	Huntington's disease	hsa05016	4
2	Thyroid hormone synthesis	hsa04918	2

KEGG Pathway of Down-regulated Genes in MA

	Term	ID	Count
1	Metabolic pathways	hsa01100	6

KEGG Pathway of Down-regulated Genes in SCOS

	Term	ID	Count
1	Glycolysis +		4
2	Carbon metabolism	hsa01200	3
3	Cell cycle	hsa04110	3
4	Glucagon signaling pathway	hsa04922	3
5	Glycolysis / Gluconeogenesis	hsa00010	3
6	Oocyte meiosis	hsa04114	3
7	Progesterone-mediated oocyte maturation	hsa04914	3
8	Biosynthesis of amino acids	hsa01230	2
9	Central carbon metabolism in cancer	hsa05230	2
10	Propanoate metabolism	hsa00640	2
11	Pyruvate metabolism	hsa00620	2
